# Estimating the distribution of morbidity and mortality of childhood diarrhea, measles, and pneumonia by wealth group in low- and middle-income countries

**DOI:** 10.1186/s12916-018-1074-y

**Published:** 2018-07-04

**Authors:** Angela Y. Chang, Carlos Riumallo-Herl, Joshua A. Salomon, Stephen C. Resch, Logan Brenzel, Stéphane Verguet

**Affiliations:** 1 Department of Global Health and Population, Harvard T.H. Chan School of Public Health, Boston, MA USA; 2 Institute for Health Metrics and Evaluation, University of Washington, Seattle, WA USA; 3 Erasmus School of Economics, Erasmus University of Rotterdam, Rotterdam, The Netherlands; 4 Department of Medicine, Stanford University School of Medicine, Stanford, CA USA; 5 Center for Health Decision Science, Harvard T.H. Chan School of Public Health, Boston, MA USA; 6 Bill & Melinda Gates Foundation, Seattle, WA USA

**Keywords:** Distributional benefits, Vaccines, Equity, Risk factors, Measles vaccine, Pneumococcal conjugate vaccine, Rotavirus vaccine

## Abstract

**Background:**

Equitable access to vaccines has been suggested as a priority for low- and middle-income countries (LMICs). However, it is unclear whether providing equitable access is enough to ensure health equity. Furthermore, disaggregated data on health outcomes and benefits gained across population subgroups are often unavailable. This paper develops a model to estimate the distribution of childhood disease cases and deaths across socioeconomic groups, and the potential benefits of three vaccine programs in LMICs.

**Methods:**

For each country and for three diseases (diarrhea, measles, pneumonia), we estimated the distributions of cases and deaths that would occur across wealth quintiles in the absence of any immunization or treatment programs, using both the prevalence and relative risk of a set of risk and prognostic factors. Building on these baseline estimates, we examined what might be the impact of three vaccines (first dose of measles, pneumococcal conjugate, and rotavirus vaccines), under five scenarios based on different sets of quintile-specific immunization coverage and disease treatment utilization rates.

**Results:**

Due to higher prevalence of risk factors among the poor, disproportionately more disease cases and deaths would occur among the two lowest wealth quintiles for all three diseases when vaccines or treatment are unavailable. Country-specific context, including how the baseline risks, immunization coverage, and treatment utilization are currently distributed across quintiles, affects how different policies translate into changes in cases and deaths distribution.

**Conclusions:**

Our study highlights several factors that would substantially contribute to the unequal distribution of childhood diseases, and finds that merely ensuring equal access to vaccines will not reduce the health outcomes gap across wealth quintiles. Such information can inform policies and planning of programs that aim to improve equitable delivery of healthcare services.

**Electronic supplementary material:**

The online version of this article (10.1186/s12916-018-1074-y) contains supplementary material, which is available to authorized users.

## Background

With the ambitious goals of ending extreme poverty and fighting against inequity in low- and middle-income countries (LMICs) [1], international agencies, such as the World Bank, have advocated for improving access to essential services, such as healthcare and education, for the lowest 40% of income earners [2]. Policymakers in LMICs, where often vast inequities in health exist, are interested in enhancing equity for both ethical and political reasons [3, 4].

Vaccine programs have been recognized as one of the most successful interventions in improving population health worldwide. Efforts put forward by local governments and international agencies have contributed to raising childhood vaccine coverage in the last decade [5], though high child mortality is still observed in LMICs, with approximately 5.9 million under-five deaths in 2015 [6]. More recently, the Global Vaccine Action Plan and Gavi, the Vaccine Alliance, both listed equitable access to vaccination as their top priorities [7–9]. However, it is unclear whether ensuring equitable access to vaccines would lead to health equity, which we define as equality of health outcomes across population subgroups. To answer this question, one needs to compare how disease burden is distributed across subgroups before and after the introduction of vaccines. However, such data on disease burden by socioeconomic strata before and after vaccines are not available empirically, making it difficult to design equitable policies for populations. Furthermore, while vaccines prevent diseases, access to treatment is needed in preventing deaths among people who already contracted the disease, which is often another source of inequity across population subgroups. The objective of this work is therefore to introduce an analytical approach to estimate the distribution of childhood disease cases and deaths and the benefits of vaccines and treatments by socioeconomic group.

## Methods

We examined three major childhood vaccine-preventable diseases – measles, pneumonia, and diarrhea, selected as they represented 23% of deaths as of 2015 (1%, 13%, and 9%, respectively) occurring among under-five children in LMICs [6], and have well-established sets of risk and prognostic factors. We studied the three corresponding vaccines (and the treatment of the diseases they prevent), namely measles vaccine (routine first dose, MCV1), rotavirus vaccine (RV, against rotavirus diarrhea), and pneumococcal conjugate vaccine (PCV, against pneumococcal pneumonia).

**Fig. 1:**
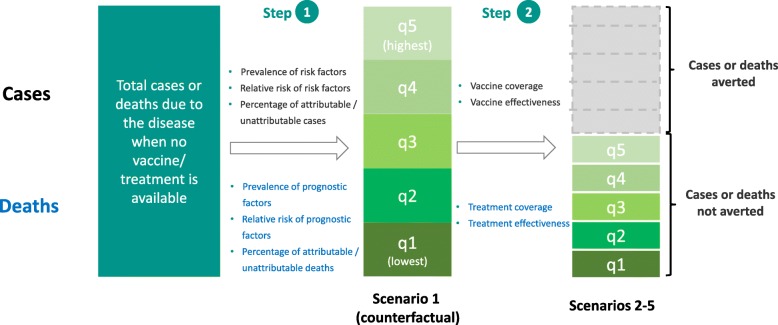
Flow diagram of the analytical approach to estimate the distribution of cases/deaths of measles, diarrhea, and pneumonia under scenarios with and without vaccines/treatment

The approach is based on the concept of population attributable fraction (PAF) [10]. Using PAF, we quantified the contribution of sets of risk and prognostic factors, defined as behaviors and characteristics of an individual that can be used to estimate the likelihood of contracting and dying from each disease. The proportion of cases and deaths that could be attributed to the exposure of selected sets of risk and prognostic factors are classified as ‘attributable’, and the remaining as ‘unattributable’ cases and deaths. Our model takes advantage of the differences in the prevalence of risk and prognostic factors across wealth strata to estimate the distribution of cases and deaths. A flow diagram outlining each step is presented in Fig. 1.

### Analytical structure

#### Cases

For each risk factor among a total of *n* risk factors, we defined w_j_ as the relative weight assigned to risk factor j, and RR_j_ as the relative risk of risk factor j: ${\mathrm{w}}_{\mathrm{j}}=\frac{{\mathrm{RR}}_{\mathrm{j}}}{\sum_{\mathrm{k}=1}^{\mathrm{n}}{\mathrm{RR}}_{\mathrm{k}}}$. Subsequently, the proportion of attributable cases (Ac_i_) occurring in wealth quintile i could be estimated as:

1 $$ {\mathrm{Ac}}_{\mathrm{i}}=\frac{\sum_{\mathrm{j}=1}^{\mathrm{n}}{\mathrm{w}}_{\mathrm{j}}{\mathrm{P}}_{\mathrm{i}\mathrm{j}}}{\sum_{\mathrm{m}=1}^5{\sum}_{\mathrm{j}=1}^{\mathrm{n}}{\mathrm{w}}_{\mathrm{j}}{\mathrm{P}}_{\mathrm{m}\mathrm{j}}} $$

where P_ij_ is the prevalence of risk factor j in wealth quintile i (Step 1 in Fig. 1). In other words, when vaccines are not available, we would expect Ac_i_% of attributable proportion of cases to occur in quintile i.

In the presence of a vaccine program, we added quintile-specific vaccine coverage (V_i_) and vaccine efficacy (E, assumed constant for simplicity) to estimate the cases not averted by the program (Step 2 in Fig. 1), using a simple static model:

2 $$ {\mathrm{Ac}}_{\mathrm{v},\mathrm{i}}=\frac{\left(1-{\mathrm{V}}_{\mathrm{i}}\mathrm{E}\right){\sum}_{\mathrm{j}=1}^{\mathrm{n}}{\mathrm{w}}_{\mathrm{j}}{\mathrm{P}}_{\mathrm{i}\mathrm{j}}}{\sum_{\mathrm{m}=1}^5{\sum}_{\mathrm{j}=1}^{\mathrm{n}}{\mathrm{w}}_{\mathrm{j}}{\mathrm{P}}_{\mathrm{m}\mathrm{j}}\left(1-{\mathrm{V}}_{\mathrm{m}}\mathrm{E}\right)} $$

In other words, the distribution of attributable cases would depend on the prevalence of the risk factors, the relative risks of the risk factors, vaccine coverage, and vaccine efficacy. We adopted a simple approach to understand the impact of different combinations of factors in a straightforward way, though we acknowledge the limitations of a static model, including the absence of herd immunity and dynamic transmissions [11, 12].

#### Deaths

Whether a death occurs would depend not only on the prevalence and relative risk of each prognostic factor, which impacts the underlying risk of mortality in each quintile, but also on whether treatment is accessible and effective. Similar to the step above, we first assigned weights to each prognostic factor contributing to disease mortality: ${\mathrm{u}}_{\mathrm{l}}=\frac{{\mathrm{RR}}_{\mathrm{l}}}{\sum_{\mathrm{k}=1}^{\mathrm{p}}{\mathrm{RR}}_{\mathrm{k}}}$, where u_l_ is the relative weight assigned to prognostic factor l and RR_l_ is the relative risk of prognostic factor l. We estimated the proportion of attributable deaths that would occur in each quintile i when treatment is available as:

3 $$ {\mathrm{Ad}}_{\mathrm{t},\mathrm{i}}=\frac{\left.\Big(1-{\mathrm{a}}_{\mathrm{i}}\mathrm{Eff}\right){\sum}_{\mathrm{l}=1}^{\mathrm{p}}{\mathrm{u}}_{\mathrm{l}}{\mathrm{P}}_{\mathrm{i}\mathrm{l}}\ }{\sum_{\mathrm{m}=1}^5{\sum}_{\mathrm{l}=1}^{\mathrm{p}}{\mathrm{u}}_{\mathrm{l}}{\mathrm{P}}_{\mathrm{m}\mathrm{l}}\ \left.\Big(1-{\mathrm{a}}_{\mathrm{m}}\mathrm{Eff}\right)} $$

where P_il_ is the prevalence of prognostic factor l in quintile i, a_i_ is the proportion of cases in quintile i for whom treatment was sought, and Eff is the treatment effectiveness. For simplicity, we assumed treatment effectiveness to be constant across quintiles, but this assumption could be relaxed if data on quintile-specific effectiveness were available.

For the unattributable portion, we assumed that the cases and deaths before accessing vaccines or treatment were distributed equally across quintiles. In the presence of vaccine or treatment programs, the distribution of unattributable cases/deaths would depend on the gradients of vaccine or treatment coverage rates, using the simple static formulation:

4 $$ {\mathrm{UAc}}_{\mathrm{v},\mathrm{i}}=\frac{\left(1-{\mathrm{V}}_{\mathrm{i}}\mathrm{E}\right)}{\sum_{m=1}^5\left(1-{\mathrm{V}}_{\mathrm{m}}\mathrm{E}\right)} $$

5 $$ {\mathrm{UAd}}_{\mathrm{t},\mathrm{i}}=\frac{\left.\Big(1-{\mathrm{a}}_{\mathrm{i}}\mathrm{Eff}\right)\ }{\sum_{m=1}^5\left.\Big(1-{\mathrm{a}}_{\mathrm{m}}\mathrm{Eff}\right)\ } $$

Thus, formally, for each disease:

6 $$ {\mathrm{C}}_{\mathrm{i}}={\mathrm{Ac}}_{\mathrm{i}}\times {\mathrm{TC}}_a+{\mathrm{UAc}}_{\mathrm{i}}\times {\mathrm{TC}}_{ua} $$

7 $$ {\mathrm{D}}_{\mathrm{i}}={\mathrm{Ad}}_{\mathrm{i}}\times {\mathrm{TD}}_a+{\mathrm{UAd}}_{\mathrm{i}}\times {\mathrm{TD}}_{ua} $$

where C_i_ and D_i_ are the proportion of all cases and deaths in wealth quintile i, TC_*a*_ and TC_*ua*_ are the proportion of all cases attributable and unattributable to risk factors, and TD_*a*_ and TD_*ua*_ are the proportion of all deaths attributable and unattributable to prognostic factors.

A summary of all the symbols used and corresponding parameters is summarized in Table 1.

**Table 1:** List of symbols used and corresponding parameters and data sources

Parameter definition	Symbol	Parameter value	Data source
Proportion of attributable cases and deaths in quintile i	Ac_i_, Ad_i_	See Additional file 1: Section V	Authors’ estimation
Proportion of attributable cases and deaths averted by vaccine/treatment in quintile i	Ac_v,i_, Ad_t,i_	See Additional file 1: Section V	Authors’ estimation
Proportion of all cases and deaths attributable to risk and prognostic factors	TC_*a*_, TD_*a*_	See Additional file 1: Section IV	[14]
Proportion of all cases and deaths unattributable to risk and prognostic factors	TC_*ua*_, TD_*ua*_	See Additional file 1: Section IV	[14]
Disease-specific relative risk of risk factor j or prognostic factor l	RR_j_, RR_l_	See Table 2	–
Relative weight assigned to risk factor j or prognostic factor l	w_j_, u_l_	–	Authors’ estimation
Prevalence of risk factor j or prognostic factor l in quintile i	P_ij_, P_il_	See Table 2	–
Vaccine effectiveness	E	MCV1: 0.85 (95% CI 0.83–0.87) PCV: 0.06 (95% CI 0.03–0.09) for clinically diagnosed pneumonia RV: 0.14 (95% CI 0.00–0.31) for diarrhea	[17–19]
Healthcare treatment efficacy in reducing mortality	Eff	Measles: 0.62 (95% CI 0.19–0.82) Pneumonia: 0.70 (95% CI 0.52–0.82) Diarrhea: 0.93 (95% CI 0.83–0.98)	[19, 32, 33]

*CI* confidence interval, *MCV1* measles vaccine first dose, *PCV* pneumococcal conjugate vaccine, *RV* rotavirus vaccine

### Data sources

LMICs with Demographic and Health Surveys (DHS) available after 2010 were selected (the full list is available in Additional file 1: Section I). We first searched the literature for risk and prognostic factors with the highest relative risks for diarrhea, measles, and pneumonia. For each factor, we also checked its availability by quintile-specific prevalence rate in the DHS [13]. Risk factors that did not match with DHS variables, had poor data quality, were not studied in systematic reviews, or had poor variable definition were excluded (listed in Table 2). We shortlisted four to five factors per disease such as childhood malnutrition indicators, non-exclusive breastfeeding, and vitamin A deficiency (Table 2, for more details see Additional file 1: Section II).

**Table 2:** Risk and prognostic factors, and relative risks for morbidity and mortality, for measles, diarrhea, and pneumonia

Disease-vaccine pair	Risk factors of morbidity (relative risk magnitude is indicated in parentheses)	Prognostic factors of mortality (relative risk magnitude is indicated in parentheses)	Sources
Measles – Measles vaccine	• Wasting: z-score < –3 SD (38.0, 95% CI 5.1–200.7); –2 SD < z-score < –3 SD (8.5, 95% CI 1.3–42.9) • Maternal education (3.2)^a^ • Having no other children vaccinated at home (3.0)^a^ • Underweight: z-score < –3 SD (5.7, 95% CI 1.8–12.4); –2 SD < z-score < –3 SD (2.5, 95% CI 1.3–5.1) • Stunting: z-score < –3 SD (2.5, 95% CI 1.1–6.6); –2 SD < z-score < –3 SD (1.5, 95% CI 1.0–3.3) • Vitamin A deficiency (2.4, 95% CI 1.6–3.5)	• Wasting: z-score < –3 SD (38.0, 95% CI 5.1–200.7); –2 SD < z-score < –3 SD (8.5, 95% CI 1.3–42.9) • Underweight: z-score < –3 SD (5.7, 95% CI 1.8–12.4); –2 SD < z-score < –3 SD (2.5, 95% CI 1.3–5.1) • Stunting: z-score < –3 SD (2.5, 95% CI 1.1–6.6); –2 SD < z-score < –3 SD (1.5, 95% CI 1.0–3.3) • Vitamin A deficiency (2.4, 95% CI 1.6–3.5) • More than one child (1.8)^a^ • Age at infection (NA)^a^ • Secondary (versus primary) exposure (NA)^a^ • Infection with complication (NA)^a^ • Overcrowding (NA)^a^ • Intensity of exposure and patterns of disease transmission (NA)^a^	[14, 27, 34, 35]
Pneumonia – Pneumococcal conjugate vaccine	• Wasting: z-score < –3 SD (116.7, 95% CI 25.2–179.3); –2 SD < z-score < –3 SD (25.6, 95% CI 6.1–39.7) • Non-exclusive breastfeeding: None (4.5, 95% CI 1.0–18.3); Partial (5.4, 95% CI 1.0–20.9); Predominant (1.8, 95% CI 1.4–2.3) • Underweight: z-score < –3 SD (2.1, 95% CI 1.8–2.7); –2 SD < z-score < –3 SD (1.3, 95% CI 1.2–1.4) • Stunting: z-score < –3 SD (1.9); –2 SD < z-score < –3 SD (1.2)^b^ • Zinc deficiency (1.8)^a^ • Vitamin A deficiency (1.6, 95% CI 1.2–2.0) • Low birth weight (< 2500 g) (1.4) • Exposed to household air pollution (1.4)^a^ • Crowding (more than five people per household) (1.4)^a^ • Secondhand smoke (1.2)^a^ • Parental literacy level (NA)^a^	• Wasting: z-score < –3 SD (116.7, 95% CI 25.2–179.3); –2 SD < z-score < –3 SD (25.6, 95% CI 6.1–39.7) • Non-exclusive breastfeeding: None (51.4, 95% CI 2.1–325.9); Partial (2.8, 95% CI 1.3–5.2); Predominant (1.9, 95% CI 1.0–4.1) • Zinc deficiency (1.7)^a^ • Underweight: z-score < –3 SD (2.1, 95% CI 1.8–2.7); –2 SD < z-score < –3 SD (1.3, 95% CI 1.2–1.4) • Stunting: z-score < –3 SD (1.9, 95% CI 1.0–3.6); –2 SD < z-score < –3 SD (1.2, 95% CI 1.0–1.7) • Vitamin A deficiency (1.6, 95% CI 1.2–2.0) • Secondhand smoke (1.2)^a^	[14, 27, 28, 36, 37]
Diarrhea – Rotavirus vaccine	• Wasting: z-score < –3 SD (105.8, 95% CI 42.2–158.0); –2 SD < z-score < –3 SD (23.3, 95% CI 8.9–35.9) • Unsafe water source (11.2)^a^ • Unsafe sanitation (3.2, 95% CI 2.8–3.7) • Mothers handwashing not practiced at critical time (2.2)^a^ • Underweight: z-score < –3 SD (2.3, 95% CI 2.1–2.8); –2 SD < z-score < –3 SD (1.2, 95% CI 1.1–1.5) • Non-exclusive breastfeeding: None (2.2, 95% CI 1.5–3.2); Partial (1.5, 95% CI 1.0–2.3); Predominant (1.2, 95% CI 1.0–1.7) • Stunting: z-score < –3 SD (1.9, 95% CI 1.3–2.7); –2 SD < z-score < –3 SD (1.2, 95% CI 1.1–1.5) • Zinc deficiency (1.9)^a^ • No hand washing with soap (1.7)^c^ • Maternal literacy (1.7)^a^ • Having more than two children aged under five years (1.7)^a^	• Wasting: z-score < –3 SD (105.8, 95% CI 42.2–158.0); –2 SD < z-score < –3 SD (23.3, 95% CI 8.9–35.9) • Unsafe water source (11.2)^a^ • Non-exclusive breastfeeding: None (9.7, 95% CI 2.4–28.1); Partial (3.9, 95% CI 1.5–8.3); Predominant (2.1, 95% CI 1.0–4.6) • Unsafe sanitation (3.2, 95% CI 2.8–3.7) • Underweight: z-score < –3 SD (2.3, 95% CI 2.1–2.8); –2 SD < z-score < –3 SD (1.2, 95% CI 1.1–1.5) • Zinc deficiency (2.0)^a^ • Stunting: z-score < –3 SD (1.9, 95% CI 1.3–2.7); –2 SD < z-score < –3 SD (1.2, 95% CI 1.1–1.5) • No handwashing with soap (1.7)^c^ • Vitamin A deficiency (1.5)^b^	[14, 27, 36, 38, 39]

^a^Risk factors not included in the analysis due to Demographic and Health Survey data unavailable by wealth quintile

^b^Risk factors not included in the analysis due to lower relative risk

^c^Risk factors not included in the analysis due to poor data quality and/or poor variable definition

*CI* confidence interval, *NA* not available, *SD* standard deviation

We collected country-specific data on the proportion of disease-specific cases and deaths that can be attributable to sets of risk and prognostic factors. The average percentage of attributable cases (deaths) in the selected countries were 73% (76%) for measles, 92% (94%) for pneumonia, and 95% (97%) for diarrhea [14, 15]. In other words, under-five cases and deaths for those diseases were largely attributable to the selected risk and prognostic factors.

### Outcomes and scenarios

Per disease, we first estimated the distribution of cases/deaths across quintiles without immunization/treatment. Second, we assessed the impact of vaccine or treatment programs on this distribution under five scenarios. For cases, Scenario 1 (S1) assumes zero vaccine coverage, which captures today’s status in many LMICs for PCV and RV (not MCV1); in other words, we estimate the counterfactual scenario of how disease cases (starting with 100% cases) would have been distributed across quintiles when vaccines are not available. S2 incorporates differences in current vaccine coverage across quintiles. We used vaccine coverage estimates from Gavi for 2016 [16]. To obtain quintile-specific MCV1 coverage, we multiplied these estimates with the quintile-specific MCV1 coverage rates obtained from the DHS. For PCV and RV, 6 and 14 out of the 41 countries had not introduced PCV and RV, respectively. Among countries that did introduce the vaccines in 2016, the DHS did not report vaccine coverage rates by quintiles. To estimate quintile-specific coverage rates for PCV and RV for all countries, we first calculated the ratios of quintile-specific three- and two-dose diphtheria-tetanus-pertussis vaccine coverage rates and the national average in the DHS (since PCV and RV match the latter’s three- and two-dose schedules, respectively), and assumed that these ratios applied to PCV and RV. We assumed that these vaccines only benefitted children who received all the recommended doses (three for PCV and two for RV), and not those who were partially vaccinated, even though partial vaccination may provide some degree of protection. To examine the effect of redistributing existing doses on health equity, S3 maintains the same total number of vaccines available in each country as S2, but distributes an equal number of doses to each quintile. In other words, S3 measures how much equity gain a country would see purely through redistribution without purchasing any additional vaccines. S4 assumes that vaccine coverage per quintile is proportional to the morbidity risk assigned to that quintile. In other words, those with a higher risk of getting the disease have a higher probability of receiving the vaccine. Finally, to examine the effect of targeting risk factors instead of vaccine coverage, S5 first reduces the baseline morbidity risks of all quintiles to the lowest risk level observed in the country (i.e., lowest prevalence of each risk factor), such that all quintiles have the same number of cases, and then applies the current quintile-specific vaccine coverage.

For deaths, S1 examines the distribution of deaths under the hypothetical scenario when no treatment is available. S2 reflects the differences in the current treatment utilization rates across wealth quintiles and how these would lead to unequal distributions. We used the percentage of those seeking care from a health provider for different conditions from the DHS as treatment coverage rates, namely the percentage who sought care among those with acute respiratory infection (for pneumonia) and diarrhea; for measles, the average of the proportions among those with acute respiratory infection and diarrhea was used. S3 assumes equal treatment coverage (at the national average) across all quintiles. S4 assumes that quintile-specific treatment coverage is proportional to the underlying mortality risk, i.e., those with a higher risk of dying from the disease have a higher probability of receiving treatment. Finally, S5 assumes that the prevalence of all prognostic factors are set at the lowest level observed in the country for all quintiles and applies the most recent DHS-reported quintile-specific treatment coverage rates.

For each scenario, we calculated (1) the proportion of cases and deaths by quintile, and (2) the change in the distribution of remaining cases and deaths. For (1), we assumed a starting point of 100% of cases or deaths when no vaccine or treatment programs were present and calculated the reduction in the total burden under each scenario. For (2), we explored the area under the curve (AUC) of the cumulative percentage of cases (or deaths) by quintile, and calculated the changes in the AUC per scenario. For example, an increase in the AUC after implementation of a vaccine program suggests that, among the remaining cases not averted by the program, a higher percentage of cases would be among the poorer quintiles compared to prior vaccine introduction. A detailed description of the methods, including an illustration of how the distributions and the AUCs were estimated, can be found in Additional file 1: Section IV.

We calculated 95% uncertainty ranges for our estimates using Monte Carlo simulations (*n* = 1000 draws), while varying the relative risks of risk and prognostic factors, vaccine efficacy, and treatment effectiveness simultaneously using truncated normal distributions with the inputs’ means and standard deviations.

### Sensitivity analyses

Two sets of sensitivity analyses were conducted. First, we accounted for the differences in the sizes of the under-five population in each quintile by adjusting the number of cases and deaths with the quintile-specific total fertility rate (TFR). Second, instead of assuming that unattributable cases and deaths were distributed equally across quintiles, we used the ratios of quintile-specific under-five mortality rates to the national average for adjustment.

## Results

Estimates for all countries are available in Additional file 1: Section IV. To illustrate, we display our findings for each disease for three populous country examples – Nigeria, Pakistan, and Ethiopia.

### Measles cases

**Fig. 2:**
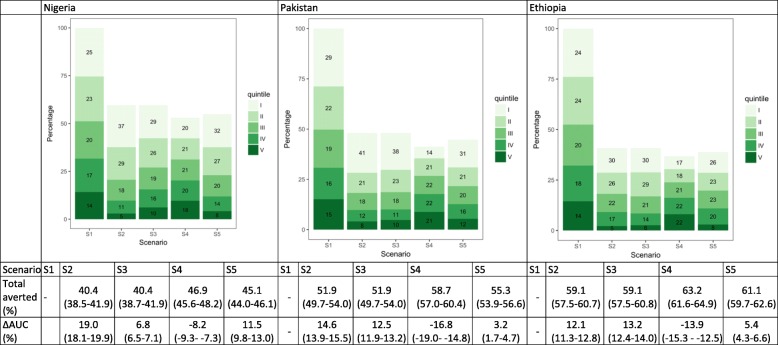
Distribution of measles cases by wealth quintile and scenario in Nigeria, Pakistan, and Ethiopia. The numbers in the green boxes represent the percentage of cases in each wealth quintile. Wealth quintiles: I = Lowest, II = Lower, III = Middle, IV = Higher, V = Highest. AUC = area under the curve. ∆ AUC: Percent change in AUC compared to Scenario 1 (S1). 95% uncertainty ranges are indicated in parentheses. (S1): no vaccine program available; S2: current vaccine program; S3: total number of vaccines from S2 distributed equally across quintiles; S4: vaccine coverage proportional to quintile-specific baseline morbidity risks; S5: equal baseline morbidity risk with current quintile-specific vaccine coverage

Under the hypothetical scenario of no vaccines (S1), more measles cases would occur among the lower wealth quintiles. In Nigeria, Pakistan, and Ethiopia, the two lowest quintiles would account for 48.8% (95% uncertainty range 48.0–50.0), 50.3% (48.9–51.8), and 47.6% (46.5–48.6) of all cases, in comparison to 31.7% (30.7–32.4), 30.7% (29.3–32.2), and 32.1% (31.4–32.9) in the two highest quintiles (S1, Fig. 2). With MCV1 (S2), in which coverage is greater among the higher quintiles, a larger proportion of cases would be averted in these quintiles. Compared to S1, AUC would increase by 19.0% (18.0–19.8), 14.6% (13.8–15.4), and 12.1% (11.4–12.8), respectively, in the three countries, suggesting that more measles cases would occur among the lowest two quintiles than the highest two. S3 assumes equal number of MCV1 across quintiles, and thus the same number of cases would be averted per quintile, leading to a larger AUC than for S1 (since the lowest quintiles would have more remaining cases because of the unequal distribution of baseline morbidity risk ex ante), but smaller increases in AUC than for S2 (more equal distribution of the vaccine). This effect would be greater in countries with greater inequalities in vaccine coverage rates, such as Nigeria (16% in the lowest quintile versus 93% in the highest quintile) and Pakistan (36% versus 86%). Distributing MCV1 proportionally to quintile-specific risks (S4) would substantially reduce the unequal distribution (decrease in AUC) for all three countries. In S5, we estimated a larger proportion of cases being averted (compared to S2 and S3) in Nigeria and Pakistan, suggesting that addressing the underlying morbidity is an effective strategy to reduce overall burden in countries with greater unequal risk distribution.

### Pneumonia cases

**Fig. 3:**
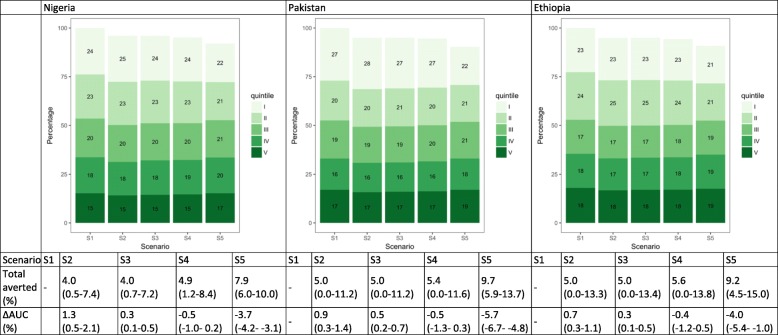
Distribution of pneumonia cases by wealth quintile and scenario in Nigeria, Pakistan, and Ethiopia. The numbers in the green boxes represent the percentage of cases in each wealth quintile. Wealth quintiles: I = Lowest, II = Lower, III = Middle, IV = Higher, V = Highest. AUC = area under the curve. ∆ AUC: Percent change in AUC compared to Scenario 1 (S1). 95% uncertainty ranges are indicated in parentheses. (S1): no vaccine program available; S2: current vaccine program; S3: total number of vaccines from S2 distributed equally across quintiles; S4: vaccine coverage proportional to quintile-specific baseline morbidity risks; S5: equal baseline morbidity risk with current quintile-specific vaccine coverage

Without vaccines, pneumonia cases are estimated to be concentrated among the lower quintiles (S1) (Fig. 3). Even though the differences in PCV coverage across quintiles would be large (for example, an average of 21% in the lowest two quintiles versus close to 100% in the highest two quintiles in Nigeria), the introduction of PCV would have a limited effect on the distribution of pneumonia cases because of its low vaccine efficacy (6% for clinically diagnosed pneumonia [17]). Under the current vaccine coverage scenario (S2), we estimate small but positive increases in AUC due to the unequal distribution of vaccines. Even after equalizing dosage distribution, minimal changes would occur in both the number of cases averted and the relative distribution of the remaining cases (S3). Distributing PCV in proportion to baseline risks would lead to a slightly greater number of total cases averted, but the reductions in AUC would not be significant (S4). Instead, equalizing baseline risks first and then applying current coverage rates would lead to small improvements in both the number of averted cases and AUC in all three countries.

### Diarrhea cases

**Fig. 4:**
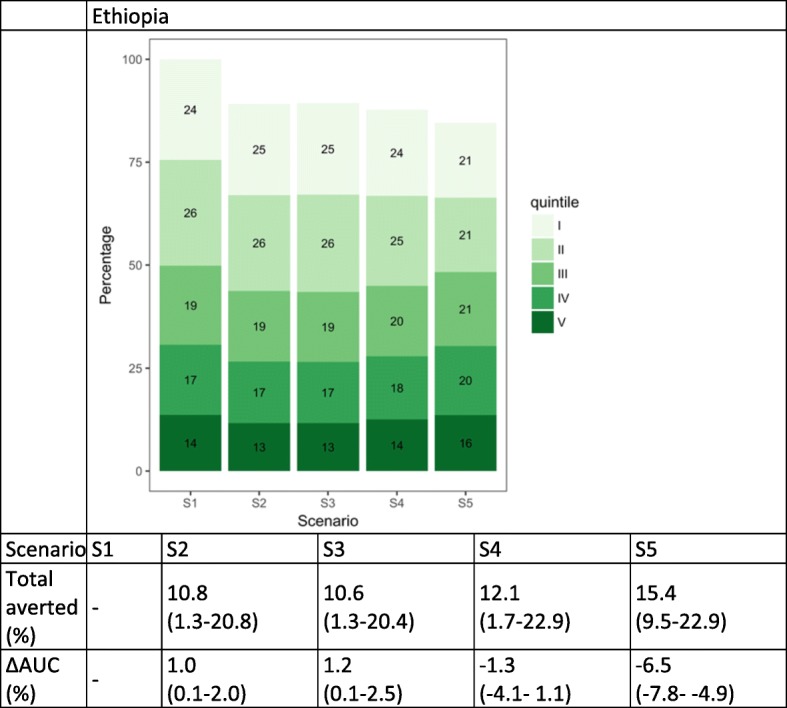
Distribution of diarrhea cases by wealth quintile and scenario in Ethiopia. The numbers in the green boxes represent the percentage of cases in each wealth quintile. Wealth quintiles: I = Lowest, II = Lower, III = Middle, IV = Higher, V = Highest. AUC = area under the curve. ∆ AUC: Percent change in AUC compared to Scenario 1 (S1). 95% uncertainty ranges are indicated in parentheses. (S1): no vaccine program available; S2: current vaccine program; S3: total number of vaccines from S2 distributed equally across quintiles; S4: vaccine coverage proportional to quintile-specific baseline morbidity risks; S5: equal baseline morbidity risk with current quintile-specific vaccine coverage

Among the three countries, only Ethiopia introduced RV in 2016 (Fig. 4). Similar to PCV, the effect of RV on the distribution of diarrhea cases is smaller than MCV1 due to lower vaccine efficacy (14% for all diarrhea [18]). Current vaccine coverage (S2) or providing equal vaccine coverage (S3) would result in a small increase in AUC as higher proportions of cases would be averted among the higher wealth quintiles. Equalizing the underlying risks (S5), on the other hand, would both increase the size of the total cases averted and result in more equal case distribution (decrease in AUC).

### Measles deaths

**Fig. 5:**
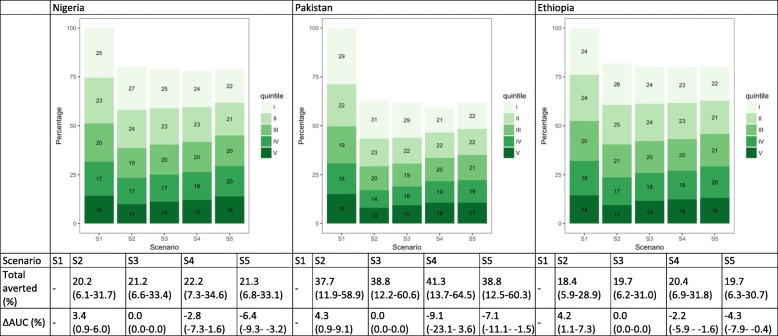
Distribution of measles deaths by wealth quintile and scenario in Nigeria, Pakistan, and Ethiopia. The numbers in the green boxes represent the percentage of deaths in each wealth quintile. Wealth quintiles: I = Lowest, II = Lower, III = Middle, IV = Higher, V = Highest. AUC = area under the curve. ∆ AUC: Percent change in AUC compared to Scenario 1 (S1). 95% uncertainty ranges are indicated in parentheses. (S1): distribution of deaths when no treatment is available; S2: current quintile-specific treatment coverage rates; S3: national average of treatment coverage for all quintiles; S4: treatment coverage proportional to quintile-specific baseline mortality risks; S5: equal baseline mortality risk with current quintile-specific treatment coverage rates

Each quintile faces different disease-specific mortality rates due to differences in the prevalence of prognostic factors and the probability of receiving treatment. When treatment is not available, deaths among measles cases would be concentrated in the lowest two quintiles (S1). When seeking treatment under current quintile-specific treatment coverage rates (S2), these unequal distributions remain because the proportion of people receiving treatment is much lower among the bottom quintiles; the differences in the treatment coverage rates between the highest and lowest quintiles were 28% in Nigeria, 23% in Pakistan, and 39% in Ethiopia (Fig. 5). S3 assumes equal treatment coverage for all quintiles, resulting in no distributional change (from S1) from the unequal distribution of the underlying mortality risks. If treatment coverage were proportional to underlying mortality risk (S4), we would expect to see small increases in the percentage of deaths averted by treatment and decreases in AUC in each country (though not significant given the large confidence interval around treatment effectiveness for measles (0.62, 95% CI 0.19–0.82 [19])). If, instead, the unequal distribution of underlying mortality risk was flattened and current quintile-specific treatment coverage was maintained, we would expect a more equal distribution of deaths (larger decreases in AUC) (S5).

### Pneumonia deaths

**Fig. 6:**
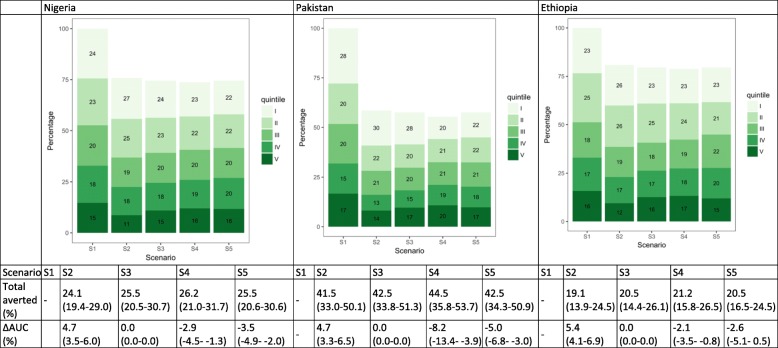
Distribution of pneumonia deaths by wealth quintile and scenario in Nigeria, Pakistan, and Ethiopia. The numbers in the green boxes represent the percentage of deaths in each wealth quintile. Wealth quintiles: I = Lowest, II = Lower, III = Middle, IV = Higher, V = Highest. AUC = area under the curve. ∆ AUC: Percent change in AUC compared to Scenario 1 (S1). 95% uncertainty ranges are indicated in parentheses. (S1): distribution of deaths when no treatment is available; S2: current quintile-specific treatment coverage rates; S3: national average of treatment coverage for all quintiles; S4: treatment coverage proportional to quintile-specific baseline mortality risks; S5: equal baseline mortality risk with current quintile-specific treatment coverage rates

Under S1, the lowest two wealth quintiles would have experienced close to half of deaths in Nigeria, Pakistan, and Ethiopia (Fig. 6). Under current treatment coverage patterns, 51.4% (50.2–52.6), 51.9% (50.0–53.9), and 52.3% (49.9–54.2) of pneumonia deaths would respectively occur among the lowest quintiles in each country (S2). When applying equal treatment utilization across quintiles (S3), we would expect the distribution of these deaths to remain the same as S1. If, instead, we increased treatment coverage among the bottom quintiles by setting treatment coverage proportional to baseline mortality risk, we would expect a more equal distribution of deaths (decreases in AUC) (S4) in most countries. This is especially visible in Pakistan, since the baseline mortality risk is estimated to be much higher among the lowest quintiles. Equalizing the baseline mortality risk first and then applying current quintile-specific treatment coverage would also lead to a more equal distribution of deaths. Similar to measles deaths, if we lower and equalize the underlying mortality risk, we would expect more equal distribution of deaths (larger decreases in AUC) (S5).

### Diarrhea deaths

**Fig. 7:**
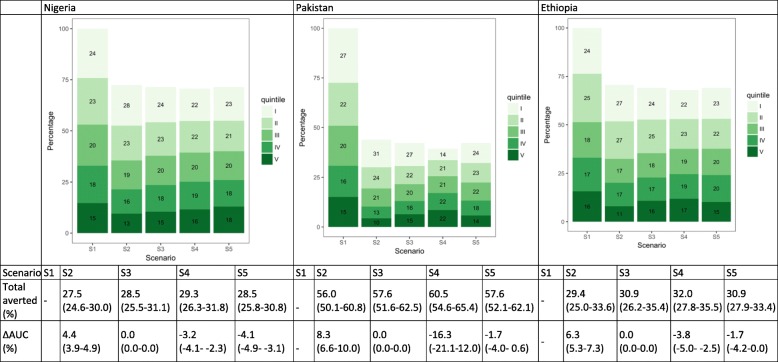
Distribution of diarrhea deaths by wealth quintile and scenario in Nigeria, Pakistan, and Ethiopia. The numbers in the green boxes represent the percentage of deaths in each wealth quintile. Wealth quintiles: I = Poorest, II = Poorer, III = Middle, IV = Richer, V = Richest. AUC = area under the curve. ∆ AUC: Percent change in AUC compared to Scenario 1 (S1). 95% uncertainty ranges are indicated in parentheses. (S1): distribution of deaths when no treatment is available; S2: current quintile-specific treatment coverage rates; S3: national average of treatment coverage for all quintiles; S4: treatment coverage proportional to quintile-specific baseline mortality risks; S5: equal baseline mortality risk with current quintile-specific treatment coverage rates

Under S1, 47.0% (46.2–47.7), 49.1% (47.7–50.7), and 48.6% (46.6–50.4) of deaths would occur in the lowest two quintiles in Nigeria, Pakistan, and Ethiopia, respectively (Fig. 7). Current quintile-specific treatment coverage (S2) increases AUC in all three countries due to higher treatment coverage rates among the higher wealth quintiles. If treatment coverage were proportional to underlying mortality risk, we would see improvements in the distribution of deaths for all three countries (decreases in AUC) (S4). This strategy would be more favorable than others in countries with less equal distribution of the underlying mortality risk such as Pakistan.

### Sensitivity analysis

The results of the two sets of sensitivity analyses are presented in Additional file 1: Section VI. With the TFR adjustment, higher TFR in lower wealth quintiles would lead to a greater number of susceptible children and therefore larger proportions of cases and deaths in these quintiles under S1. Therefore, the estimated effect of vaccines and treatments would be intensified because of the greater unequal distribution of underlying morbidity and mortality risks. When we replaced the equal distribution of unattributable cases and deaths with the ratio of under-five mortality rates to the national average, we found that most of the results were not different from the main findings, and that the changes were more favorable (more equal distribution of cases and deaths across quintiles).

## Discussion

Equity is increasingly gaining attention on the global development agenda [2, 4]. To understand whether certain health interventions can lead to changes in health equity, we developed an analytical approach to estimate the changes in the distribution of childhood disease-related incidence and mortality by wealth quintile with selected interventions. In particular, we applied this approach to examine how vaccine programs might affect health equity under different scenarios of intervention coverage and treatment utilization assumptions in LMICs.

Our study highlighted several factors that can substantially contribute to the unequal distribution of childhood disease. First, higher prevalence of risk factors, such as childhood malnutrition indicators, among the poor contribute to unequal distribution of childhood disease incidence and mortality, before any intervention (immunization or treatment) is even introduced. Second, large differences were observed in vaccine coverage across quintiles. In many countries, vaccine coverage among the top quintiles can be three-to-four times higher than among the bottom quintiles. Third, an unequal distribution of deaths was caused by the combination of unequal distribution of prognostic factors (thus an unequal mortality risk) and treatment coverage rates across quintiles. Our results suggest that the most appropriate strategy to remedy childhood disease inequities for each country would depend on country context, namely on how the baseline risks and current vaccine and treatment coverage are distributed across population subgroups.

The Global Vaccine Action Plan listed equity as a major principle in delivering universal access to immunization [7]; it emphasized “*equitable access to immunization*” as a core component of the right to health, and suggested that closing the coverage gap between the lowest and highest wealth quintiles would lead to greater equity. However, as shown in this study, merely ensuring equal access to vaccines will not reduce the health outcomes gap across wealth quintiles (see Scenario 3 in Figs. 2, 3, and 4). The poor face higher baseline risks, which are tied to social determinants of health such as wealth and education, in addition to lower treatment coverage rates. In our study, we find that Scenario 4 (distributing vaccines proportional to baseline risk) would often yield the most equitable distribution of disease burden, and that unequal distribution of vaccines that benefit the lowest wealth groups more would be required to achieve health equity.

This study makes an important contribution to the limited publications on the assessment of the distributional burden of childhood diseases and of the distributional impact of vaccines [20–22]. It builds on previous work, extends the analysis to three childhood diseases and their corresponding vaccines, and is replicable to a large number of countries. Our methodology could, in fact, be extended in the future to examine the distribution of other diseases and the distributional impact of other health interventions, and across other population subgroups (e.g. region, sex).

One potential application of this study is to provide inputs to decision-makers on how to determine appropriate equity-enhancing strategies for countries. We presented estimates for several policy options, including providing equal vaccine coverage across quintiles, targeting the poor, and addressing the underlying risks before improving vaccine coverage. One key input required to determine strategies is costs. We do not have data on how much more it would cost to increase intervention coverage across different socioeconomic groups, nor do we know how much effort it would take to address the inequities in the baseline morbidity and mortality risks. Thus, while we can conclude from our study that certain scenarios would be more effective than others, data on costs would be required to determine which scenario would be most cost-effective and sustainable under countries’ budget constraints.

This study has several limitations. First, a key limitation lies in our simplification of disease progression. We assumed that whether one gets a disease solely depended on the prevalence and relative risk of risk factors and vaccine coverage. As described above, this paper does not involve dynamic transmission models, and therefore does not reflect the potential nonlinear effects of vaccines on disease transmissibility and herd immunity [11, 12]. We were only able to identify contact matrices between different age groups [23], but not between wealth quintiles. On a related note, we searched for risk factors related to crowding, household, or neighborhood density as an attempt to account for the size of susceptible populations. Households in the lower wealth quintiles are more likely to have more children, and therefore disease transmission rates in these quintiles may be higher than the higher wealth quintiles with fewer children. However, we were unable to find the relative risks of related risk factors and/or the prevalence of these risk factors by wealth quintile in DHS. Furthermore, non-specific effects of vaccines, such as their effect on overall mortality [24], as well as the timeliness of receiving the vaccines [25, 26], were not taken into account. We believe that including these factors would lead to a more skewed distribution of cases. Second, two other important modes of delivery for measles vaccination, measles second dose and supplementary immunization activities, were not included in the analysis due to lack of coverage data by quintile. Third, for some countries, the DHS did not have complete data on the prevalence of risk and prognostic factors by wealth quintile, so we assumed they were at the same levels as neighboring or similar countries. Fourth, when taking the PAF approach, it is important that the definition of exposure and the studied population sample match closely with the prevalence data. For simplicity, we selected input data sources for risk factors from highly cited systematic reviews [14, 27, 28], and they may not perfectly meet the definitions of the prevalence data from the DHS. Similarly, the results depend on the selected data sources such as inputs for vaccine efficacy and treatment effectiveness. Since, for simplicity, we assumed they were constant across quintiles, changing the data sources would not lead to large changes in the results. However, we acknowledge that there are other data sources that could be equally suitable for this study [29–31]. We also reflected the uncertainties around these data sources by estimating uncertainty ranges for our results. However, we did not incorporate uncertainty around estimates of vaccine coverage, treatment coverage, and risk factor prevalence, due to the lack of immediate availability of uncertainty ranges from the original data sources. Finally, we were not able to validate the accuracy of the estimates since empirical data, especially for the null counterfactual scenario, were not available. One alternative for verification would be to collect disease-specific morbidity and mortality data at the subnational or national levels and examine the relationship between their respective wealth and socioeconomic levels. Our work points to these important data collection needs in the future.

## Conclusions

Our findings contribute toward understanding how diseases and the benefits of health interventions might be distributed, specifically in relation to achieving Sustainable Development Goal 3 in ensuring essential health services are provided for all. Achieving equity in health outcomes will only occur in step-by-step processes, which is why this paper is important in illustrating the potential distributional results of different approaches. The outputs can provide decision-makers with information on the possible distributional impact of policies, and thereby help promote more equitable resource allocation, even when empirical data are unavailable. Furthermore, the pursuit of health equity requires more than ensuring equal access to one intervention, and rather a more systematic approach in addressing the health gaps between population subgroups.

## Additional file

Additional file 1: Country selection and Demographic and Health Survey year; Methods in detail; Selection of risk and prognostic factors; Input data; Full results; Sensitivity analyses. (PDF 1013 kb)

## Abbreviations

AUC: Area under the curve
DHS: Demographic and Health Survey
LMIC: Low- and middle-income countries
MCV: Measles containing vaccine
PAF: Population attributable fraction
PCV: Pneumococcal conjugate vaccine
RV: Rotavirus vaccine
TFR: Total fertility rate
