# Supplementary material for: Estimating the distribution of morbidity and mortality of childhood diarrhea, measles, and pneumonia by wealth group in low- and middle-income countries
Source: BMC Med. 2018 Jul 4;16:102. doi: 10.1186/s12916-018-1074-y (PMC6030776; doi:10.1186/s12916-018-1074-y)
Supplement: Supplementary file 1 — Country selection and Demographic and Health Survey year; Methods in detail; Selection of risk and prognostic factors; Input data; Full results; Sensitivity analyses. (PDF 1013 kb) [file 12916_2018_1074_MOESM1_ESM.pdf]

Estimating the distribution of morbidity and mortality of childhood diarrhea, measles, and pneumonia by wealth group in low- and middle-income countries

Angela Y. Chang, Carlos Riumallo-Herl, Joshua A. Salomon, Stephen C. Resch, Logan Brenzel, Stéphane Verguet

Web Appendix

Table of Contents

I. Country selection and Demographic and Health Survey year 2

II. Methods in detail 4

a. An example of the method to distribute cases/deaths across wealth quintiles............... 4

b. Calculation of the area under the curve (AUC) 5

III. Selection of risk and prognostic factors 6

IV. Input data for quintile-specific vaccine and treatment coverage rates, risk and prognostic factor prevalence, and total fertility rate for 41 low- and middle-income countries .. 7

V. Full results.. 19

VI. Sensitivity analyses 26

VII. References 37

I. Country selection and Demographic and Health Survey year

We selected 41 low- and middle-income countries with Demographic and Health Surveys available after 2010, listed in Table S1 (1).

Table S1. List of 41 countries and Demographic and Health Survey year

Country	Year of Demographic and Health Survey
Armenia	2010
Bangladesh	2011
Benin	2011-12
Burkina Faso	2010
Burundi	2010
Cambodia	2014
Cameroon	2011
Chad	2014-15
Comoros	2012
Democratic Republic of the Congo	2013-14
Congo	2011-12
Côte d'Ivoire	2011-12
Ethiopia	2011
The Gambia	2013
Ghana	2014
Guinea	2012
Haiti	2012
Honduras	2011-12
Indonesia	2012
Kenya	2014
Kyrgyz Republic	2012
Lesotho	2014
Liberia	2013
Malawi	2010
Mali	2012-13
Mozambique	2011
Nepal	2011
Niger	2012
Nigeria	2013
Pakistan	2012-13
Rwanda	2010
Senegal	2014
Sierra Leone	2013
Tajikistan	2012
Tanzania	2010

Timor-Leste	2009-10
Togo	2013-14
Uganda	2011
Yemen	2013
Zambia	2013-14
Zimbabwe	2010-11

II. Methods in detail

a. An example of the method to distribute cases/deaths across wealth quintiles

We estimate the distribution of cases when no vaccines are available based on two inputs: the relative risk of the risk factor (how important the risk factor is in increasing the likelihood of the disease) and the prevalence of the risk factor (how common it is in each quintile).

As an example, assume we have three risk factors, A, B, and C for measles, and the relative risk of each are 5.0, 3.0, and 2.0. Following the equation $w_j = \frac{RR_j}{\sum_{k=1}^{n} RR_k}$, the weight we assign for each risk factor would be:

$$w_a = \frac{5.0}{(5.0 + 3.0 + 2.0)} = 0.5$$
$$w_b = \frac{3.0}{(5.0 + 3.0 + 2.0)} = 0.3$$
$$w_c = \frac{2.0}{(5.0 + 3.0 + 2.0)} = 0.2$$

Second, assume we know from the DHS that the prevalence of risk factor A for each quintile is 90%, 80%, 70%, 60%, and 50%, for risk factor B is 50%, 40%, 30%, 20%, 10%, and for risk factor C is 5%, 4%, 3%, 2%, 1%.

Then, following equation (1) from the main text $Ac_i = \frac{\sum_{j=1}^{n} w_j P_{ij}}{\sum_{m=1}^{5} \sum_{j=1}^{n} w_j P_{mj}}$, the proportion of attributable cases occurring in the first quintile would be:

$$\frac{(\text{weight of A} * \text{prevalence of A in quintile 1} + \text{weight of B} * \text{prevalence of B in quintile 1} + \text{weight of C} * \text{prevalence of C in quintile 1})}{\text{sum of the products of weight and prevalence of all quintiles}}$$

With this example, it would be:

$$\frac{(0.5 * 90\% + 0.3 * 50\% + 0.2 * 5\%)}{(0.5 * (90\% + 80\% + 70\% + 60\% + 50\%)\ +\ 0.3 * (50\% + 40\% + 30\% + 20\% + 10\%)\ +\ 0.2 * (5\% + 4\% + 3\% + 2\% + 1\%)} = 27\%$$

We interpret this to say that, out of the 100% of cases that would happen in this country, we expect to have 27% of cases in quintile 1.

b. Calculation of the area under the curve (AUC)

To compare the distributions of cases (or deaths) in each of the scenarios, we explored the area under the curve (AUC) of the cumulative percentage of cases (or deaths) by quintile, and calculated the changes in the AUC for each scenario, compared to Scenario 1 when no vaccines (or treatment) are available. For example, an increase in the AUC after vaccine program suggests that, among the remaining cases not averted by the program, a higher percentage of cases would be among the lower wealth quintiles compared to prior vaccine program. As illustrated in Figure S3.1, if cases are distributed equally across quintiles (i.e., 20% in each quintile), the AUC equals 250 (=5*100/2). Having an AUC greater than 250 (the area outside of the triangle) suggests that higher percentages of cases are in the lower wealth quintiles. The illustration shows a decrease in AUC after vaccine introduction (decrease in the area). On the other hand, an increase in the AUC between the two scenarios suggests that, among the remaining cases not averted by the program, a higher percentage of cases is among the lower wealth quintiles compared to prior vaccine introduction.

Figure S3.1. Illustration of the area under the curve analysis.

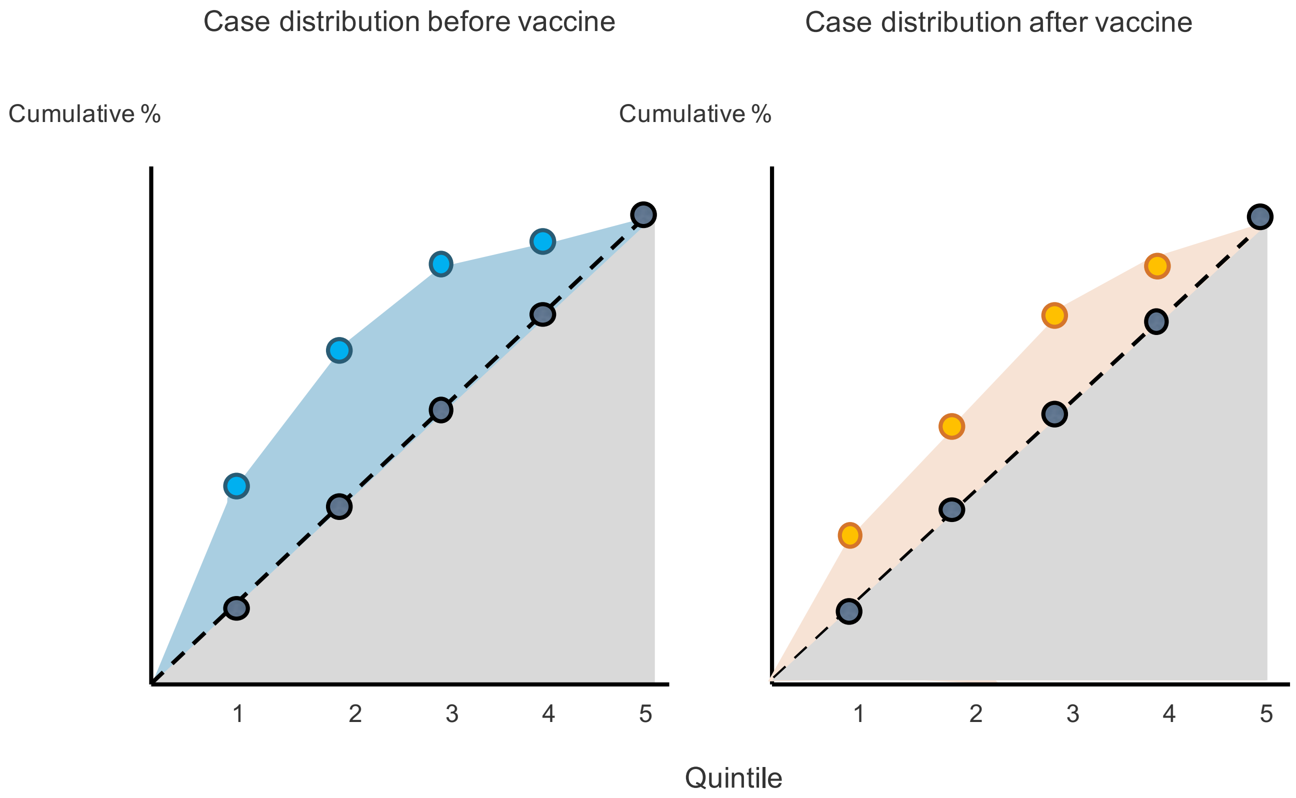

III. Selection of risk and prognostic factors

We first searched the literature for risk and prognostic factors with the highest relative risks for diarrhea, measles, and pneumonia. For each risk factor, we also checked its availability by quintile-specific prevalence rate in the DHS (1). Factors that did not match with DHS variables, had poor data quality, were not studied in systematic reviews, or had poor variable definition according to experts were excluded. We shortlisted five factors per disease (listed in the main paper, Table 2).

Prevalence rates by wealth quintile for malnutrition metrics (wasting, underweight, and stunting) and low birth weight were available in the DHS for most countries. The relative risk (RR) for each childhood malnutrition exposure is adjusted for the joint effect of the other malnutrition indicators, as described by the GBD 2013 Risk Factors Collaborators (2), and therefore assumed independent. We applied neighboring country data to countries with missing data.

Prevalence rates of vitamin A deficiency by wealth quintile were proxied with the percentage not given vitamin A supplements in the past six months among children aged 6 to 59 months. For countries missing this variable, we used the percentage who did not consumed foods rich in vitamin A in the past 24 hours among age 6 to 23 months as replacement.

For estimating the level of sanitation in one's household, we used the percentage of children whose stools were not disposed safely as a proxy.

For non-exclusive breastfeeding, the DHS provides data on the median duration (in months) of exclusive breastfeeding in the past three years by wealth quintile, and the national average (percentage) of exclusive breastfeeding among children of ages zero to five months and zero to six months. To estimate the burden of non-exclusive breastfeeding, we assumed that the ratio of the average duration of exclusive breastfeeding of a wealth quintile to the national average is proportional to the ratio of the percentage of people exclusively breastfeeding in the same wealth quintile to the national average. The burden of non-exclusive breastfeeding is calculated as 100% minus the estimated prevalence of exclusive breastfeeding.

IV. Input data

Table S4.1. Input data for quintile-specific vaccine and treatment coverage rates, risk and prognostic factor prevalence, and total fertility rate for 41 low- and middle-income countries.

Country	Quintile	MCV cover age	DPT2 cover age	DPT3 cover age	Among children under 5 with fever, percentage for whom advice or treatment was sought from a health facility or provider	Among children under 5 with acute respiratory infection, percentage for whom advice or treatment was sought from a health facility or provider	Percentage of children with diarrhoea for whom advice or treatment was sought from a health facility or provider	Weight-for-height (wasting) z-score < -3SD	Weight-for-age (underweight) z-score < -3SD	Height-per-age (stunting) z-score <-3SD
Armenia	1	94.6	91.3	88.3	NA	NA	NA	3.3	2	11
Armenia	2	99.1	100	99.1	NA	NA	NA	2	2	7.5
Armenia	3	96.8	94.2	94.2	NA	NA	NA	1.5	1.6	8.8
Armenia	4	94.1	96.1	96.1	NA	NA	NA	2.2	0.2	5.3
Armenia	5	91.8	99.1	96.9	NA	NA	NA	0.1	0.1	8.3
Bangladesh	1	79.2	93.1	90.3	22	25	20	4.5	16.6	24.5
Bangladesh	2	87.5	92.8	90.1	21	30	17	4.1	11.3	16.9
Bangladesh	3	88.1	96	93.2	26	29	22	3.9	11.5	14.1
Bangladesh	4	90.4	97.5	96.3	30	46	25	3.4	6.3	11.2
Bangladesh	5	93.6	99.3	97.8	43	58	49	3.7	3.9	6.4
Benin	1	53.7	67.7	58.7	28	20	27	10.8	23.1	33.6
Benin	2	62.7	75.9	67.6	37	29	36	6.4	17	27.6
Benin	3	71.9	84.1	76	38	45	40	9.6	19	29.3
Benin	4	76.7	88.3	80.2	39	31	45	5.2	15.9	32.3
Benin	5	84.7	91.6	85.6	47	31	37	7.1	9.3	20.4
Burkina Faso	1	78.1	88.3	83.4	42	36	36	6.7	11.1	19
Burkina Faso	2	86	91.6	97.9	46	43	41	5.7	8.3	17.5
Burkina Faso	3	89.3	93.6	91.6	57	66	47	6.1	7.4	16.5
Burkina Faso	4	91.7	95.8	92.5	59	56	57	5.1	6.2	11.5
Burkina Faso	5	92.5	95	92.9	68	70	56	4.8	3.7	5.4
Burundi	1	91.2	96.5	94.4	57	52	57	1.1	13.3	36.3
Burundi	2	94.1	98.2	95.2	64	53	58	1.1	8.4	28.6
Burundi	3	94.7	99.4	95.9	63	59	61	1.6	7.8	27.2
Burundi	4	95.8	98.8	96.9	62	53	51	1.4	6	24.8
Burundi	5	96.2	96.9	94.2	67	58	62	1.6	2.7	17.1
Cambodia	1	65.9	81.1	71.6	62	68	62	2.8	7.1	14.1
Cambodia	2	71.7	86.7	78.3	67	65	54	2.3	5.7	9.8
Cambodia	3	77.7	91	82.5	56	78	58	2.1	4.3	7.8
Cambodia	4	87.6	98	94.3	59	67	56	2.5	3.5	6.9
Cambodia	5	94.7	98.9	96.2	59	67	44	1.9	1.7	4.1
Cameroon	1	51.6	57.1	43.9	11	9	14	4	10.9	26.1
Cameroon	2	66	76.2	65.6	24	38	21	2.1	6.5	18
Cameroon	3	75.8	85.2	73.8	32	41	28	1.4	1.8	11.6
Cameroon	4	78.4	85.3	77.7	34	35	30	0.9	1.6	8.8
Cameroon	5	86.3	93	874	43	39	37	0.5	0.4	2.8
Chad	1	52.7	41.5	27.2	12.6	15.7	17.2	3.7	10.4	24.6
Chad	2	55.4	46.7	32.4	18.5	20.9	21.1	3.8	10.5	21.7
Chad	3	58.1	46.1	31.3	21.1	23.5	26.2	5.4	12.5	22.9
Chad	4	53.2	46.3	33.2	25	30.7	28.8	4.2	13.4	24.8

Chad	5	67.4	64.7	44.7	38.6	44.3	37.7	3.6	8.4	13.3
Comoros	1	62.5	67	57.5	40	NA	42	5.6	6.7	18.2
Comoros	2	70.1	75.6	65.9	39	NA	35	3.8	4.9	17.4
Comoros	3	83.2	84.9	80.5	43	NA	39	3.6	2.6	11.4
Comoros	4	83.6	88.2	82.5	52	NA	49	3.4	4.1	13.9
Comoros	5	79.8	84.6	81.7	50	NA	33	1.8	2.1	8.8
DRC	1	61.8	61	48	37	37	37	3	9.9	28.8
DRC	2	67.3	65.8	50.7	45	40	45	3.3	9.5	26.1
DRC	3	72.6	72.9	56.5	37	49	42	3	7.1	23.5
DRC	4	75.2	81.9	69.8	39	39	38	2.2	5.9	1
DRC	5	84.3	92.4	83	42	44	33	1.2	1.6	9
Congo	1	58.8	69.5	56.4	40	41	32	2	4	13.1
Congo	2	70.1	76.9	66.4	45	49	33	2.2	3.5	8.6
Congo	3	80.1	88.6	74.1	44	41	30	1.3	1.9	7.8
Congo	4	84.9	90.6	85.1	47	66	37	1	0.9	5.6
Congo	5	87.1	94.1	83.8	61	71	53	0.9	0.7	2.1
Cote d'Ivoire	1	53.9	62.5	52.1	22	18	19	1.6	6	19.4
Cote d'Ivoire	2	56.8	67.5	60.4	34	30	34	2	2.6	13.1
Cote d'Ivoire	3	65	68	58.1	34	50	23	1.5	3	10.9
Cote d'Ivoire	4	69	79.9	73.9	41	48	32	1.6	3	8.1
Cote d'Ivoire	5	83.1	84.3	80.7	42	42	29	1.8	1	4.9
Ethiopia	1	45.3	42.4	26	16	16	22	3.3	12	25.5
Ethiopia	2	52	45.2	29.4	21	25	25	4.1	11.1	23.1
Ethiopia	3	52	47.1	31	23	22	35	2.5	8.7	21.8
Ethiopia	4	56.4	56.6	42.2	28	33	33	2.1	5.9	17.4
Ethiopia	5	79.7	78.3	61.5	40	62	53	1.6	4.9	12
Gambia	1	93	95.5	90	66	70	67	3.4	4.4	10.7
Gambia	2	89.4	95.8	88.1	60	70	71	4.3	4.6	9.4
Gambia	3	89.3	94.8	88.3	63	71	67	5.8	4	8.8
Gambia	4	89.4	92.9	88.5	67	62	66	4.1	3.9	7.4
Gambia	5	76.2	97.7	82.7	70	67	66	3.5	2.1	3.7
Ghana	1	86.5	95.1	87.4	55	NA	54	1.1	2.7	7.6
Ghana	2	89.5	94.4	86.5	53	NA	47	0.9	1.9	8.2
Ghana	3	89.3	94.7	88.6	62	NA	34	0.5	1	4.2
Ghana	4	88.9	94.9	88.8	57	NA	41	0.3	1.3	2.2
Ghana	5	92.9	98.5	91.9	54	NA	46	0.4	1.3	1.3
Guinea	1	44.1	48.5	32.4	21	14.5	27	5.4	7.1	15.3
Guinea	2	64.6	61.8	52.7	27	30.6	30	3.2	7.4	19.7
Guinea	3	59.6	61.7	51.6	31	34	38	4.3	5.7	14
Guinea	4	66.4	67	54.5	43	48	43	2.9	3.1	10.7
Guinea	5	80.2	78.2	62.2	60	77.7	55	1.6	0.6	5.1
Haiti	1	65	69.4	54.7	28	22.9	28	1	4.3	12.9
Haiti	2	64.2	76.1	62.7	32	31.8	27	1.4	3.2	9.3
Haiti	3	69.2	78.8	66.9	44	36	34	1.1	3	5.9
Haiti	4	62.9	81.5	62.5	49	52.1	39	1.9	2.8	5.1
Haiti	5	62.8	90.2	67.9	54	51.2	49	0.4	1.3	2.1
Honduras	1	90	97.7	95.8	60	61.1	52	0.4	2.6	14.5
Honduras	2	87.6	97.1	94.7	56	60.2	53	0.2	1.1	5.6
Honduras	3	86.2	95.4	92.7	62	67	49	0.5	0.6	3.1
Honduras	4	84.9	97.2	95.7	69	65.3	54	0.1	0.3	1.7
Honduras	5	89.8	99.3	98.1	64	72.6	52	0.2	0.4	1

Indonesia	1	80	87	79.9	65.6	60.7	62.8	2.8	7.1	14.1
Indonesia	2	83.9	93.9	86.8	73.9	83.7	65.9	2.3	5.7	9.8
Indonesia	3	93.2	97.2	95.5	73.5	80.3	67.3	2.1	4.3	7.8
Indonesia	4	86.2	95.7	91.3	77.1	86.8	65	2.5	3.5	6.9
Indonesia	5	90.4	96.3	95.2	79.1	71	61.5	1.9	1.7	4.1
Kenya	1	76.4	91.7	83.3	60	63	61	1.6	4.2	12.3
Kenya	2	86.9	97.7	90.1	63	67	58	0.8	2.7	9.5
Kenya	3	88.3	97.2	92	60	65	56	0.8	1.9	7.4
Kenya	4	94.5	98.7	93.7	63	63	55	0.6	1.1	6.2
Kenya	5	93.1	95.2	92.7	69	74	56	0.6	0.8	3.3
Kyrgyz Republic	1	99.1	98	92.2	NA	NA	NA	1.6	1.6	7.7
Kyrgyz Republic	2	98	98	93	NA	NA	NA	0.9	0.6	4.7
Kyrgyz Republic	3	97.2	94.2	87.1	NA	NA	NA	1.3	1.2	6.2
Kyrgyz Republic	4	92.8	90.4	79.9	NA	NA	NA	1	0.6	4.8
Kyrgyz Republic	5	95.7	95.7	75	NA	NA	NA	0.8	0.4	5.3
Lesotho	1	87.6	95	83.7	61.5	59.9	58.9	1.2	3.6	15.5
Lesotho	2	85.3	94.3	80.9	59.2	66.5	51.9	0.9	2.6	15.8
Lesotho	3	97.1	96.1	93.5	57.1	63.4	43.1	0.3	0.2	9.8
Lesotho	4	91	93.6	88	70.1	63.4	45.2	0.1	1.2	9.1
Lesotho	5	89.7	96.5	79.7	53.2	63.4	54.6	0	0	2
Liberia	1	65	72.1	57.9	50.6	48.3	43.2	2.6	5.4	15.9
Liberia	2	74.1	81.5	71.7	57.2	56.4	46.8	2.1	5.7	16.3
Liberia	3	73	87.4	73.6	60.9	59.7	53.6	2	4.3	12.7
Liberia	4	78.1	87.9	78.3	61.2	44.3	44.2	1.6	2	7.7
Liberia	5	84.1	83.8	79.4	62.2	44	49.2	1.7	3.8	5.9
Malawi	1	90.2	94.9	91.3	60.8	61.8	63.8	2.8	4.5	24.1
Malawi	2	92.5	96.6	93.3	61	66.8	63.2	1.8	4.5	22.3
Malawi	3	91.5	95.6	92	68.3	76.8	60.5	1.9	3	19.8
Malawi	4	96	96.7	94.8	63.4	71.5	64.5	0.4	1.9	18
Malawi	5	95.9	96.7	94.3	71.6	75.2	58.2	0.5	1	13.5
Mali	1	61.8	63.6	47.6	18.4	18.4	19.1	6.3	13.5	26.4
Mali	2	68.6	70.4	57.6	28.4	25	31.9	6.2	12.8	21.5
Mali	3	68.9	72.2	61.4	23.6	30.2	25.8	6	10.1	21
Mali	4	76.8	80.3	71.5	33.8	45	39.4	3.3	5.1	17.5
Mali	5	82.9	86.1	77.6	67	53.1	41.3	3.6	2.5	8.1
Mozambique	1	74.8	76.6	64.9	47.6	44.1	50	3.7	7.5	25.7
Mozambique	2	74.9	81.7	70.2	42.7	39.4	47.9	2.1	4.4	24.5
Mozambique	3	79.3	89.1	79.2	59.5	NA	59.1	2.1	4.1	21.8
Mozambique	4	91	94.3	85.3	69.7	51.4	62.8	1.2	1.7	14.9
Mozambique	5	92.9	92.8	87.1	68	66.8	63.2	1.2	1.6	7.4
Nepal	1	86	91.8	87.6	29.8	NA	32.7	2.3	10.5	25.2
Nepal	2	85.2	93.5	89.7	38.2	NA	38.7	2.3	8.7	20
Nepal	3	85.2	94.4	90.5	44.7	NA	38.9	3	8.2	12.9
Nepal	4	92.2	96.7	96.7	50.1	NA	44.1	3.5	6.1	10.2
Nepal	5	96.1	99.5	98.4	46.4	NA	37.1	2	2	6.5
Niger	1	58.7	62.8	52.9	60.5	35.1	41.2	10.8	23.1	33.6

Niger	2	62.4	71.1	58.9	54.2	40.5	57.5	6.4	17	27.6
Niger	3	61.7	74.4	62.6	72.4	61.7	54.6	9.6	19	29.3
Niger	4	72.4	82.8	70.5	80.1	50.8	50.6	5.2	15.9	32.3
Niger	5	78.1	92.6	88.9	81.7	82.1	56.5	7.1	9.3	20.4
Nigeria	1	13.2	10.9	7	25	27	19	10.5	17.3	33.8
Nigeria	2	26.6	26.4	18.5	29	28	28	10.2	15.6	28.5
Nigeria	3	43.9	49.5	39.7	30	41	32	8.5	10.3	19.3
Nigeria	4	60.6	68.5	60	35	37	38	7.6	7.9	12.2
Nigeria	5	77.9	86.7	79.5	44	64	38	5.9	5.4	7.9
Pakistan	1	35.1	40.9	29.9	56	57	54	4.5	19.4	38.4
Pakistan	2	60.6	73.5	67.1	57	58	55	3.2	12	30.7
Pakistan	3	62.5	77.2	69.2	63	60	59	4	6	19.6
Pakistan	4	72.1	85	78.8	73	74	68	1.8	5.9	16.6
Pakistan	5	82.8	93.7	88	79	79	77	2.9	3	10
Rwanda	1	94	98	95.6	51	53	37.9	0.9	3.2	23.5
Rwanda	2	93	97.1	95.7	53	64	54	1	2.5	20.4
Rwanda	3	94.9	97.8	97.1	59	65	54.6	0.3	2.6	17.9
Rwanda	4	97	99.6	97.9	68	56	54.5	0.9	2	12.8
Rwanda	5	97.4	99.3	98.7	69	86	57.4	1	0.8	7.8
Senegal	1	78.2	91	85.6	44.2	48	30.8	0.7	4	8.5
Senegal	2	78.6	92.8	89.4	42.5	38.8	37.2	0.6	2.7	5.6
Senegal	3	79	91.2	86.7	43.7	45.05	30.4	0.9	1.8	4.3
Senegal	4	83.2	96.2	95.3	42.3	51.3	24.8	1	1.1	4
Senegal	5	83.5	99.2	91.6	56.4	28.4	42.2	0.5	0.4	1.6
Sierra Leone	1	81.4	89.6	78.6	65.2	67.7	63.8	3.6	6.2	22.5
Sierra Leone	2	77.5	86.4	77.3	66.6	71.9	55.6	5	6.9	21.1
Sierra Leone	3	76.4	87.8	79.1	70.2	75.3	74.1	4.2	5.3	17.7
Sierra Leone	4	78.4	92.7	80.5	67.4	70.1	66.6	3.5	5	16
Sierra Leone	5	79	86.7	72.3	56.4	75.1	68.2	3.3	3	10.2
Tajikistan	1	92.7	96.4	92.9	47.9	NA	48.1	5.2	4.5	12
Tajikistan	2	97.7	97.8	95.6	51.6	NA	45.8	3.4	4.8	10.1
Tajikistan	3	95	94.8	92.7	62.1	NA	65.9	3	2.3	7.3
Tajikistan	4	96	93.7	91.9	67.6	NA	58.5	4.3	3.9	9.9
Tajikistan	5	94.4	94.1	91.6	63.3	NA	53.1	3.4	2.7	9.2
Tanzania	1	79.9	90.6	84.1	58	NA	52	1.7	6.4	20.4
Tanzania	2	82.1	91	84.1	62	NA	54	0.9	4.8	19.5
Tanzania	3	79.8	91.6	87.3	61	NA	45	1.3	2.9	16.5
Tanzania	4	90.9	95.2	91.3	66	NA	53	0.9	2.2	13.7
Tanzania	5	93.8	97.7	96.9	81	NA	63	1.4	1.2	8.9
Timor-Leste	1	53.7	60.9	54.8	59	NA	66	8.8	18.9	37.3
Timor-Leste	2	63.7	66.2	62.5	69	NA	70	6.7	17.3	40.8
Timor-Leste	3	71	71.9	68.3	77	NA	78	7.6	17.3	36.1
Timor-Leste	4	78	79	75.8	73	NA	68	6.5	13.1	28.6
Timor-Leste	5	74.7	80.3	72.5	83	NA	78	5.1	9.6	20.2
Togo	1	74.8	89.2	84	36	41.7	35	1.8	5.1	12.7
Togo	2	67.6	84.5	77.7	32	51.8	23	1.3	5.2	13.6
Togo	3	67.5	84.9	78.6	34	39.9	23	1.7	3.5	13.5
Togo	4	78	92.8	83.1	44	47.8	33	1.9	2.6	6.2
Togo	5	83.4	95.1	90.9	56	69.1	40	0.5	1	1.9
Uganda	1	75.1	87.7	73.8	79	78	74	1.3	5.8	18.9
Uganda	2	72.1	88.2	71.6	79	79	73	2	3.2	12.5

Uganda	3	74.1	80.8	66	82	78	74	1	0.4	18.4
Uganda	4	76.4	83.6	70.6	78	77	73	2	2	11.7
Uganda	5	81.6	86	74.7	85	82	67	1	1.3	5.5
Yemen	1	51.8	57.1	42.4	28	31	29	7.7	23.6	33.2
Yemen	2	58.9	66.6	54.3	31	32	32	5.4	15.8	28.8
Yemen	3	63.7	68.8	57.9	33	35	33	3.9	12.4	24.7
Yemen	4	69.6	74	66.7	39	35	34	4.2	8.1	15.3
Yemen	5	77.1	89.1	83.7	39	42	40	4.2	4.8	8.1
Zambia	1	80.9	89	79.3	69	67	63	2.9	4.4	22.1
Zambia	2	80.6	91.6	83.4	73	66	67	1.7	3.5	17.2
Zambia	3	85.3	93	85.1	71	67	68	1.9	2.7	17
Zambia	4	90	98.1	92.4	75	75	67	2.5	2.8	16.2
Zambia	5	92.7	98	95.1	75	80	62	1.7	1.5	10.8
Zimbabwe	1	77.7	78.6	66.7	38	52	37	0.9	2.2	12.8
Zimbabwe	2	74.6	75.8	71.2	44	44	35	0.8	2.4	11.3
Zimbabwe	3	73.5	81.1	70.1	38	54	30	0.3	2.3	12.9
Zimbabwe	4	86.9	85	78.8	29	48	37	0.8	1.2	8.6
Zimbabwe	5	83.7	85.3	79.4	33	NA	40	0.3	0.6	6.1

Country	Quintile	Weight-for-height (wasting) z-score < -2SD	Weight-for-age (underweight) z-score < -2SD	Height-per-age (stunting) z-score <-2SD	Among children age 6-59 months, percentage given vitA supplements in past six months	Among age 6-23, percentage who consumed foods rich in vitA in the past 24 hours	Exclusive breastfeeding - median duration (months) breastfeeding in the past 3 years	Predominant breastfeeding - median duration (months) breastfeeding in the past 3 years	Low birth weight (<2500g), percent of births low weight	Percentage of children whose stools were disposed of safely	Total fertility rate
Armenia	1	7.8	7.9	26.2	NA	66.9	76.8 (percentage)	NA	8.9	73.0	1.8
Armenia	2	3.8	5.2	16.0	NA	67.1	82.7 (percentage)	NA	5.9	82.2	1.8
Armenia	3	0.5	5.5	19.3	NA	79.5	77.1 (percentage)	NA	6.8	91.2	1.6
Armenia	4	3.7	2.9	16.3	NA	80.4	68.7 (percentage)	NA	5.9	87.2	1.8
Armenia	5	1.2	1.5	18.6	NA	79.6	79.4 (percentage)	NA	6.1	87.3	1.5
Bangladesh	1	17.5	50.3	53.7	55.2	53.2	3.0	5.3	19.3	19.6	3.1
Bangladesh	2	16.2	41.6	45.4	56.5	60.6	3.2	4.4	19.3	24.1	2.5
Bangladesh	3	17.7	36.0	40.7	60.8	69.6	3.6	5.1	18.0	31.7	2.2
Bangladesh	4	13.6	27.5	35.9	64.1	68.3	4.2	5.0	15.6	49.8	2.1
Bangladesh	5	12.1	20.9	25.7	62.3	69.6	3.5	4.7	15.2	73.1	1.9
Benin	1	24.3	47.3	55.7	37.6	45.2	0.6	3.2	14.0	10.5	6.1
Benin	2	20.8	45.7	53.4	44.8	49.4	0.6	3.0	15.0	13.4	5.4
Benin	3	21.2	43.0	48.3	48.2	55.2	1.3	4.4	12.8	26.6	5.1
Benin	4	16.7	40.2	54.6	52.6	54.9	0.7	3.4	11.4	60.1	4.6
Benin	5	16.5	32.7	45.1	61.1	63.0	1.5	2.8	11.3	90.3	3.9
Burkina Faso	1	17.3	31.7	41.9	53.8	31.4	0.6	7.0	14.8	3.5	7.1
Burkina Faso	2	16.2	28.5	37.0	59.9	29.7	0.7	8.0	13.9	7.4	6.9
Burkina Faso	3	15.3	26.0	37.6	67.3	29.8	0.6	7.0	13.2	11.9	6.7
Burkina Faso	4	15.5	23.8	33.2	68.9	35.6	0.7	7.0	13.8	29.0	6.2
Burkina Faso	5	11.9	15.5	18.6	65.2	50.7	0.7	6.0	13.8	82.6	3.7
Burundi	1	7.2	40.7	70.0	80.9	84.9	3.5	6.0	14.4	77.6	6.2
Burundi	2	6.0	30.1	59.1	79.2	80.6	3.9	6.0	10.5	80.8	6.8
Burundi	3	5.2	29.5	59.8	78.9	84.6	4.5	6.0	11.2	80.0	6.5
Burundi	4	5.4	25.2	56.5	80.2	85.8	4.6	6.0	11.9	81.5	6.8
Burundi	5	5.4	17.0	41.4	85.1	81.5	3.2	5.0	6.8	85.0	5.7
Cambodia	1	11.0	31.0	41.9	68.9	82.0	4.3	5.0	10.8	60.2	3.8
Cambodia	2	11.4	27.5	37.1	68.9	86.5	4.4	5.0	8.2	64.3	2.8
Cambodia	3	8.4	23.3	31.7	69.9	86.8	3.7	5.0	7.3	75.3	2.8
Cambodia	4	9.3	22.0	29.1	73.6	82.7	4	5.0	7.6	79.8	2.4
Cambodia	5	7.4	13.0	18.5	67.5	85.7	1.2	3.0	5.6	76.9	2.2
Cameroon	1	10.7	29.8	48.6	60.6	63.1	0.5	6.0	8.2	54.1	7.0
Cameroon	2	6.9	19.9	42.0	55.9	69.9	0.5	5.0	7.3	66.5	6.4
Cameroon	3	4.8	9.4	31.0	55.4	77.8	0.5	4.0	7.5	78.6	5.6
Cameroon	4	2.6	6.5	22.8	52.0	80.1	0.7	3.0	7.2	85.0	4.2
Cameroon	5	2.1	3.9	12.3	51.2	77.6	0.6	3.0	8.5	92.6	3.3
Chad	1	11.6	28.9	41.2	44.1	42.3	3.5	5.0	6.7	13.6	7.0
Chad	2	12.6	27.3	39.8	45.4	42.0	3	5.4	6.5	16.0	7.0
Chad	3	14.3	31.1	40.4	41.5	44.8	2.8	5.2	4.8	19.4	6.8
Chad	4	13.4	33.2	44.7	39.7	44.3	2.6	4.8	6.5	22.9	6.2
Chad	5	13.0	22.3	31.5	50.7	56.5	1.9	2.8	8.2	68.7	5.3
Comoros	1	13.4	19.8	38.2	42.0	71.1	0.4	4.0	12.6	71.7	6.7
Comoros	2	10.9	18.8	32.5	48.1	63.9	0.4	2.0	16.6	77.3	4.6
Comoros	3	11.1	12.5	25.9	47.8	69.5	0.5	2.0	19.7	70.2	4.2
Comoros	4	9.1	12.9	27.0	51.0	78.3	0.6	2.0	15.3	72.2	3.5
Comoros	5	9.7	9.3	21.9	57.0	75.4	0.5	1.0	16.2	64.5	3.4
DRC	1	8.4	29.1	49.7	60.2	79.7	2.1	4.7	6.3	57.8	7.6
DRC	2	9.9	27.1	48.3	66.1	83.3	2.2	4.8	5.6	62.6	7.4
DRC	3	8.9	23.9	45.8	67.0	80.9	2.7	4.6	7.0	66.6	7.1

DRC	4	6.9	20.2	41.4	75.5	84.4	2.4	4.9	9.8	75.9	6.5
DRC	5	4.1	8.1	22.9	87.8	80.7	1.6	3.6	6.5	85.6	4.9
Congo	1	6.1	17.6	34.5	60.6	81.8	0.5	4.0	71.7	16.2	7.0
Congo	2	5.7	13.0	27.6	64.7	81.3	0.5	2.0	91.5	37.3	6.1
Congo	3	5.2	10.7	23.9	62.4	76.9	0.7	3.0	97.4	41.6	4.9
Congo	4	6.7	8.3	17.0	64.1	69.1	0.5	3.0	98.3	69.3	4.1
Congo	5	6.1	4.8	9.3	75.0	72.9	0.6	1.0	99.9	86.4	3.8
Cote d'Ivoire	1	10.0	20.7	38.4	50.3	56.3	0.5	6.0	15.0	16.2	6.7
Cote d'Ivoire	2	5.9	16	35.5	60.0	58.6	0.5	6.0	15.9	37.3	6.0
Cote d'Ivoire	3	6.9	12.8	27.7	53.8	58.7	0.4	6.0	12.9	41.6	5.5
Cote d'Ivoire	4	7.4	11.7	24.2	70.0	62.1	0.5	4.0	15.5	69.3	4.3
Cote d'Ivoire	5	6.9	10.0	15.5	76.7	65.5	0.4	3.0	11.9	86.4	3.2
Ethiopia	1	12.1	35.6	49.2	45.2	18.5	2.6	5.3	34.6	17.9	6.0
Ethiopia	2	12.3	33.2	47.7	53.2	20.4	2.3	5.2	29.1	25.2	5.7
Ethiopia	3	9.4	28.8	45.6	51.4	26.4	3.6	6.1	31.9	34.3	5.3
Ethiopia	4	7.7	25.8	45.0	59.6	28.1	2.3	5.2	25.6	46.1	5.0
Ethiopia	5	5.1	15.1	29.7	58.1	39.7	0.6	4.0	22.1	63.3	2.8
Gambia	1	11.0	21.8	29.5	69.4	45.9	2.7	6.4	14.0	86.1	6.7
Gambia	2	12.0	17.8	27.2	70.9	45.7	2.7	6.8	10.0	88.2	6.8
Gambia	3	13.6	17.9	25.2	69.5	47.3	1.7	6.0	9.6	86.5	6.2
Gambia	4	9.6	11.8	22.4	65.8	51.4	1.3	5.3	12.7	77.3	5.3
Gambia	5	11.0	9.7	15.2	67.4	48.8	2.2	4.9	12.1	68.6	3.8
Ghana	1	6.1	15.6	24.8	59.0	61.0	3.5	6.4	11.4	24.0	6.3
Ghana	2	3.8	13.3	25.5	71.2	65.6	3.0	5.4	8.6	39.2	5.5
Ghana	3	2.2	7.2	17.9	67.7	69.1	2.8	4.4	8.4	42.3	3.9
Ghana	4	6.8	11.6	14.4	61.9	71.9	2.6	4.3	9.9	44.1	3.5
Ghana	5	4.3	6.3	8.5	66.5	68.5	1.9	3.2	9.7	52.1	2.8
Guinea	1	13.3	19.8	33.8	33.0	16.9	0.7	7.7	8.5	38.7	6.5
Guinea	2	9.5	25.6	41.1	37.7	18.4	0.7	8.1	8.0	47.5	5.5
Guinea	3	8.8	18.6	33.8	38.2	27.2	0.7	6.9	6.4	61.6	5.7
Guinea	4	8.6	15.8	25.0	41.9	32.2	0.6	6.9	8.7	82.2	4.8
Guinea	5	6.4	4.8	15.4	57.9	45.6	0.6	4.4	9.9	92.8	3.4
Haiti	1	5.6	17.8	31.0	43.4	63.6	1.8	3.5	19.1	42.2	5.7
Haiti	2	4.5	11.4	26.5	45.3	60.9	1.8	2.2	21.7	57.1	4.7
Haiti	3	5.4	11.5	20.6	40.2	66.1	2.1	2.7	22.6	72.0	3.8
Haiti	4	6.3	7.9	16.1	46.0	68.0	1.1	1.4	17.7	84.4	3.1
Haiti	5	3.2	3.8	6.6	48.2	62.3	1.5	2.5	17.2	85.6	1.9
Honduras	1	1.4	10.2	27.6	74.4	77.9	2.4	4.6	13.6	31.2	4.6
Honduras	2	1.2	6.5	19.5	77.9	82.4	1.1	2.1	10.0	45.1	3.3
Honduras	3	0.9	4.7	13.3	72.3	82.0	0.6	0.6	8.2	49.4	2.9
Honduras	4	0.7	3.3	9.8	69.7	82.2	0.5	0.6	7.4	51.8	2.4
Honduras	5	1.1	2.7	7.0	71.5	87.2	0.5	0.6	8.4	50.0	2.0
Indonesia	1	11.0	31.0	41.9	53.0	79.8	1.1	2.7	11.2	43.1	3.2
Indonesia	2	11.4	27.5	37.1	58.3	81.5	0.7	1.1	7.2	58.4	2.7
Indonesia	3	8.4	23.3	31.7	63.6	81.8	1.9	2.3	7.8	71.9	2.5
Indonesia	4	9.3	22.0	29.1	66.4	83.9	0.6	0.7	6.3	76.4	2.4
Indonesia	5	7.4	13.0	18.5	65.1	86.9	0.7	0.7	5.0	74.3	2.2
Kenya	1	17.5	50.3	53.7	64.3	51.6	2.9	4.6	8.5	56.5	6.4
Kenya	2	16.2	41.6	45.4	71.3	70.8	3.2	4.5	5.7	88.5	4.7
Kenya	3	17.7	36.0	40.7	72.5	71.7	2.4	4.1	8.0	91.7	3.8
Kenya	4	13.6	27.5	35.9	76.2	84.7	4.1	4.7	8.8	91.9	3.1
Kenya	5	12.1	20.9	25.7	76.4	86.1	3.5	4.2	7.0	87.6	2.8
Kyrgyz Republic	1	2.7	4.5	18.3	NA	66.2	3.9	5.9	5.7	58	4.0

Kyrgyz Republic	2	2.1	2.6	18.6	NA	63.2	1.8	5.3	4.5	68.2	4.1
Kyrgyz Republic	3	3.2	3.7	16.7	NA	69.0	3.3	5.9	7.5	69.6	3.9
Kyrgyz Republic	4	2.0	2.9	16.4	NA	63.7	2.6	4.9	5.5	68	4.0
Kyrgyz Republic	5	4.1	3.3	19.0	NA	67.3	3.4	6.0	7.4	81.6	2.7
Lesotho	1	7.5	15.5	45.6	59.1	55.0	4.3	5.4	15.2	24.0	5.0
Lesotho	2	7.3	12.8	38.1	59.6	62.5	4.4	4.8	8.3	49.4	3.9
Lesotho	3	7.4	9.0	34.8	61.9	60.4	4.5	4.8	11.6	71.9	3.8
Lesotho	4	7.3	9.1	28.2	63.2	61.1	4.1	4.2	8.6	82.7	2.7
Lesotho	5	7.4	2.8	13.4	62.9	65.8	4.1	4.2	9.2	86.4	2.1
Liberia	1	6.1	18.3	35.3	50.8	68.1	3.2	7.3	10.8	15.9	6.6
Liberia	2	6.2	18.8	35.2	58.0	69.5	3.6	7.4	11.7	25.6	5.9
Liberia	3	6.4	15.1	35.3	61.2	66.1	3.1	6.4	7.7	30.4	5.2
Liberia	4	6.8	11.3	27.7	67.4	69.7	2.8	5.8	12.0	45.8	3.9
Liberia	5	3.9	8.5	19.9	69.2	55.9	1.7	3.6	7.0	47.3	2.8
Malawi	1	5.0	16.5	55.5	85.0	72.7	3.5	3.9	13.5	64.5	6.8
Malawi	2	4.5	14.3	50.8	84.9	72.7	3.4	4.0	13.2	76.2	6.8
Malawi	3	4.6	12.3	46.5	87.3	79.9	4.1	4.6	12.6	81.0	6.3
Malawi	4	3.8	13.7	46.8	86.1	79.7	4.1	4.4	11.8	82.1	5.3
Malawi	5	1.9	7.4	36.0	84.5	79.1	3.6	4.0	10.6	88.6	3.7
Mali	1	14.5	31.8	46.4	54.9	49.8	0.8	5.3	18.8	37.9	6.7
Mali	2	15.5	31.8	44.4	53.7	49.8	1.1	7.0	18.6	47.6	6.8
Mali	3	12.7	25.8	42.4	59.5	51.1	1.1	7.2	17.6	54.7	6.6
Mali	4	10.0	20.8	33.9	65.6	59.3	0.7	5.4	14.4	73.5	6.1
Mali	5	10.1	15.1	21.2	72.6	63.7	0.9	4.7	13.9	85.2	4.7
Mozambique	1	9.6	23.0	51.1	65.4	75.3	1.4	4.2	14.8	64.5	7.2
Mozambique	2	6.1	17.2	48.0	66.3	72.0	0.7	4.5	14.6	76.2	7.2
Mozambique	3	5.3	15.2	46.4	75.5	71.4	1.1	5.1	15.5	81.0	6.3
Mozambique	4	4.0	9.7	37.4	81.7	65.8	1.8	4.7	14.7	82.1	5.6
Mozambique	5	3.2	6.4	24.1	89.9	68.8	1.9	4.3	11.7	88.6	3.7
Nepal	1	12.5	40.3	56.0	89.4	47.4	4.6	5.3	16.8	19.6	4.1
Nepal	2	10.7	31.6	45.7	89.7	46.6	4.6	5.9	12.0	26.3	3.1
Nepal	3	12.8	28.8	34.5	91.4	39.9	4.7	6.0	12.1	33.8	2.7
Nepal	4	8.8	22.9	30.5	91.3	49.8	4.3	5.5	11.8	57.5	2.1
Nepal	5	7.4	10.0	25.8	90.8	52.7	2.2	3.4	12.2	84.7	1.5
Niger	1	24.3	47.3	55.7	52.7	NA	NA	NA	16.9	37.9	8.4
Niger	2	20.8	45.7	53.4	60.8	NA	NA	NA	19.5	47.6	8.3
Niger	3	21.2	43.0	48.3	60.7	NA	NA	NA	14.0	54.7	9.2
Niger	4	16.7	40.2	54.6	63.7	NA	NA	NA	9.0	73.5	8.9
Niger	5	16.5	32.7	45.1	87.6	NA	NA	NA	8.7	85.2	7.1
Nigeria	1	21.9	41.9	53.8	20.9	NA	0.4	4.9	13.5	56.7	7.0
Nigeria	2	19.7	34.8	46.1	30.9	NA	0.5	4.5	17.3	57.4	6.7
Nigeria	3	16.8	25.7	35.1	42.8	NA	0.5	3.9	9.1	51.7	5.7
Nigeria	4	16.7	22.1	26.3	53.0	NA	0.6	4	8.7	60.8	4.9
Nigeria	5	13.9	15.6	18.0	67.3	NA	1.1	4.4	6.8	72.3	3.9
Pakistan	1	17.3	47.8	61.6	62.9	32.6	0.7	4.4	67.1	19.6	5.2
Pakistan	2	10.5	34.2	55.7	76.7	39.5	0.7	2.6	42.7	24.1	4.4
Pakistan	3	9.4	26.1	40.6	77.3	44.6	1.2	2.9	40.0	31.7	3.8
Pakistan	4	7.8	22.4	37.8	74.6	49.5	0.7	1.4	22.8	49.8	3.4
Pakistan	5	8.2	15.6	23.0	69.7	60.6	1.0	1.3	19.1	73.1	2.7
Rwanda	1	3.5	15.5	54.0	91.5	66.9	5.1	5.8	9.0	82.4	5.4

Rwanda	2	3.2	13.8	51.1	91.7	70.8	5.3	6.0	6.9	84.4	5.2
Rwanda	3	2.0	11.4	45.7	92.3	72.5	5.9	6.7	5.8	87.2	4.5
Rwanda	4	2.4	9.2	39.2	95.2	74.0	5.2	5.9	5.5	89.4	4.4
Rwanda	5	2.8	5.2	25.8	94.6	83.0	4.7	5.3	4.0	91.6	3.4
Senegal	1	8.2	19.2	28.8	NA	NA	NA	6.4	NA	86.1	6.7
Senegal	2	6.7	14.7	21.7	NA	NA	NA	6.8	NA	88.2	6.8
Senegal	3	6.3	11.1	15.5	NA	NA	NA	6.0	NA	86.5	6.2
Senegal	4	3.4	8.6	13.4	NA	NA	NA	5.3	NA	77.3	5.3
Senegal	5	3.3	5.3	8.4	NA	NA	NA	4.9	NA	68.6	3.8
Sierra Leone	1	7.7	17.3	42.6	85.4	42.0	0.7	5.5	7.2	59.0	6.1
Sierra Leone	2	10.6	19.6	40.4	80.6	42.4	0.9	5.5	3.9	73.0	5.8
Sierra Leone	3	9.7	16.6	38.1	81.3	47.9	0.9	5.4	5.0	75.9	5.5
Sierra Leone	4	9.6	15.5	35.0	83.4	46.4	0.8	4.8	6.9	85.0	4.7
Sierra Leone	5	8.5	10.3	28.1	86.1	52.4	0.5	2.9	12.9	86.9	3.0
Tajikistan	1	9.5	15.8	32.1	77.9	43.1	1.4	5.8	9.5	91.1	4.1
Tajikistan	2	10.9	13.7	29.0	82.8	50.2	2.2	6.0	7.8	89.3	4.1
Tajikistan	3	9.6	10.2	23.4	76.0	57.9	1.5	5.6	6.8	88.7	3.9
Tajikistan	4	10.5	11.5	24.9	76.5	53.3	0.7	5.2	6.4	88.8	3.5
Tajikistan	5	9.0	9.3	20.9	68.1	53.8	0.7	3.9	5.8	89.9	3.2
Tanzania	1	6.6	21.5	48.4	53.3	62.5	3.2	3.8	6.3	61.0	7.0
Tanzania	2	3.8	17.9	45.1	54.2	65.8	2.7	3.5	6.3	67.2	6.8
Tanzania	3	4.4	14.4	44.3	62.7	58.0	2.4	3.2	5.3	73.5	6.1
Tanzania	4	4.4	12.8	39.2	68.7	58.5	2.1	2.8	6.7	80.7	4.7
Tanzania	5	4.7	9.3	26.3	69.5	62.5	1.6	2.2	9.1	86.7	3.2
Timor-Leste	1	20.8	49.4	63.0	43.8	74.0	3.1	3.6	10.6	13.8	7.3
Timor-Leste	2	18.7	48.0	63.5	44.4	73.5	3.3	3.9	16.5	16.5	6.0
Timor-Leste	3	19.6	48.1	60.5	50.7	78.1	2.2	3.6	11.7	22.0	6.1
Timor-Leste	4	17.6	41.4	55.4	56.6	82.4	2.5	3.2	10.5	36.8	5.3
Timor-Leste	5	16.2	35.3	47.1	58.5	88.4	1.4	1.6	8.1	50.5	4.2
Togo	1	8.0	20.2	33.4	85.3	68.1	2.9	5.5	9.3	7.5	6.3
Togo	2	6.6	21.1	37.5	77.7	72.7	2.3	5.4	9.5	12.9	5.8
Togo	3	7.0	17.3	32.5	80.0	65.4	2.7	5.3	11.2	25.7	5.4
Togo	4	6.6	12.2	19.4	83.8	65.9	3.4	6.1	9.0	68.9	3.9
Togo	5	4.0	7.2	10.6	81.8	71.1	3.2	5.8	8.8	86.3	3.5
Uganda	1	4.1	18.1	37.3	62.1	63.7	3.5	4.6	10.5	68.1	7.9
Uganda	2	6.2	14.3	30.9	58.3	63.3	3.7	5.0	8.5	82.4	7.1
Uganda	3	5.7	17.3	45.0	50.8	58.1	3.5	4.4	9.3	83.7	6.9
Uganda	4	4.5	9.5	30.5	55.5	59.5	3.6	4.3	10.9	87.4	6.1
Uganda	5	2.8	8.4	20.8	56.4	60.6	2.6	3.6	11.2	91.0	4.0
Yemen	1	20.7	54.0	59.2	47.4	34.0	NA	20.8	29.4	17.3	6.1
Yemen	2	17.8	45.1	55.5	55.0	36.9	NA	18.3	42.5	32.4	5.3
Yemen	3	14.2	37.9	48.1	55.6	45.7	NA	17.2	26.2	42.1	4.5
Yemen	4	15.0	31.0	38.3	58.2	59.0	NA	18.1	27.1	47.7	3.8
Yemen	5	12.5	22.1	25.9	61.6	67.6	NA	17.7	20.1	46.7	2.9
Zambia	1	6.8	20.1	47.3	70.5	72.8	4.2	4.9	10.0	77.0	7.1
Zambia	2	5.9	15.7	41.7	77.7	75.3	4.1	5.1	7.6	77.6	7.0
Zambia	3	5.2	13.7	40.2	75.8	74.6	4.2	4.8	8.9	83.1	6.0
Zambia	4	6.5	12.7	37.6	78.8	75.6	4.3	4.8	10.3	91.7	4.2
Zambia	5	5.7	8.7	28.4	82.6	77.9	3.5	4.2	9.4	91.5	3.0
Zimbabwe	1	3.2	12.0	36.8	60.1	63.0	0.6	3.6	9.9	64.3	5.3
Zimbabwe	2	3.3	11.0	32.0	62.4	62.0	0.7	3.4	7.6	77.7	5.1
Zimbabwe	3	2.9	9.0	35.0	66.9	70.0	1.3	3.1	10.2	85.5	4.4
Zimbabwe	4	3.7	8.8	28.6	70.1	66.2	1.1	2.6	9.9	94.2	3.8

Zimbabwe	5	1.1	5.7	23.8	70.2	68.9	2.4	3.3	9.5	96.3	2.6

NA: not available; MCV: measles containing vaccine; DPT: diphtheria, pertussis, and tetanus; SD: standard deviation; vitA: vitamin A.
Data source: Demographic and Health Survey (DHS) (1).

Table S4.2. Input data for the proportion of attributable and unattributable cases and deaths for 41 low- and middle-income countries.

	Measles		Pneumonia		Diarrhea	
Country	Proportion of cases attributable to risk factors	Proportion of deaths attributable to prognostic factors	Proportion of cases attributable to risk factors	Proportion of deaths attributable to prognostic factors	Proportion of cases attributable to risk factors	Proportion of deaths attributable to prognostic factors
Armenia	0.43	0.43	0.73	0.81	0.85	0.90
Burundi	0.78	0.78	0.93	0.94	0.96	0.97
Benin	0.84	0.84	0.94	0.96	0.97	0.98
Burkina Faso	0.82	0.82	0.95	0.96	0.98	0.98
Bangladesh	0.81	0.81	0.94	0.95	0.97	0.98
Cote d'Ivoire	0.78	0.77	0.92	0.94	0.95	0.97
Cameroon	0.69	0.69	0.90	0.92	0.94	0.96
Democratic Republic of the Congo	0.80	0.80	0.93	0.95	0.96	0.97
Congo	0.74	0.74	0.89	0.92	0.94	0.96
Comoros	0.82	0.82	0.94	0.96	0.97	0.98
Ethiopia	0.80	0.81	0.94	0.95	0.97	0.98
Ghana	0.69	0.69	0.88	0.90	0.93	0.95
Guinea	0.77	0.77	0.94	0.95	0.96	0.97
The Gambia	0.76	0.76	0.93	0.94	0.95	0.96
Honduras	0.26	0.25	0.77	0.82	0.82	0.88
Haiti	0.40	0.40	0.89	0.92	0.94	0.97
Indonesia	0.72	0.71	0.86	0.91	0.93	0.95
Kenya	0.72	0.75	0.87	0.90	0.92	0.95
Kyrgyzstan	0.53	0.53	0.76	0.82	0.90	0.93
Cambodia	0.76	0.76	0.91	0.94	0.95	0.97
Liberia	0.75	0.75	0.92	0.94	0.95	0.96
Lesotho	0.73	0.73	0.86	0.89	0.93	0.96
Mali	0.82	0.82	0.95	0.96	0.97	0.98

Mozambique	0.76	0.76	0.92	0.94	0.95	0.97
Malawi	0.74	0.74	0.91	0.92	0.95	0.96
Niger	0.88	0.88	0.96	0.97	0.98	0.99
Nigeria	0.78	0.78	0.93	0.95	0.97	0.98
Nepal	0.80	0.80	0.92	0.94	0.95	0.97
Pakistan	0.79	0.79	0.93	0.95	0.95	0.97
Rwanda	0.56	0.56	0.88	0.90	0.92	0.93
Senegal	0.70	0.70	0.91	0.92	0.94	0.95
Sierra Leone	0.78	0.78	0.94	0.95	0.96	0.98
Chad	0.86	0.86	0.96	0.97	0.98	0.98
Togo	0.72	0.72	0.91	0.93	0.95	0.96
Tajikistan	0.68	0.42	0.87	0.90	0.95	0.97
Timor-leste	0.84	0.84	0.94	0.96	0.97	0.98
Tanzania	0.68	0.68	0.91	0.93	0.95	0.96
Uganda	0.68	0.68	0.90	0.93	0.94	0.96
Yemen	0.89	0.89	0.93	0.95	0.97	0.99
Zambia	0.75	0.75	0.91	0.92	0.94	0.96
Zimbabwe	0.67	0.67	0.87	0.90	0.90	0.94

V. Full results

Table S5.1-S5.6 show the full results from the analysis for all 41 countries. For each scenario, we report the total number of cases/deaths averted (expressed as a percentage decrease) from Scenario 1, and the change in the area under the curve (expressed as a percentage) from Scenario 1.

The definitions of the five scenarios for the cases and deaths are:

Cases

S1: distribution of cases when no vaccine is available;
S2: current quintile-specific vaccine coverage rates;
S3: national average of vaccine coverage for all quintiles;
S4: vaccine coverage proportional to quintile-specific baseline mortality risks;
S5: equal baseline morbidity risk with current quintile-specific vaccine coverage rates.

Deaths

S1: distribution of deaths when no treatment is available;
S2: current quintile-specific treatment coverage rates;
S3: national average of treatment coverage for all quintiles;
S4: treatment coverage proportional to quintile-specific baseline mortality risks;
S5: equal baseline mortality risk with current quintile-specific treatment coverage rates.

Table S5.1. Change in the distribution of measles cases by wealth quintile for four scenarios, compared to when no vaccines are available (Scenario 1).

	Total cases averted (%)				Change in AUC (compared to S1) (%)			
Country	S2	S3	S4	S5	S2 AUC	S3 AUC	S4 AUC	S5 AUC
Armenia	82.4 (80.3, 84.4)	78.4 (76.4, 80.4)	79.2 (76.8, 81.7)	82.3 (80.3, 84.3)	-3.0 (-3.4, -2.6)	17.7 (15.9, 19.9)	-14.9 (-17.6, -12.4)	-11.2 (-12.1, -10.1)
Burundi	80.6 (78.5, 82.6)	78.0 (76.0, 80.0)	79.3 (77.1, 81.5)	80.8 (78.8, 82.7)	4.0 (3.5, 4.5)	14.4 (12.9, 16.1)	-15.5 (-18.5, -12.2)	-2.7 (-4.3, -1.0)
Benin	54.2 (52.2, 56.2)	54.2 (52.3, 56.2)	56.3 (54.9, 57.7)	55.3 (53.9, 56.6)	9.9 (9.3, 10.4)	7.7 (7.3, 8.1)	-8.2 (-9.2, -7.2)	3.7 (3.0, 4.3)
Burkina Faso	76.0 (74.1, 77.9)	74.3 (72.5, 76.2)	76.5 (74.6, 78.4)	76.5 (74.7, 78.3)	9.6 (8.7, 10.6)	14.7 (13.4, 16.2)	-17.3 (-19.7, -15)	2.8 (1.5, 4.1)
Bangladesh	76.7 (74.8, 78.7)	74.4 (72.5, 76.4)	76.8 (74.8, 78.8)	77.4 (75.5, 79.2)	9.0 (8.1, 10.0)	16.6 (15.2, 18.4)	-18.8 (-21.4, -16.1)	1.3 (-0.3, 3.0)
Cote d'Ivoire	54.0 (51.9, 56.0)	54.0 (51.8, 56.0)	56.7 (55.1, 58.3)	55.3 (53.9, 56.6)	9.1 (8.5, 9.7)	9.3 (8.7, 9.9)	-10.4 (-12.4, -8.4)	1.7 (0.0, 3.2)
Cameroon	66.4 (64.1, 68.7)	64.7 (62.4, 67.0)	70.1 (68.2, 72.0)	69.7 (68.0, 71.4)	16.9 (15.5, 18.3)	21.2 (19.5, 23)	-22.7 (-24.9, -20.4)	4.6 (2.0, 7.4)
DRC	65.4 (63.7, 67.2)	61.9 (60.3, 63.6)	71.6 (69.8, 73.5)	67.2 (65.6, 68.7)	8.5 (7.9, 9.1)	13.3 (12.5, 14.3)	-22.7 (-25.2, -20.4)	-1.5 (-2.6, -0.4)
Congo	68.1 (66.1, 70.1)	67.1 (65.0, 69.0)	71.5 (69.4, 73.6)	69.7 (68.0, 71.4)	15 (13.8, 16.3)	15.5 (14.2, 16.9)	-20.1 (-22.9, -17)	6.7 (4.6, 9.1)
Comoros	68.7 (66.7, 70.5)	67.8 (65.9, 69.6)	70.7 (69.0, 72.5)	69.7 (68.0, 71.4)	11.2 (10.3, 12.1)	15.9 (14.6, 17.2)	-18 (-19.9, -16.2)	2.2 (0.8, 3.8)
Ethiopia	59.1 (57.5, 60.7)	59.1 (57.5, 60.8)	63.2 (61.6, 64.8)	61.1 (59.7, 62.5)	12.1 (11.4, 12.8)	13.2 (12.4, 14.0)	-13.9 (-15.3, -12.3)	5.5 (4.4, 6.6)
Ghana	79.7 (77.6, 81.7)	76.6 (74.6, 78.6)	77.9 (75.4, 80.5)	79.9 (78.0, 81.8)	4.1 (3.6, 4.6)	15.6 (14.1, 17.4)	-13.5 (-17.1, -9.8)	-2.5 (-4.1, -1.1)
Guinea	44.3 (42.2, 46.4)	44.3 (42.2, 46.3)	47.9 (46.6, 49.2)	45.9 (44.8, 47.0)	6.3 (6.0, 6.7)	7.9 (7.5, 8.4)	-7.7 (-8.4, -6.9)	-2.9 (-3.7, -2.0)
Gambia	81.6 (79.6, 83.6)	80.0 (78.0, 82.1)	80.4 (78.1, 82.8)	81.2 (79.3, 83.2)	-10.7 (-12.3, -9.4)	4.0 (3.5, 4.5)	-9.8 (-14.7, -5.5)	-14.2 (-16, -12.6)
Honduras	76.6 (74.7, 78.4)	76.5 (74.7, 78.4)	76.6 (74.7, 78.5)	76.5 (74.7, 78.3)	-1.1 (-1.2, -1.0)	10.2 (9.2, 11.3)	-10.3 (-12.8, -7.9)	-4.2 (-5.1, -3.4)
Haiti	46.8 (45.2, 48.4)	46.8 (45.3, 48.3)	47.0 (45.9, 48.2)	46.8 (45.6, 47.9)	-0.5 (-0.6, -0.5)	2.9 (2.8, 3.1)	-2.8 (-3.6, -2.1)	-4.1 (-4.8, -3.5)
Indonesia	66.8 (65.1, 68.5)	66.7 (65.1, 68.4)	68.4 (66.7, 70.2)	67.2 (65.6, 68.7)	4.2 (3.9, 4.5)	13.6 (12.7, 14.7)	-14.4 (-16.7, -12.3)	-2.8 (-3.7, -2.0)
Kenya	68.1 (66.2, 69.9)	67.8 (65.9, 69.6)	70.3 (68.5, 72.1)	68.9 (67.2, 70.5)	7.7 (7.1, 8.3)	16.7 (15.5, 18.1)	-18.4 (-20.2, -16.5)	-0.8 (-2.1, 0.4)
Kyrgyz Republic	83.3 (81.2, 85.3)	82.7 (80.7, 84.8)	82.4 (80.2, 84.8)	83.2 (81.2, 85.2)	-4.5 (-5.2, -3.9)	7.2 (6.3, 8.3)	-2.9 (-6.5, 0.8)	-5.9 (-6.9, -4.9)
Cambodia	76.9 (75.0, 78.8)	75.3 (73.4, 77.1)	78.9 (77.0, 80.9)	77.7 (75.9, 79.6)	15.8 (14.3, 17.7)	15.0 (13.7, 16.6)	-15.9 (-18.3, -13.4)	9.6 (7.7, 11.7)
Liberia	50.4 (48.9, 52.0)	50.4 (48.9, 52.0)	52.0 (50.7, 53.3)	51.0 (49.8, 52.2)	4.2 (4.0, 4.4)	7.4 (7.1, 7.8)	-7.4 (-8.3, -6.6)	-3.0 (-3.6, -2.4)
Lesotho	79.4 (77.2, 81.6)	76.1 (73.9, 78.2)	77.4 (75.3, 79.6)	79.7 (77.8, 81.6)	3.6 (3.2, 4.0)	17.5 (15.7, 19.5)	-18.6 (-21.4, -15.7)	-5.6 (-8.0, -3.6)
Mali	68.3 (66.6, 70.1)	66.7 (65.0, 68.4)	72.6 (70.8, 74.5)	69.7 (68.0, 71.4)	10.1 (9.3, 11)	16.4 (15.2, 17.8)	-22.7 (-25, -20.2)	1.7 (0.5, 2.9)
Mozambique	72.1 (70.3, 74.0)	67.0 (65.2, 68.9)	73.9 (72.1, 75.8)	74.0 (72.2, 75.7)	10.3 (9.4, 11.2)	18.5 (17.1, 19.9)	-23.9 (-25.9, -22.0)	-1.1 (-2.2, 0.2)
Malawi	73.6 (71.5, 75.7)	71.2 (69.1, 73.3)	74.5 (72.4, 76.8)	74.0 (72.2, 75.7)	3.1 (2.8, 3.4)	17.6 (16, 19.3)	-20.8 (-23.5, -17.9)	-6.1 (-7.8, -4.0)
Niger	61.9 (59.9, 63.9)	61.9 (59.9, 63.8)	64.4 (62.6, 66.3)	62.9 (61.4, 64.4)	8.5 (8.0, 9.1)	11.9 (11.1, 12.7)	-12.8 (-14.2, -11.1)	2.0 (1.2, 2.7)
Nigeria	40.3 (38.5, 41.9)	40.3 (38.8, 41.9)	46.9 (45.7, 48.1)	45.1 (44.0, 46.1)	19.0 (18.0, 19.8)	6.8 (6.5, 7.1)	-8.2 (-9.3, -7.3)	11.5 (9.8, 12.9)
Nepal	75.9 (73.8, 78.0)	72.4 (70.2, 74.6)	76.9 (74.4, 79.4)	76.5 (74.7, 78.3)	6.1 (5.5, 6.8)	13.0 (11.9, 14.4)	-19.6 (-23, -16.2)	-1.3 (-3.8, 0.9)
Pakistan	51.9 (49.7, 54.0)	51.9 (49.7, 54.0)	58.7 (57.1, 60.4)	55.3 (54.0, 56.5)	14.6 (13.8, 15.4)	12.5 (11.9, 13.2)	-16.8 (-19.0, -14.8)	3.2 (1.6, 4.6)
Rwanda	83.1 (81.1, 85.1)	79.6 (77.6, 81.5)	80.1 (77.8, 82.6)	83.3 (81.3, 85.2)	3.9 (3.4, 4.6)	14.1 (12.5, 15.9)	-14.1 (-17.4, -10.9)	-2.3 (-3.4, -1.1)
Senegal	69.4 (67.5, 71.4)	68.9 (66.8, 70.9)	71.0 (69.0, 73.0)	69.7 (68.0, 71.4)	3.0 (2.8, 3.3)	15.1 (13.9, 16.3)	-17.3 (-20.1, -13.7)	-4.4 (-6.1, -2.8)
Sierra Leone	67.9 (66.2, 69.7)	67.9 (66.2, 69.7)	68.8 (67.0, 70.6)	68.0 (66.4, 69.6)	-0.8 (-0.9, -0.7)	8.6 (7.9, 9.3)	-8.3 (-10.3, -6.5)	-5.0 (-5.8, -4.2)
Chad	47.4 (45.9, 48.9)	47.4 (45.9, 48.9)	48.0 (46.8, 49.1)	47.6 (46.5, 48.7)	3.0 (2.8, 3.2)	0.7 (0.6, 0.7)	-0.4 (-1.1, 0.1)	2.6 (2.0, 3.1)
Togo	70.4 (68.5, 72.3)	67.4 (65.5, 69.2)	74.0 (71.9, 76.3)	71.4 (69.7, 73.1)	4.9 (4.4, 5.4)	11.0 (10.1, 12)	-19.6 (-22.5, -17.0)	-2.0 (-3.1, -1.0)
Tajikistan	83.2 (81.1, 85.2)	81.6 (79.6, 83.6)	81.7 (79.5, 84.0)	83.2 (81.2, 85.2)	1.2 (1.0, 1.4)	8.1 (7.1, 9.2)	-3.5 (-6.5, -0.3)	-1.3 (-1.9, -0.5)
Timor-Leste	63.8 (62.2, 65.5)	63.8 (62.2, 65.5)	65.5 (63.9, 67.2)	64.6 (63.1, 66.1)	11.2 (10.5, 12)	10.7 (10, 11.5)	-10.6 (-12, -9.3)	5.3 (4.4, 6.3)
Tanzania	81.4 (79.4, 83.4)	78.2 (76.3, 80.1)	80.5 (78.5, 82.7)	81.7 (79.8, 83.7)	6.4 (5.6, 7.3)	16.1 (14.4, 18.1)	-14.3 (-17.1, -11.4)	-0.3 (-1.4, 1.0)
Uganda	71.1 (69.1, 73.1)	70.8 (68.8, 72.8)	72.1 (69.8, 74.6)	71.4 (69.7, 73.1)	3.8 (3.5, 4.2)	9.0 (8.3, 9.8)	-9.4 (-12.3, -6.8)	0.2 (-0.9, 1.2)
Yemen	63.5 (61.7, 65.3)	63.0 (61.2, 64.9)	67.5 (65.8, 69.2)	65.5 (63.9, 67.0)	11.9 (11.1, 12.9)	18.3 (17, 19.7)	-20.5 (-22.3, -18.8)	1.6 (0.2, 3.0)
Zambia	73.6 (71.7, 75.4)	72.7 (70.9, 74.5)	73.9 (72.0, 75.9)	74.0 (72.2, 75.7)	7.9 (7.2, 8.7)	13.2 (12.1, 14.5)	-13.7 (-16.2, -11.5)	2.3 (1.2, 3.3)
Zimbabwe	79.0 (77.1, 81.0)	76.7 (74.8, 78.5)	79.2 (77.0, 81.4)	79.4 (77.5, 81.3)	7.2 (6.5, 8.1)	12.7 (11.5, 14.1)	-16.6 (-19.0, -14.4)	1.8 (0.8, 2.9)

Table S5.2. Change in the distribution of pneumonia cases by wealth quintile for four scenarios, compared to when no vaccines are available (Scenario 1).

	Total cases averted (%)				Change in AUC (compared to S1) (%)			
Country	S2	S3	S4	S5	S2 AUC	S3 AUC	S4 AUC	S5 AUC
Armenia	6.6 (0.0, 15.2)	5.9 (0.0, 14.5)	6.3 (0.0, 14.8)	16.1 (9.7, 22.3)	0.1 (0.0, 0.2)	0.3 (0.1, 0.6)	-0.3 (-1.2, 0.5)	-5.7 (-7.4, -3.9)
Burundi	6.8 (0.3, 13.4)	6.7 (0.2, 13.2)	6.7 (0.2, 12.8)	7.6 (3.7, 11.7)	0.0 (0.0, 0.0)	0.1 (0.0, 0.1)	0.0 (-1.0, 1.0)	-1.1 (-3.3, 0.5)
Benin	5.0 (0.0, 10.5)	5.0 (0.0, 10.5)	5.2 (0.0, 10.4)	9.1 (5.5, 12.4)	0.4 (0.1, 0.6)	0.4 (0.1, 0.6)	-0.4 (-1.1, 0.4)	-4.3 (-4.9, -3.6)
Burkina Faso	6.5 (2.1, 10.9)	6.4 (2.1, 10.7)	6.5 (2.2, 10.7)	7.8 (4.3, 11.5)	0.1 (0.0, 0.1)	0.2 (0.1, 0.4)	-0.2 (-1.1, 0.6)	-3.2 (-4.0, -2.4)
Bangladesh	6.6 (2.3, 11.0)	6.4 (2.3, 10.7)	6.5 (2.3, 10.7)	8.8 (5.7, 12.1)	0.1 (0.0, 0.2)	0.3 (0.1, 0.5)	-0.3 (-1.2, 0.6)	-4.0 (-4.9, -3.1)
Cote d'Ivoire	5.4 (0.7, 9.7)	5.4 (0.8, 9.7)	5.4 (1.0, 9.6)	5.4 (2.4, 8.6)	0.5 (0.2, 0.8)	0.1 (0.0, 0.2)	-0.1 (-0.9, 0.7)	-1.5 (-2.7, -0.5)
Cameroon	2.5 (0.0, 12.3)	2.5 (0.0, 12.4)	6.1 (0.0, 15.8)	11.0 (6.2, 16.7)	0.8 (0.3, 1.3)	0.3 (0.1, 0.5)	-0.5 (-1.3, 0.4)	-6.3 (-8.6, -4.1)
DRC	5.5 (0.0, 12.0)	5.4 (0.0, 12.0)	5.9 (0.0, 12.4)	9.6 (4.9, 14.4)	0.5 (0.2, 0.8)	0.4 (0.1, 0.6)	-0.4 (-1.1, 0.4)	-3.5 (-4.2, -2.7)
Congo	6.0 (0.3, 11.8)	6.0 (0.2, 11.8)	6.3 (0.3, 11.8)	6.9 (3.7, 10.7)	0.5 (0.2, 0.8)	0.2 (0.1, 0.3)	-0.2 (-1.1, 0.7)	-2.2 (-3.7, -0.5)
Comoros	NA	NA	NA	NA	NA	NA	NA	NA
Ethiopia	5.0 (0.0, 13.3)	5.0 (0.0, 13.4)	5.6 (0.0, 13.8)	9.2 (4.6, 14.9)	0.7 (0.3, 1.1)	0.3 (0.1, 0.5)	-0.3 (-1.2, 0.6)	-4.0 (-5.4, -0.7)
Ghana	6.8 (0.0, 15.4)	6.7 (0.0, 15.2)	6.7 (0.0, 15.0)	8.4 (4.0, 13.4)	0.1 (0.0, 0.1)	0.0 (-0.1, 0.0)	0.0 (-1.0, 1.1)	0.1 (-4.2, 3.4)
Guinea	NA	NA	NA	NA	NA	NA	NA	NA
Gambia	6.9 (1.4, 12.2)	6.7 (1.3, 11.9)	6.6 (1.4, 11.7)	8.1 (4.5, 11.5)	-0.1 (-0.1, 0.0)	0.0 (0.0, 0.0)	0.0 (-0.8, 0.9)	0.3 (-0.4, 1.1)
Honduras	6.1 (0.0, 12.2)	6.0 (0.0, 12.1)	6.2 (0.0, 11.9)	8.9 (3.5, 13.2)	0.0 (0.0, 0.1)	-0.3 (-0.4, -0.1)	0.3 (-0.6, 1.1)	2.8 (1.2, 3.7)
Haiti	2.0 (-3.0, 7.0)	2.0 (0.0, 7.0)	2.0 (0.0, 7.0)	5.2 (2.3, 8.5)	0.1 (0.0, 0.1)	0.0 (0.0, 0.0)	0.0 (-0.3, 0.2)	1.4 (0.7, 1.9)
Indonesia	NA	NA	NA	NA	NA	NA	NA	NA
Kenya	5.8 (0.8, 10.6)	5.8 (0.8, 10.6)	5.9 (1.0, 10.4)	10.4 (7.1, 13.7)	0.1 (0.0, 0.2)	0.4 (0.2, 0.7)	-0.4 (-1.2, 0.4)	-4.2 (-5.3, -3.1)
Kyrgyz Republic	5.5 (0.0, 12.4)	5.5 (0.0, 12.4)	5.5 (0.0, 12.2)	6.0 (2.6, 9.7)	-0.3 (-0.4, -0.1)	0.1 (0.0, 0.1)	-0.1 (-0.8, 0.7)	-1.2 (-2.5, -0.1)
Cambodia	6.5 (0.0, 14.8)	6.5 (0.0, 14.9)	6.7 (0.0, 14.7)	7.0 (3.8, 10.7)	0.3 (0.1, 0.5)	-0.1 (-0.1, 0.0)	0.0 (-1.0, 1.1)	0.9 (-3.0, 5.5)
Liberia	4.9 (0.0, 11.1)	4.9 (0.0, 11.1)	4.9 (0.0, 10.9)	5.3 (2.3, 8.9)	0.3 (0.1, 0.5)	0.1 (0.0, 0.1)	-0.1 (-0.8, 0.6)	-1.3 (-3.5, 1.5)
Lesotho	6.2 (0.0, 13.7)	6.2 (0.0, 13.6)	6.2 (0.0, 13.3)	7.5 (3.6, 12.5)	0.0 (0.0, 0.0)	0.3 (0.1, 0.5)	-0.3 (-1.2, 0.6)	-4.0 (-6.3, -1.6)
Mali	5.4 (0.0, 11.1)	5.4 (0.0, 11.1)	5.7 (0.0, 11.5)	10.0 (5.5, 14.1)	0.5 (0.2, 0.8)	0.4 (0.2, 0.7)	-0.4 (-1.1, 0.3)	-4.0 (-4.8, -3.1)
Mozambique	5.5 (0.0, 12.3)	5.3 (0.0, 12.2)	5.8 (0.0, 12.3)	14.1 (10.5, 17.9)	0.3 (0.1, 0.5)	0.6 (0.2, 0.9)	-0.6 (-1.3, 0.2)	-6.3 (-7.9, -4.8)
Malawi	6.5 (0.0, 16.4)	6.0 (0.0, 15.8)	6.3 (0.0, 16.0)	14.2 (7.2, 21.4)	0.0 (0.0, 0.1)	0.4 (0.2, 0.7)	-0.4 (-1.2, 0.4)	-6.3 (-7.6, -4.7)
Niger	4.8 (0.0, 10.1)	4.8 (0.0, 10.1)	5.0 (0.0, 10.1)	9.4 (6.0, 12.8)	0.5 (0.2, 0.8)	0.3 (0.1, 0.6)	-0.4 (-1.1, 0.4)	-4.0 (-4.8, -3.2)
Nigeria	4.0 (0.6, 7.3)	4.0 (0.8, 7.1)	4.9 (1.4, 8.3)	7.9 (6.0, 9.9)	1.3 (0.4, 2.1)	0.3 (0.1, 0.5)	-0.3 (-1.0, 0.4)	-3.7 (-4.2, -3.1)
Nepal	6.5 (0.0, 15.1)	6.5 (0.0, 15.1)	6.5 (0.0, 14.5)	7.1 (3.2, 11.7)	0.2 (0.1, 0.3)	0.0 (-0.1, 0.0)	0.0 (-0.9, 1.0)	0.6 (-2.0, 3.6)
Pakistan	5.0 (0.0, 11.0)	5.0 (0.0, 11.0)	5.4 (0.0, 11.4)	10.0 (5.8, 13.7)	0.9 (0.4, 1.4)	0.5 (0.2, 0.7)	-0.5 (-1.3, 0.4)	-5.7 (-6.7, -4.8)
Rwanda	6.9 (0.8, 12.9)	6.4 (0.5, 12.2)	6.7 (0.5, 12.6)	13 (8.3, 17.3)	0.0 (0.0, 0.1)	0.1 (0.0, 0.1)	0.0 (-1.1, 1.1)	-1.0 (-3.0, 0.7)
Senegal	6.4 (0.0, 13.0)	6.4 (0.0, 13.0)	6.4 (0.0, 12.5)	7.2 (3.1, 11.9)	0.1 (0.0, 0.2)	0.2 (0.1, 0.3)	-0.2 (-1.1, 0.7)	-2.2 (-4.2, -0.1)
Sierra Leone	6.3 (0.8, 11.6)	6.3 (0.8, 11.6)	6.3 (1.0, 11.4)	6.5 (3.1, 10.2)	-0.1 (-0.1, 0.0)	0.1 (0.0, 0.1)	-0.1 (-0.9, 0.8)	-1.0 (-1.9, 0.2)
Chad	NA	NA	NA	NA	NA	NA	NA	NA
Togo	6.2 (0.0, 12.5)	6.0 (-0.4, 12.2)	6.4 (0.0, 12.7)	10.7 (6.1, 15.5)	0.1 (0.0, 0.1)	0.2 (0.1, 0.4)	-0.4 (-1.1, 0.4)	-2.8 (-3.6, -2.0)
Tajikistan	NA	NA	NA	NA	NA	NA	NA	NA
Timor-Leste	NA	NA	NA	NA	NA	NA	NA	NA
Tanzania	6.7 (0.1, 13.0)	6.6 (0.1, 12.9)	6.7 (0.3, 12.7)	7.5 (3.7, 11.3)	0.1 (0.1, 0.2)	0.0 (0.0, 0.0)	0.0 (-1.0, 1.1)	0.1 (-2.6, 2.5)
Uganda	5.6 (0.0, 12.8)	5.6 (0.0, 12.8)	5.6 (0.0, 12.6)	6.4 (3.0, 10.8)	0.0 (0.0, 0.0)	0.0 (0.0, 0.0)	0.0 (-0.8, 0.7)	-0.2 (-1.0, 1.2)
Yemen	5.8 (0.4, 11.3)	5.8 (0.4, 11.2)	6.2 (0.9, 11.6)	8.6 (5.4, 11.9)	0.5 (0.2, 0.9)	0.4 (0.2, 0.7)	-0.3 (-1.3, 0.5)	-5.2 (-6.5, -3.8)
Zambia	6.2 (0.4, 11.8)	6.2 (0.4, 11.8)	6.2 (0.4, 11.8)	7.1 (3.8, 10.3)	0.3 (0.1, 0.4)	0.1 (0.1, 0.2)	-0.1 (-1.1, 0.8)	-2.0 (-3.2, -0.4)
Zimbabwe	6.5 (1.7, 11.4)	6.2 (1.5, 10.8)	6.5 (1.7, 11.3)	11.2 (7.6, 15.1)	0.2 (0.1, 0.4)	0.2 (0.1, 0.4)	-0.3 (-1.2, 0.5)	-3.2 (-3.8, -2.7)

N/A: estimates not available due to no vaccine introduction in 2016.

Table S5.3. Change in the distribution of diarrhea cases by wealth quintile for four scenarios, compared to when no vaccines are available (Scenario 1).

	Total cases averted (%)				Change in AUC (compared to S1) (%)			
Country	S2	S3	S4	S5	S2 AUC	S3 AUC	S4 AUC	S5 AUC
Armenia	13.3 (0.2, 26.3)	11.5 (0.0, 23.0)	13.1 (0.5, 26.5)	23.6 (13.4, 33.8)	0.1 (0.0, 0.3)	1.1 (0.1, 2.4)	-0.8 (-4.1, 1.9)	-8.8 (-10.6, -7.3)
Burundi	13.5 (1.7, 26.4)	13.5 (1.6, 26.3)	13.4 (1.8, 26.2)	13.7 (3.6, 26.1)	0.0 (0.0, 0.1)	0.6 (0.1, 1.3)	-0.5 (-3.7, 2.5)	-3.6 (-5.3, -2.1)
Benin	NA	NA	NA	NA	NA	NA	NA	NA
Burkina Faso	12.8 (1.8, 24.8)	12.3 (1.7, 24.0)	13.0 (2.1, 25.2)	15.8 (7.0, 26.2)	0.2 (0.0, 0.5)	0.7 (0.1, 1.5)	-0.9 (-3.9, 1.5)	-4.9 (-6.8, -3.3)
Bangladesh	NA	NA	NA	NA	NA	NA	NA	NA
Cote d'Ivoire	7.3 (0.0, 14.8)	7.3 (0.0, 14.8)	7.6 (0.4, 15.2)	10.5 (5.8, 16.2)	0.5 (0.1, 0.9)	0.6 (0.1, 1.3)	-0.7 (-2.6, 0.9)	-5.5 (-6.8, -4.2)
Cameroon	11.8 (0.9, 23.3)	10.3 (0.5, 20.7)	12.3 (0.8, 24.9)	24.6 (18.3, 32.2)	1.0 (0.1, 2.0)	1.5 (0.2, 3.2)	-1.5 (-4.4, 0.8)	-11 (-12.8, -9.3)
DRC	NA	NA	NA	NA	NA	NA	NA	NA
Congo	12.5 (1.1, 24.4)	12.0 (1, 23.4)	12.7 (1.3, 25.4)	17.0 (9.0, 26.5)	0.8 (0.1, 1.7)	1.1 (0.1, 2.4)	-1.2 (-4.1, 1.4)	-7.2 (-8.9, -5.3)
Comoros	NA	NA	NA	NA	NA	NA	NA	NA
Ethiopia	11.1 (1.3, 21.2)	10.9 (1.2, 20.9)	12.6 (1.3, 24.0)	16.7 (9.6, 23.1)	1.0 (0.1, 2.0)	1.2 (0.1, 2.5)	-1.5 (-4.0, 1.0)	-6.5 (-7.8, -4.9)
Ghana	13.7 (1.2, 26.8)	13.1 (1.1, 25.8)	13.4 (1.7, 26)	15.0 (6.2, 26.4)	0.1 (0.0, 0.2)	0.8 (0.1, 1.7)	-0.6 (-4, 2.4)	-5.8 (-7.4, -4.1)
Guinea	NA	NA	NA	NA	NA	NA	NA	NA
Gambia	13.5 (1.9, 26.2)	13.4 (1.9, 26.1)	13.2 (2.1, 25.5)	14.0 (4.5, 26.1)	0.0 (0.0, 0.0)	0.1 (0.0, 0.1)	0.0 (-2.9, 2.7)	0.2 (-0.4, 0.7)
Honduras	12.8 (1.6, 24.9)	12.8 (1.6, 24.9)	12.9 (1.8, 24.9)	13.1 (3.7, 24.7)	0.0 (0.0, 0.1)	0.6 (0.1, 1.2)	-0.6 (-3.8, 2.2)	-3.3 (-4.5, -2.0)
Haiti	10.6 (0.4, 21.1)	10.2 (0.3, 20.5)	11.4 (0.7, 22.6)	16.3 (8.6, 24.8)	0.5 (0.1, 1.0)	0.7 (0.1, 1.5)	-0.8 (-3.7, 1.4)	-3.6 (-5.5, -2.0)
Indonesia	NA	NA	NA	NA	NA	NA	NA	NA
Kenya	12 (1.1, 23.6)	11.8 (0.9, 23.2)	12.1 (1.3, 23.9)	15.8 (8.9, 23.8)	0.1 (0.0, 0.2)	1.2 (0.2, 2.6)	-1.1 (-4.0, 1.4)	-7.4 (-9.9, -5.0)
Kyrgyz Republic	NA	NA	NA	NA	NA	NA	NA	NA
Cambodia	NA	NA	NA	NA	NA	NA	NA	NA
Liberia	6.8 (0.6, 13.3)	6.8 (0.6, 13.3)	6.9 (0.6, 13.7)	9.5 (4.9, 14.4)	0.2 (0.0, 0.4)	0.5 (0.1, 1.0)	-0.5 (-2.1, 0.9)	-4.3 (-5.5, -3.1)
Lesotho	NA	NA	NA	NA	NA	NA	NA	NA
Mali	10.7 (1.5, 20.6)	10.6 (1.5, 20.5)	11.4 (1.6, 22.1)	16.8 (10.6, 23.4)	0.6 (0.1, 1.3)	1.3 (0.2, 2.6)	-1.2 (-3.8, 1.1)	-6.2 (-8.3, -4.4)
Mozambique	10.5 (1.2, 20.7)	10.2 (1.1, 20.0)	11.3 (1.3, 22.2)	19.3 (13.2, 25.7)	0.4 (0.1, 0.9)	1.5 (0.2, 3.2)	-1.5 (-4.4, 0.8)	-8.7 (-10.7, -7.1)
Malawi	13.2 (0.5, 26.3)	11.8 (0.3, 23.4)	13.0 (0.2, 26.1)	21.4 (11.9, 31.1)	0.0 (0.0, 0.1)	1.1 (0.1, 2.3)	-1.2 (-4.0, 1.2)	-8.6 (-11.3, -6.4)
Niger	10.8 (1.1, 21.2)	10.8 (1.1, 21.1)	11.4 (1.1, 22.5)	14.1 (7.9, 21.7)	0.9 (0.1, 1.8)	1.0 (0.1, 2.1)	-1.1 (-3.8, 1.4)	-5.6 (-6.6, -4.2)
Nigeria	NA	NA	NA	NA	NA	NA	NA	NA
Nepal	NA	NA	NA	NA	NA	NA	NA	NA
Pakistan	NA	NA	NA	NA	NA	NA	NA	NA
Rwanda	13.7 (1.4, 26.7)	13.1 (1.4, 25.5)	13.3 (1.7, 26.0)	15.9 (6.5, 26.9)	0.1 (0.0, 0.1)	0.9 (0.1, 1.9)	-0.6 (-4.0, 2.4)	-6.0 (-7.3, -4.8)
Senegal	12.9 (0.8, 25.3)	12.8 (0.7, 25.1)	13.0 (0.9, 25.6)	13.5 (3.7, 25.0)	0.2 (0.0, 0.5)	0.7 (0.1, 1.6)	-0.6 (-3.8, 2.2)	-3.9 (-5.9, -1.5)
Sierra Leone	12.8 (1.6, 24.8)	12.7 (1.6, 24.7)	12.8 (1.9, 24.9)	13.6 (5.1, 24.7)	0.0 (0.0, 0.0)	0.6 (0.1, 1.2)	-0.6 (-3.6, 1.9)	-3.7 (-4.9, -1.8)
Chad	NA	NA	NA	NA	NA	NA	NA	NA
Togo	12.4 (1.2, 24.3)	11.3 (1.0, 22.2)	13.1 (1.3, 25.8)	20.8 (12.5, 30.2)	0.1 (0.0, 0.3)	0.8 (0.1, 1.8)	-1.1 (-4.1, 1.3)	-6.2 (-7.9, -4.5)
Tajikistan	13.5 (1.7, 26.3)	13.4 (1.7, 26.0)	13.5 (1.9, 25.9)	14.1 (5.0, 26.1)	-0.1 (-0.3, 0.0)	0.4 (0.0, 0.9)	-0.2 (-3.7, 3.0)	-2.5 (-3.3, -1.4)
Timor-Leste	NA	NA	NA	NA	NA	NA	NA	NA
Tanzania	13.5 (1.7, 26.3)	13.3 (1.7, 25.9)	13.2 (1.8, 25.6)	14.2 (5.2, 26.1)	0.2 (0.0, 0.5)	0.8 (0.1, 1.8)	-0.6 (-3.8, 2.4)	-5.4 (-6.4, -4.1)
Uganda	2.5 (0.0, 7.7)	2.5 (0.0, 7.7)	2.5 (0.0, 7.7)	7.6 (5.2, 10.3)	0.0 (0.0, 0.0)	0.2 (0.0, 0.3)	-0.1 (-0.7, 0.3)	-2.3 (-3.3, -1.5)
Yemen	12.1 (1.6, 23.6)	12.0 (1.6, 23.4)	12.5 (2.0, 24.2)	14.6 (8.8, 24.0)	0.9 (0.1, 1.9)	1.3 (0.2, 2.8)	-1.1 (-4.1, 1.6)	-7.6 (-8.8, -5.8)
Zambia	12.6 (1.7, 24.6)	12.5 (1.6, 24.4)	12.7 (2.0, 24.7)	13.6 (5.5, 24.5)	0.3 (0.0, 0.7)	0.8 (0.1, 1.6)	-0.7 (-3.9, 2.1)	-4.6 (-5.6, -3.4)
Zimbabwe	13.1 (1.6, 25.5)	12.2 (1.4, 23.7)	13.2 (2.1, 25.5)	19.0 (10.3, 29.0)	0.3 (0.0, 0.6)	0.9 (0.1, 1.9)	-1.0 (-4.2, 1.4)	-6.5 (-8.1, -5.2)

N/A: estimates not available due to no vaccine introduction in 2016.

Table S5.4. Change in the distribution of measles deaths by wealth quintile for four scenarios, compared to when no treatment is available (Scenario 1).

	Total cases averted (%)				Change in AUC (compared to S1) (%)			
Country	S2	S3	S4	S5	S2 AUC	S3 AUC	S4 AUC	S5 AUC
Armenia	19.4 (5.5, 30.9)	20.0 (5.8, 31.9)	20.6 (6.3, 32.7)	20.0 (5.9, 31.7)	3.1 (0.8, 5.6)	0.0 (0.0, 0.0)	-2.2 (-5.9, 2.1)	-4.5 (-7.2, -1.7)
Burundi	33.5 (9.9, 53.2)	33.6 (9.9, 53.4)	34.3 (10.2, 54.3)	33.6 (10.0, 53.3)	0.5 (0.1, 1.0)	0.0 (0.0, 0.0)	-3.8 (-11.7, 4.5)	-6.0 (-7.5, -4.6)
Benin	20.1 (5.6, 32.0)	20.3 (5.7, 32.4)	20.7 (6.0, 33.0)	20.3 (6.0, 32.2)	1.8 (0.4, 3.1)	0.0 (0.0, 0.0)	-1.9 (-5.5, 2.4)	-4.9 (-6.1, -3.6)
Burkina Faso	29.6 (9.0, 47.0)	30.3 (9.2, 48.1)	30.9 (8.9, 48.8)	30.3 (9.0, 48.0)	4.6 (1.0, 9)	0.0 (0.0, 0.0)	-3.3 (-9.7, 3.7)	-2.1 (-5.9, 2.6)
Bangladesh	18.2 (5.3, 29.0)	19.1 (5.5, 30.6)	19.6 (5.6, 31.1)	19.1 (5.7, 30.3)	3.8 (1.0, 6.7)	0.0 (0.0, 0.0)	-2.0 (-5.4, 1.8)	-3.7 (-7.1, -0.2)
Cote d'Ivoire	18.8 (5.0, 30.2)	19.4 (5.3, 31.0)	19.9 (6.2, 31.8)	19.4 (5.7, 30.7)	2.6 (0.6, 4.6)	0.0 (0.0, 0.0)	-2.2 (-5.7, 1.9)	-5.7 (-8.0, -3.4)
Cameroon	15.9 (3.7, 26.5)	17.4 (4.4, 28.2)	19.0 (5.5, 30.3)	17.4 (5.2, 27.6)	3.1 (0.8, 5.4)	0.0 (0.0, 0.0)	-3.2 (-6.8, 0.7)	-10.7 (-13.7, -7.8)
DRC	24.2 (7.2, 38.5)	24.1 (7.2, 38.2)	25.7 (7.3, 40.7)	24.1 (7.1, 38.2)	0.1 (0.0, 0.3)	0.0 (0.0, 0.0)	-3.4 (-7.6, 1.1)	-12.1 (-12.9, -11.5)
Congo	26.1 (7.6, 41.5)	27.0 (7.8, 43.0)	27.9 (8.1, 44.1)	27.0 (8.0, 42.8)	3.4 (0.8, 6.5)	0.0 (0.0, 0.0)	-3.6 (-8.9, 2.4)	-5.1 (-8.7, -1.3)
Comoros	23.8 (6.9, 37.9)	23.6 (6.8, 37.6)	24.4 (7.1, 38.6)	23.6 (7.0, 37.4)	-0.1 (-0.2, 0.0)	0.0 (0.0, 0.0)	-2.9 (-7.6, 2.3)	-8.7 (-9.7, -7.4)
Ethiopia	18.4 (5.9, 28.9)	19.7 (6.2, 31.0)	20.4 (6.9, 31.8)	19.7 (6.3, 30.7)	4.2 (1.1, 7.3)	0.0 (0.0, 0.0)	-2.2 (-5.9, -1.6)	-4.3 (-7.9, -0.4)
Ghana	26.9 (7.6, 42.7)	26.5 (7.5, 42.0)	27.2 (8.1, 43.0)	26.5 (7.8, 42.0)	-1.7 (-3.3, -0.4)	0.0 (0.0, 0.0)	-2.9 (-8.6, 3.4)	-8.0 (-9.8, -6.3)
Guinea	21.8 (6.2, 34.9)	23.7 (6.9, 37.8)	24.8 (7.2, 39.2)	23.7 (7.0, 37.6)	5.3 (1.3, 9.6)	0.0 (0.0, 0.0)	-3.1 (-7.2, 1.7)	-3.8 (-8.7, 1.5)
Gambia	40.4 (12.1, 64.2)	40.3 (12.1, 64.1)	40.6 (11.5, 64.7)	40.3 (12.0, 64.0)	-0.9 (-2.1, -0.2)	0.0 (0.0, 0.0)	-2.1 (-11.4, 7.8)	-3.6 (-5.0, -2.4)
Honduras	34.9 (10.6, 55.4)	34.9 (10.6, 55.5)	35.1 (10.2, 55.8)	34.9 (10.4, 55.4)	1.1 (0.2, 2.4)	0.0 (0.0, 0.0)	-2.3 (-10.7, 7.1)	-2.1 (-3.4, -0.7)
Haiti	21.8 (6.5, 34.7)	22.1 (6.6, 35.2)	22.2 (6.6, 35.3)	22.1 (6.6, 35.1)	3.9 (0.9, 7.1)	0.0 (0.0, 0.0)	-1.1 (-5.0, 3.3)	0.6 (-2.6, 4.0)
Indonesia	41.9 (12.8, 66.6)	42.0 (12.8, 66.7)	42.9 (12.8, 68.0)	42.0 (12.5, 66.6)	1.3 (0.2, 3.1)	0.0 (0.0, 0.0)	-5.9 (-17.7, 5.9)	-6.0 (-7.1, -4.6)
Kenya	36.8 (11.3, 58.5)	36.8 (11.3, 58.6)	38.0 (11.2, 60.3)	36.8 (10.9, 58.4)	0.1 (0.0, 0.2)	0.0 (0.0, 0.0)	-5.8 (-15.3, 4.0)	-8.5 (-9.6, -7.5)
Kyrgyz Republic	30.5 (9.1, 48.6)	30.6 (9.1, 48.7)	30.7 (8.9, 48.7)	30.6 (9.1, 48.5)	5.3 (1.1, 10.5)	0.0 (0.0, 0.0)	-1.0 (-7.8, 6.7)	3.8 (-0.5, 9.1)
Cambodia	37.0 (11.3, 58.8)	36.9 (11.3, 58.6)	37.6 (11.2, 59.6)	36.9 (10.9, 58.5)	-1.2 (-2.6, -0.2)	0.0 (0.0, 0.0)	-4.5 (-13.9, 4.9)	-8.0 (-9.7, -6.7)
Liberia	29.3 (8.8, 46.6)	29.2 (8.8, 46.4)	29.8 (8.6, 47.2)	29.2 (8.7, 46.3)	-0.2 (-0.4, 0.0)	0.0 (0.0, 0.0)	-3.3 (-9.3, 3.2)	-7.7 (-8.4, -7.1)
Lesotho	34.1 (10, 54.2)	34.0 (10.0, 54.0)	35.2 (10.6, 55.6)	34.0 (10.1, 53.9)	-0.6 (-1.2, -0.1)	0.0 (0.0, 0.0)	-5.1 (-13.2, 3.2)	-9.3 (-11.3, -7.3)
Mali	18.4 (5.4, 29.3)	19.6 (5.9, 31.3)	20.5 (5.7, 32.6)	19.6 (5.8, 31.1)	3.5 (0.9, 6.2)	0.0 (0.0, 0.0)	-2.4 (-5.6, 1.3)	-6.0 (-9.0, -2.9)
Mozambique	31.3 (9.3, 49.8)	32.4 (9.7, 51.6)	35.1 (10.7, 55.6)	32.4 (9.6, 51.4)	2.9 (0.6, 5.8)	0.0 (0.0, 0.0)	-7.1 (-15.4, 1.4)	-10.3 (-12.9, -6.9)
Malawi	39.3 (11.6, 62.6)	39.5 (11.7, 62.8)	41.0 (11.8, 65.0)	39.5 (11.7, 62.6)	1.0 (0.2, 2.2)	0.0 (0.0, 0.0)	-6.8 (-17.8, 4.0)	-8.5 (-10.5, -6.3)
Niger	30.9 (8.8, 49.3)	31.6 (9.0, 50.3)	32.4 (9.3, 51.4)	31.6 (9.4, 50.2)	4.4 (0.9, 8.8)	0.0 (0.0, 0.0)	-3.8 (-10.9, 3.9)	-2.8 (-6.3, 1.8)
Nigeria	20.2 (6.1, 31.7)	21.2 (6.6, 33.5)	22.2 (7.3, 34.6)	21.3 (6.8, 33.1)	3.4 (0.9, 6.0)	0.0 (0.0, 0.0)	-2.8 (-7.3, 1.6)	-6.4 (-9.3, -3.2)
Nepal	22.7 (6.3, 36.3)	22.8 (6.3, 36.5)	23.6 (6.8, 37.6)	22.8 (6.8, 36.2)	1.0 (0.2, 1.8)	0.0 (0.0, 0.0)	-2.6 (-6.7, 2.0)	-7.8 (-10.0, -5.9)
Pakistan	37.7 (11.9, 58.9)	38.8 (12.2, 60.6)	41.3 (13.7, 64.5)	38.8 (12.5, 60.3)	4.3 (0.9, 9.1)	0.0 (0.0, 0.0)	-9.1 (-23.1, 3.6)	-7.1 (-11.1, -1.5)
Rwanda	34.2 (10.1, 54.4)	34.7 (10.4, 55.1)	35.3 (10.4, 56.0)	34.7 (10.3, 55.1)	3.5 (0.7, 7.4)	0.0 (0.0, 0.0)	-3.9 (-12.2, 4.6)	-2.8 (-5.8, 1.5)
Senegal	22.6 (6.6, 36.0)	22.5 (6.5, 35.8)	23.0 (6.7, 36.6)	22.5 (6.7, 35.6)	-0.5 (-0.8, -0.1)	0.0 (0.0, 0.0)	-2.5 (-6.7, 2.4)	-8.1 (-9.7, -6.6)
Sierra Leone	40.9 (12.3, 65.0)	41.0 (12.4, 65.2)	41.5 (11.9, 65.9)	41.0 (12.2, 65.1)	1.4 (0.3, 3.3)	0.0 (0.0, 0.0)	-3.3 (-13.6, 6.3)	-2.7 (-4.2, -0.7)
Chad	15.7 (4.6, 25.1)	15.9 (4.7, 25.3)	15.9 (4.4, 25.4)	15.9 (4.7, 25.2)	3.1 (0.8, 5.4)	0.0 (0.0, 0.0)	-0.2 (-2.6, 2.5)	2.5 (0.0, 5.0)
Togo	23.3 (6.8, 37.0)	24.1 (7.0, 38.4)	25.0 (7.1, 39.7)	24.1 (7.1, 38.2)	1.5 (0.3, 2.7)	0.0 (0.0, 0.0)	-2.6 (-6.6, 1.9)	-6.7 (-8.5, -4.8)
Tajikistan	32.2 (9.8, 51.3)	32.3 (9.8, 51.4)	32.5 (9.5, 51.5)	32.3 (9.6, 51.3)	1.8 (0.4, 3.6)	0.0 (0.0, 0.0)	-1.0 (-8.4, 7.3)	0.3 (-1.2, 2.1)
Timor-Leste	42.7 (12.9, 67.8)	42.9 (12.9, 68.2)	43.6 (12.7, 69.2)	42.9 (12.7, 68.1)	2.0 (0.3, 4.7)	0.0 (0.0, 0.0)	-5.1 (-16.5, 6.4)	-4.0 (-5.8, -1.3)
Tanzania	31.6 (9.5, 50.2)	31.8 (9.6, 50.5)	32.5 (9.4, 51.5)	31.8 (9.4, 50.5)	1.3 (0.3, 2.6)	0.0 (0.0, 0.0)	-3.8 (-11.1, 4.1)	-5.5 (-6.9, -3.9)
Uganda	45.0 (13.6, 71.6)	45.0 (13.6, 71.5)	45.4 (12.9, 72.3)	45.0 (13.3, 71.4)	-0.4 (-0.9, -0.1)	0.0 (0.0, 0.0)	-3.8 (-16, 8.7)	-4.6 (-5.6, -3.7)
Yemen	20.0 (5.9, 31.9)	20.4 (6.0, 32.6)	21.7 (6.5, 34.2)	20.4 (6.1, 32.4)	1.3 (0.3, 2.2)	0.0 (0.0, 0.0)	-3.3 (-7.3, 1.3)	-9.9 (-11.3, -8.5)
Zambia	40.5 (12.2, 64.4)	40.6 (12.3, 64.6)	41.4 (12.3, 65.8)	40.6 (12.1, 64.5)	1.4 (0.3, 3.3)	0.0 (0.0, 0.0)	-5.0 (-16.1, 6.9)	-4.6 (-6.0, -2.6)
Zimbabwe	24.9 (7.4, 39.6)	24.8 (7.4, 39.5)	25.3 (7.5, 40.0)	24.8 (7.4, 39.4)	-0.4 (-0.7, -0.1)	0.0 (0.0, 0.0)	-2.2 (-6.8, 2.9)	-6.6 (-7.1, -6.1)

Table S5.5. Change in the distribution of pneumonia deaths by wealth quintile for four scenarios, compared to when no treatment is available (Scenario 1).

	Total cases averted (%)				Change in AUC (compared to S1) (%)			
Country	S2	S3	S4	S5	S2 AUC	S3 AUC	S4 AUC	S5 AUC
Armenia	20.5 (13.1, 27.5)	21.8 (14.0, 28.9)	24.7 (17.0, 31.8)	21.8 (17.6, 26.1)	2.9 (2.2, 3.7)	0.0 (0.0, 0.0)	-3.9 (-6.0, -2.3)	-11.3 (-13.2, -8.3)
Burundi	35.7 (28.0, 43.4)	35.7 (28.0, 43.4)	35.9 (29.0, 42.9)	35.7 (28.9, 42.7)	1.0 (0.7, 1.3)	0.0 (0.0, 0.0)	-1.4 (-4.7, 1.7)	-1.3 (-3.8, 0.8)
Benin	20.1 (14.2, 25.5)	20.3 (14.4, 25.7)	20.9 (15.7, 25.8)	20.3 (16.4, 24.2)	1.8 (1.4, 2.3)	0.0 (0.0, 0.0)	-1.9 (-3.5, -0.7)	-5.6 (-6.1, -5.1)
Burkina Faso	34.5 (27.7, 41.4)	35.2 (28.3, 42.2)	35.6 (28.9, 42.4)	35.2 (28.5, 42.1)	6.1 (4.5, 8.1)	0.0 (0.0, 0.0)	-2.8 (-5.9, -0.3)	1.0 (-0.8, 3.1)
Bangladesh	23.5 (18.8, 28.4)	24.4 (19.5, 29.6)	24.8 (19.9, 29.7)	24.4 (19.7, 29.2)	5.1 (3.8, 6.5)	0.0 (0.0, 0.0)	-1.9 (-3.8, -0.4)	-0.6 (-2.1, 1.0)
Cote d'Ivoire	24.0 (18.1, 29.7)	24.4 (18.9, 29.9)	24.5 (19.5, 29.7)	24.4 (19.7, 29.2)	4.6 (3.5, 5.9)	0.0 (0.0, 0.0)	-1.1 (-3.0, 0.5)	1.1 (-0.4, 2.7)
Cameroon	18.9 (10.4, 27.4)	21.0 (13.0, 29.0)	23.4 (16.5, 29.9)	21.0 (17.0, 25.2)	3.8 (2.9, 4.8)	0.0 (0.0, 0.0)	-4.4 (-6.5, -2.4)	-11.8 (-15.5, -7.4)
DRC	27.0 (20.8, 33.1)	27.1 (20.9, 33.3)	28.2 (22.0, 34.4)	27.1 (22.0, 32.5)	0.9 (0.6, 1.1)	0.0 (0.0, 0.0)	-3.0 (-4.9, -1.4)	-8.6 (-10, -6.9)
Congo	34.2 (26.3, 42.0)	34.8 (26.8, 42.8)	35.3 (27.8, 42.8)	34.8 (27.5, 41.8)	5.6 (4.0, 7.4)	0.0 (0.0, 0.0)	-3.1 (-6.5, -0.3)	0.1 (-3.2, 3.4)
Comoros	27.3 (20.5, 33.7)	27.4 (20.7, 33.9)	28.3 (22.0, 34.3)	27.4 (22.2, 32.8)	1.0 (0.7, 1.3)	0.0 (0.0, 0.0)	-3.3 (-5.6, -1.4)	-7.6 (-9.6, -4.9)
Ethiopia	19.1 (13.9, 24.5)	20.5 (14.4, 26.1)	21.2 (15.8, 26.1)	20.5 (16.5, 24.5)	5.4 (4.1, 6.9)	0.0 (0.0, 0.0)	-2.1 (-3.5, -0.8)	-2.6 (-5.1, 0.5)
Ghana	32.8 (24.4, 41.0)	32.7 (24.4, 40.9)	33.2 (25.9, 40.3)	32.7 (26.4, 39.1)	-0.9 (-1.1, -0.6)	0.0 (0.0, 0.0)	-1.6 (-5.1, 1.5)	-3.6 (-7.0, -0.5)
Guinea	23.8 (17.6, 30.6)	26.6 (19.8, 33.3)	27.8 (21.5, 33.9)	26.6 (21.5, 31.8)	8.0 (6.0, 10.3)	0.0 (0.0, 0.0)	-3.5 (-5.5, -1.6)	-0.6 (-3.7, 3.0)
Gambia	44.3 (35.4, 53.2)	44.2 (35.4, 53.1)	44.8 (36.3, 53.3)	44.2 (35.7, 52.8)	-1.3 (-1.7, -0.9)	0.0 (0.0, 0.0)	-0.1 (-3.9, 3.1)	-1.5 (-2.3, -0.9)
Honduras	42.5 (33.6, 51.6)	42.4 (33.5, 51.3)	42.7 (34.3, 50.8)	42.4 (34.3, 50.7)	2.6 (1.8, 3.6)	0.0 (0.0, 0.0)	1.8 (-2.8, 5.6)	5.2 (1.7, 8.0)
Haiti	25.1 (18.8, 31.2)	25.2 (18.7, 31.5)	25.7 (19.7, 31.6)	25.2 (20.4, 30.1)	5.1 (3.8, 6.4)	0.0 (0.0, 0.0)	-0.5 (-2.2, 0.9)	2.4 (0.7, 4.2)
Indonesia	49.6 (39.7, 59.3)	49.7 (39.9, 59.5)	50.4 (40.7, 60.2)	49.7 (40.2, 59.4)	2.9 (1.9, 4.3)	0.0 (0.0, 0.0)	-4.5 (-10.7, 0.2)	-2.0 (-3.3, -0.6)
Kenya	42.9 (34.3, 51.7)	43.1 (34.5, 51.9)	44.4 (35.9, 52.9)	43.1 (34.9, 51.6)	1.4 (1.0, 2.0)	0.0 (0.0, 0.0)	-6.1 (-11, -2.5)	-6.4 (-8.0, -5.2)
Kyrgyz Republic	33.1 (25.3, 40.9)	33.3 (25.5, 41.0)	33.4 (26.6, 40.2)	33.3 (27.0, 39.9)	5.5 (4.0, 7.3)	0.0 (0.0, 0.0)	-0.8 (-3.7, 1.8)	4.0 (1.4, 6.9)
Cambodia	44.7 (35.2, 54.0)	44.8 (35.4, 54.1)	45.0 (36.3, 53.7)	44.8 (36.2, 53.6)	0.0 (0.0, 0.1)	0.0 (0.0, 0.0)	-2.2 (-7.5, 2.4)	-2.5 (-4.5, 0.0)
Liberia	32.9 (25.6, 40.2)	32.8 (25.6, 40.0)	33.1 (26.3, 39.6)	32.8 (26.5, 39.3)	-1.4 (-1.8, -1.0)	0.0 (0.0, 0.0)	-2.0 (-4.8, 0.5)	-5.6 (-7.4, -3.7)
Lesotho	41.1 (31.4, 50.4)	41.1 (31.6, 50.4)	42.0 (33.7, 50.3)	41.1 (33.3, 49.2)	0.4 (0.3, 0.6)	0.0 (0.0, 0.0)	-5.2 (-10.4, -0.7)	-6.7 (-11.1, -2.9)
Mali	20.8 (15.8, 25.9)	22.3 (16.8, 27.5)	23.0 (17.9, 28.0)	22.3 (18.0, 26.7)	5.0 (3.8, 6.3)	0.0 (0.0, 0.0)	-2.3 (-3.9, -1.0)	-3.2 (-5.0, -1.2)
Mozambique	32.6 (25.4, 40.0)	33.9 (26.6, 41.5)	36.9 (29.7, 44.4)	33.9 (27.4, 40.6)	3.5 (2.5, 4.5)	0.0 (0.0, 0.0)	-7.5 (-11.2, -4.6)	-10.2 (-12.1, -8.0)
Malawi	45.1 (34.4, 55.3)	45.7 (34.8, 56.0)	49.0 (39.1, 58.9)	45.7 (37.0, 54.7)	2.8 (1.9, 3.9)	0.0 (0.0, 0.0)	-10.8 (-16.8, -5.4)	-10.2 (-13.7, -5.5)
Niger	34.2 (26.5, 41.8)	35.1 (27.5, 42.6)	36.2 (29.1, 43.4)	35.1 (28.4, 42.0)	7.7 (5.7, 10.3)	0.0 (0.0, 0.0)	-4.1 (-7.5, -1.5)	0.7 (-1.6, 3.2)
Nigeria	24.1 (19.4, 29.0)	25.5 (20.5, 30.7)	26.2 (21.0, 31.7)	25.5 (20.6, 30.6)	4.7 (3.5, 6.0)	0.0 (0.0, 0.0)	-2.9 (-4.5, -1.3)	-3.5 (-4.9, -2.0)
Nepal	26.0 (18.7, 33.0)	26.0 (18.7, 33.2)	26.2 (20.1, 32.2)	26.0 (21.0, 31.1)	2.1 (1.6, 2.7)	0.0 (0.0, 0.0)	-0.7 (-2.7, 0.9)	-0.4 (-2.9, 2.1)
Pakistan	41.5 (33.0, 50.1)	42.5 (33.8, 51.3)	44.5 (35.8, 53.7)	42.5 (34.3, 50.9)	4.7 (3.3, 6.5)	0.0 (0.0, 0.0)	-8.2 (-13.4, -3.9)	-5.0 (-6.8, -3.0)
Rwanda	41.9 (33.1, 50.5)	42.1 (33.2, 50.8)	43.3 (34.7, 51.7)	42.1 (34.0, 50.3)	5.6 (3.9, 7.7)	0.0 (0.0, 0.0)	-2.9 (-7.8, 1.3)	1.7 (-0.7, 4.1)
Senegal	27.8 (20.7, 34.9)	27.5 (20.4, 34.4)	27.8 (21.5, 33.8)	27.5 (22.2, 32.9)	-1.6 (-2.1, -1.2)	0.0 (0.0, 0.0)	-1.9 (-4.2, 0.1)	-6.9 (-9.4, -4.2)
Sierra Leone	46.8 (37.6, 56.2)	46.8 (37.6, 56.2)	47.1 (38.1, 56.1)	46.8 (37.8, 56.0)	1.2 (0.8, 1.7)	0.0 (0.0, 0.0)	-1.8 (-6.5, 2.0)	-0.7 (-1.8, 0.6)
Chad	17.5 (13.6, 21.8)	17.5 (13.6, 21.7)	17.7 (13.9, 21.5)	17.5 (14.2, 21.0)	4.0 (3.1, 5.0)	0.0 (0.0, 0.0)	0.2 (-0.8, 1.1)	5.3 (4.2, 6.4)
Togo	31.3 (24.3, 38.3)	32.5 (25.0, 39.8)	33.7 (26.6, 40.8)	32.5 (26.3, 38.9)	2.6 (1.9, 3.5)	0.0 (0.0, 0.0)	-3.0 (-5.6, -1.1)	-4.3 (-5.5, -3.0)
Tajikistan	36.5 (28.8, 44.5)	36.6 (29.0, 44.4)	37.0 (29.7, 44.1)	36.6 (29.6, 43.8)	3.0 (2.2, 4.0)	0.0 (0.0, 0.0)	-0.9 (-4.7, 2.2)	1.6 (-0.1, 3.8)
Timor-Leste	46.5 (37.4, 55.9)	46.8 (37.6, 56.3)	47.4 (38.4, 56.5)	46.8 (37.9, 56.0)	3.5 (2.4, 4.9)	0.0 (0.0, 0.0)	-4.4 (-9.7, -0.3)	-1.5 (-3.2, 0.7)
Tanzania	38.6 (30.6, 46.6)	38.6 (30.7, 46.7)	39.0 (31.5, 46.7)	38.6 (31.3, 46.2)	3.1 (2.2, 4.2)	0.0 (0.0, 0.0)	-1.9 (-6.0, 1.7)	0.5 (-1.4, 2.3)
Uganda	51.1 (40.5, 61.5)	51.2 (40.6, 61.6)	51.7 (41.6, 61.7)	51.2 (41.4, 61.2)	0.5 (0.3, 0.7)	0.0 (0.0, 0.0)	-2.1 (-7.6, 2.5)	-1.6 (-2.7, -0.4)
Yemen	22.4 (17.2, 27.7)	22.7 (17.6, 28.1)	23.5 (18.7, 28.4)	22.7 (18.4, 27.2)	1.5 (1.2, 1.9)	0.0 (0.0, 0.0)	-2.8 (-4.7, -1.2)	-7.0 (-8.1, -5.6)
Zambia	46.0 (36.8, 55.4)	46.1 (36.9, 55.5)	46.8 (38.0, 55.9)	46.1 (37.3, 55.2)	3.3 (2.2, 4.6)	0.0 (0.0, 0.0)	-3.6 (-9.1, 0.7)	-0.3 (-1.7, 1.3)
Zimbabwe	30.9 (24.3, 37.6)	30.5 (23.9, 37.1)	31.5 (25.1, 37.9)	30.5 (24.6, 36.4)	-1.5 (-1.9, -1.1)	0.0 (0.0, 0.0)	-3.0 (-5.4, -1.0)	-9.8 (-10.7, -9.0)

Table S5.6. Change in the distribution of diarrhea deaths by wealth quintile for four scenarios, compared to when no treatment is available (Scenario 1).

	Total cases averted (%)				Change in AUC (compared to S1) (%)			
Country	S2	S3	S4	S5	S2 AUC	S3 AUC	S4 AUC	S5 AUC
Armenia	29.0 (23.1, 34.4)	30.9 (24.8, 36.4)	35.2 (29.7, 39.8)	30.9 (27.8, 33.3)	4.6 (3.9, 5.3)	0.0 (0.0, 0.0)	-6.7 (-8.6, -4.7)	-9.5 (-12, -6.5)
Burundi	53.3 (47.5, 57.8)	53.2 (47.5, 57.8)	53.6 (48.3, 57.8)	53.2 (47.9, 57.3)	0.6 (0.4, 0.7)	0.0 (0.0, 0.0)	-3.9 (-7.2, -0.6)	-2.8 (-4.2, -1.5)
Benin	33.3 (28.9, 36.9)	34.1 (29.6, 37.7)	35.2 (31.3, 38.4)	34.1 (30.6, 36.7)	3.3 (2.8, 3.7)	0.0 (0.0, 0.0)	-4.5 (-5.8, -3.2)	-5.7 (-6.4, -5.1)
Burkina Faso	42.8 (38.2, 46.2)	43.7 (39.0, 47.3)	44.5 (40.0, 48.0)	43.7 (39.2, 47.0)	6.6 (5.5, 7.5)	0.0 (0.0, 0.0)	-4.9 (-6.6, -3.1)	-0.1 (-1.6, 1.1)
Bangladesh	23.2 (20.5, 25.5)	24.5 (21.5, 27.0)	25.1 (22.2, 27.4)	24.5 (22.0, 26.4)	5.2 (4.5, 5.8)	0.0 (0.0, 0.0)	-2.4 (-3.2, -1.7)	-1.9 (-3.1, -0.9)
Cote d'Ivoire	24.9 (20.8, 28.4)	25.2 (21.3, 28.7)	25.6 (22.3, 28.4)	25.2 (22.7, 27.2)	1.8 (1.6, 2.0)	0.0 (0.0, 0.0)	-2.2 (-3.2, -1.2)	-4.8 (-6.9, -3.3)
Cameroon	21.4 (14.1, 28.3)	23.9 (16.9, 30.7)	27.0 (21.8, 32.0)	23.9 (21.5, 25.8)	4.2 (3.6, 4.8)	0.0 (0.0, 0.0)	-5.4 (-7.1, -3.8)	-11.9 (-15.9, -8.1)
DRC	36.5 (31.5, 40.7)	35.9 (31.1, 40.0)	37.3 (32.9, 40.9)	35.9 (32.3, 38.7)	-1.1 (-1.4, -0.9)	0.0 (0.0, 0.0)	-4.6 (-5.9, -3.2)	-11.0 (-12.8, -9.2)
Congo	33.0 (28.2, 37.1)	34.1 (29.1, 38.3)	35.1 (31.1, 38.6)	34.1 (30.6, 36.7)	4.0 (3.3, 4.5)	0.0 (0.0, 0.0)	-4.6 (-6.2, -2.9)	-4.4 (-7.0, -2.2)
Comoros	36.7 (31.8, 40.5)	36.5 (31.7, 40.3)	37.2 (33.1, 40.5)	36.5 (32.8, 39.3)	-0.3 (-0.4, -0.2)	0.0 (0.0, 0.0)	-4.3 (-6.0, -2.4)	-7.7 (-9.7, -5.6)
Ethiopia	29.4 (25.0, 33.6)	30.9 (26.2, 35.4)	32.0 (27.8, 35.5)	30.9 (27.9, 33.4)	6.3 (5.3, 7.3)	0.0 (0.0, 0.0)	-3.8 (-5.0, -2.5)	-1.7 (-4.2, 0.0)
Ghana	41.4 (35.9, 46.2)	40.9 (35.3, 45.7)	41.3 (37.0, 44.7)	40.9 (36.8, 44.0)	-3.1 (-3.5, -2.5)	0.0 (0.0, 0.0)	-2.7 (-5.4, -0.3)	-6.3 (-9.1, -3.9)
Guinea	33.6 (28.7, 37.7)	35.5 (30.5, 39.9)	37.4 (32.9, 41.1)	35.5 (32.0, 38.3)	6.1 (5.1, 7.0)	0.0 (0.0, 0.0)	-5.8 (-7.4, -4.0)	-4.1 (-6.5, -1.8)
Gambia	62.1 (55.5, 67.0)	62.1 (55.6, 67.0)	62.8 (56.6, 67.8)	62.1 (55.8, 66.9)	-1.3 (-1.6, -1.0)	0.0 (0.0, 0.0)	1.5 (-2.3, 4.8)	-0.5 (-1.2, 0.4)
Honduras	47.8 (41.8, 52.5)	47.9 (41.8, 52.6)	48.0 (43.2, 51.7)	47.9 (43.1, 51.6)	0.1 (0.1, 0.2)	0.0 (0.0, 0.0)	0.5 (-3.5, 3.8)	0.6 (-3.2, 3.4)
Haiti	31.6 (26.6, 35.9)	32.6 (27.4, 37.2)	33.5 (29.2, 37.2)	32.6 (29.3, 35.1)	4.9 (4.1, 5.6)	0.0 (0.0, 0.0)	-2.5 (-3.9, -1.1)	-0.9 (-3.5, 1.1)
Indonesia	59.3 (53.0, 64.2)	59.4 (53.0, 64.2)	60.7 (54.7, 65.4)	59.4 (53.4, 64.0)	-0.4 (-0.5, -0.3)	0.0 (0.0, 0.0)	-8.5 (-12.7, -4.5)	-6.1 (-7.7, -4.7)
Kenya	53.0 (47.3, 57.4)	52.7 (47.0, 57.1)	54.8 (49.3, 59.4)	52.7 (47.4, 56.7)	-2.0 (-2.4, -1.6)	0.0 (0.0, 0.0)	-11.2 (-15.0, -7.7)	-11.3 (-12.8, -10.3)
Kyrgyz Republic	46.5 (40.6, 51.5)	47.3 (41.4, 52.1)	47.5 (42.7, 51.2)	47.3 (42.5, 50.9)	9.1 (7.4, 10.5)	0.0 (0.0, 0.0)	-2.9 (-5.3, -0.6)	6.5 (4.1, 8.6)
Cambodia	50.6 (44.2, 55.7)	50.5 (44.2, 55.4)	50.8 (45.9, 54.9)	50.5 (45.4, 54.4)	-5.2 (-6.2, -4.1)	0.0 (0.0, 0.0)	-2.8 (-6.9, 1.3)	-7.7 (-10.5, -4.7)
Liberia	43.6 (38.4, 47.7)	43.7 (38.5, 47.7)	44.0 (39.5, 47.4)	43.7 (39.2, 47.0)	1.3 (1.1, 1.5)	0.0 (0.0, 0.0)	-3.4 (-5.6, -0.9)	-3.2 (-5.1, -1.3)
Lesotho	47.5 (41.3, 52.6)	46.7 (40.5, 52.0)	49.5 (44.2, 54.3)	46.7 (42.0, 50.3)	-2.7 (-3.1, -2.2)	0.0 (0.0, 0.0)	-11.0 (-15.7, -7.0)	-13.5 (-17, -10.7)
Mali	27.7 (23.8, 31.0)	29.0 (24.9, 32.4)	30.1 (26.5, 33.1)	29.0 (26.1, 31.2)	4.5 (3.9, 5.0)	0.0 (0.0, 0.0)	-3.6 (-4.5, -2.6)	-4.6 (-6.1, -3.2)
Mozambique	50.7 (44.9, 55.3)	52.1 (46.3, 56.8)	56.2 (50.3, 61.4)	52.1 (46.9, 56.1)	5.0 (4.1, 5.9)	0.0 (0.0, 0.0)	-15.5 (-20.3, -11.1)	-8.1 (-10.1, -6.1)
Malawi	57.4 (50.6, 62.6)	57.1 (50.3, 62.3)	61.1 (54.7, 66.7)	57.1 (51.4, 61.5)	-1.4 (-1.7, -1.0)	0.0 (0.0, 0.0)	-17.8 (-24.1, -12.3)	-14.3 (-16.9, -11.5)
Niger	47.4 (42.1, 51.4)	48.0 (42.7, 52.0)	49.4 (44.4, 53.4)	48.0 (43.1, 51.7)	3.6 (3.0, 4.2)	0.0 (0.0, 0.0)	-7.3 (-9.6, -4.9)	-4.6 (-5.3, -3.9)
Nigeria	27.5 (24.6, 30.0)	28.5 (25.5, 31.1)	29.3 (26.3, 31.8)	28.5 (25.8, 30.8)	4.4 (3.9, 4.9)	0.0 (0.0, 0.0)	-3.2 (-4.1, -2.3)	-4.1 (-4.9, -3.1)
Nepal	35.2 (29.9, 40.0)	35.3 (30.0, 40.1)	35.8 (31.4, 39.3)	35.3 (31.7, 38.0)	1.7 (1.4, 1.9)	0.0 (0.0, 0.0)	-2.7 (-4.2, -1.1)	-4.1 (-6.4, -1.8)
Pakistan	56.0 (50.01 60.8)	57.6 (51.6, 62.5)	60.5 (54.6, 65.4)	57.6 (52.1, 62.1)	8.3 (6.6, 10.0)	0.0 (0.0, 0.0)	-16.3 (-21.1, -12.0)	-1.7 (-4.0, 0.6)
Rwanda	46.9 (41.7, 51.1)	47.6 (42.2, 51.9)	48.9 (44.0, 52.8)	47.6 (42.8, 51.3)	5.8 (4.8, 6.7)	0.0 (0.0, 0.0)	-5.1 (-7.7, -2.3)	0.3 (-1.6, 2.0)
Senegal	30.3 (25.8, 34.5)	30.5 (25.9, 34.7)	30.6 (26.9, 33.7)	30.5 (27.4, 32.8)	1.0 (0.8, 1.2)	0.0 (0.0, 0.0)	-1.2 (-2.7, 0.2)	-1.8 (-4.2, 0.4)
Sierra Leone	60.2 (53.8, 65.0)	60.5 (54.1, 65.3)	60.9 (54.9, 65.7)	60.5 (54.4, 65.1)	3.7 (2.9, 4.5)	0.0 (0.0, 0.0)	-4.9 (-8.5, -1.4)	0.2 (-1.1, 1.3)
Chad	23.9 (20.8, 26.5)	24.1 (20.9, 26.8)	24.3 (21.5, 26.7)	24.1 (21.7, 26.0)	4.3 (3.7, 4.8)	0.0 (0.0, 0.0)	-0.2 (-0.9, 0.4)	3.5 (2.3, 4.4)
Togo	27.3 (22.9, 31.0)	28.4 (23.9, 32.3)	30.3 (26.2, 34.0)	28.4 (25.5, 30.6)	0.8 (0.5, 0.9)	0.0 (0.0, 0.0)	-3.8 (-4.6, -2.8)	-10.4 (-11.9, -9.2)
Tajikistan	49.8 (44.3, 54.2)	50.0 (44.5, 54.3)	50.5 (45.5, 54.4)	50.0 (44.9, 53.8)	3.5 (2.8, 4.1)	0.0 (0.0, 0.0)	-1.0 (-4.1, 2.1)	2.3 (0.6, 4.3)
Timor-Leste	66.1 (59.3, 71.3)	66.3 (59.5, 71.5)	67.1 (60.4, 72.4)	66.3 (59.6, 71.4)	4.6 (3.4, 5.7)	0.0 (0.0, 0.0)	-9.8 (-14.7, -4.8)	-0.5 (-2.2, 1.3)
Tanzania	49.1 (43.5, 53.5)	49.2 (43.5, 53.6)	49.6 (44.7, 53.6)	49.2 (44.2, 53.0)	2.9 (2.4, 3.4)	0.0 (0.0, 0.0)	-3.5 (-7.2, -0.1)	-0.4 (-3.0, 1.8)
Uganda	66.7 (59.5, 72.3)	66.5 (59.3, 72.0)	67.2 (60.4, 73.1)	66.5 (59.8, 71.6)	-2.6 (-3.4, -1.9)	0.0 (0.0, 0.0)	-7.9 (-13.3, -2.9)	-7.4 (-9.3, -5.6)
Yemen	30.5 (26.6, 33.7)	30.9 (27.0, 34.1)	31.9 (28.4, 34.6)	30.9 (27.8, 33.3)	2.3 (2.0, 2.6)	0.0 (0.0, 0.0)	-4.1 (-5.6, -2.8)	-6.2 (-7.5, -4.9)
Zambia	60.2 (53.8, 65.0)	60.2 (53.8, 65.0)	60.9 (54.9, 65.8)	60.2 (54.1, 64.9)	0.0 (-0.2, 0.1)	0.0 (0.0, 0.0)	-7.3 (-11.6, -3.0)	-4.8 (-6.0, -3.8)
Zimbabwe	32.8 (28.6, 36.3)	33.0 (28.8, 36.5)	34.6 (30.7, 37.8)	33.0 (29.6, 35.5)	0.4 (0.3, 0.5)	0.0 (0.0, 0.0)	-4.5 (-5.8, -3.3)	-9.7 (-11.1, -8.6)

VI. Sensitivity analyses

We performed two sets of sensitivity analyses.

1. **Sensitivity analysis 1.** *Accounting for differences in the sizes of the under-five population in each quintile* applying a total fertility rate adjustment

First, we accounted for the differences in the sizes of the under-five population in each quintile by adjusting the number of cases and deaths with the quintile-specific total fertility rate (TFR):

$$\mathrm{C_i} = [\mathrm{Ac_i} \times \mathrm{TC}_a + \mathrm{UAc_i} \times \mathrm{TC}_{ua}] \times \frac{\mathrm{TFR_i}}{\mathrm{TFR_{avg}}} \quad ,$$

$$\mathrm{D_i} = [\mathrm{Ad_i} \times \mathrm{TD}_a + \mathrm{UAd_i} \times \mathrm{TD}_{ua}] \times \frac{\mathrm{TFR_i}}{\mathrm{TFR_{avg}}} \quad .$$

where $\mathrm{TFR_i}$ and $\mathrm{TFR_{avg}}$ are the quintile-specific and national average of TFR, respectively.

Higher TFR in lower wealth quintiles leads to greater number of susceptible children and therefore larger proportions of cases and deaths in these quintiles. We present the full results of the sensitivity analysis for cases and deaths in Tables S6.1 and S6.2, respectively. We highlighted the results with significant differences in the change in the AUC of greater than 25% compared to the main results (presented in Appendix V).

We found that the results were mostly sensitive to this adjustment, mainly because the TFR adjustment makes the underlying distribution of cases and deaths more unequal (more cases and deaths occur in the lower wealth quintiles), and therefore increases the effect of the vaccine/treatment scenarios on the distribution. As expected, the changes in the AUC for all scenarios are more favorable (more equal distribution of cases and deaths) than the main results. We therefore conclude that our main results (presented in the main text and Appendix V) that did not reflect the TFR adjustment are conservative estimates of the effect of vaccines and treatment on the distributions of disease cases and deaths.

2. **Sensitivity analysis 2.** *Replacing the flat distribution of the unattributable cases and deaths with a distribution comparable to the distribution of quintile-specific under-five mortality rates*.

Instead of assuming that unattributable cases and deaths ($\mathrm{UAc_i}$ and $\mathrm{UAd_i}$) follow a flat distribution, we replaced it with the ratios of under-five mortality rates from each quintile ($\mathrm{U5M_i}$) to check the impact of this assumption on our results:

$$\mathrm{UAc_i} = \mathrm{UAd_i} = \frac{\mathrm{U5M}_i}{\sum_{j=1}^{5} \mathrm{U5M}_j} \; .$$

In all countries, U5M in the lower wealth quintiles are greater than in the higher wealth quintiles, therefore replacing the flat distribution with U5M would lead to greater differences in the distribution of cases and deaths across quintiles. On average, the proportion of unattributable cases/deaths are 27%/28% for measles, 10%/7% for pneumonia, and 5%/4% for diarrhea. The impact of this replacement should be greater on countries with higher proportion of unattributable cases/deaths.

In Tables S6.3 and S6.4 we highlight the results with significant differences in the change in AUC of greater than 25% for cases and deaths, respectively, compared to the main results (presented in Appendix V). We found that the results were mostly not sensitive to this adjustment. The results that had larger differences compared to the main results were for Scenario 5 of PCV and RV. This scenario assumes equal baseline mortality risk with current quintile-specific treatment coverage rates. In this sensitivity analysis, the change in the AUC were more favorable (more equal distribution of cases across quintiles) than the main results because countries started with more unequal case distributions, and therefore the lower quintiles benefited more when baseline mortality risk was equalized.

Table S6.1. Sensitivity analysis – change in the area under the curve (AUC) for cases, compared to Scenario 1, with total fertility rate adjustment. Highlighted cells represent greater than 25% difference compared to the main results in Appendix V.

Scenarios:

S2: current quintile-specific vaccine coverage rates;
S3: national average of vaccine coverage for all quintiles;
S4: vaccine coverage proportional to quintile-specific baseline mortality risks;
S5: equal baseline morbidity risk with current quintile-specific vaccine coverage rates.

Country	MCV S2	MCV S3	MCV S4	MCV S5	PCV S2	PCV S3	PCV S4	PCV S5	RV S2	RV S3	RV S4	RV S5
Armenia	-3.6 (-3.9, -3.2)	16.1 (14.4, 18.1)	-15.2 (-17.7, -12.6)	-14.1 (-15.1, -12.8)	-1.8 (-2.2, -1.3)	-1.6 (-2, -1)	-2.2 (-3.2, -1.1)	-15 (-17.9, -11.4)	-2.3 (-2.5, -1.9)	-1.3 (-2.3, -0.1)	-3.2 (-6.6, -0.1)	-19 (-20, -17.5)
Burundi	3.5 (3, 4)	13.4 (12, 15)	-15 (-18.1, -11.8)	-3.7 (-5.2, -2)	0.4 (0.2, 0.6)	0.4 (0.2, 0.6)	0.4 (-0.8, 1.5)	-2.3 (-4.2, -0.6)	0.1 (0, 0.1)	0.6 (0.1, 1.3)	-0.4 (-4, 2.7)	-4.6 (-6.3, -3.1)
Benin	6.7 (6.2, 7.1)	4.7 (4.4, 5.1)	-9.3 (-10.4, -8.3)	-5.4 (-6, -4.8)	-1.6 (-1.9, -1.3)	-1.6 (-1.9, -1.4)	-2.2 (-3, -1.5)	-14.8 (-15.4, -14.2)	NA	NA	NA	NA
Burkina Faso	5.6 (4.8, 6.5)	9 (7.9, 10.1)	-16.2 (-18.5, -14)	-7.1 (-8.4, -5.9)	-1.4 (-1.7, -1.1)	-1.3 (-1.6, -0.9)	-1.6 (-2.5, -0.7)	-13.3 (-14.1, -12.3)	-2.8 (-3.3, -2.4)	-2.4 (-3.1, -1.7)	-3.7 (-6.6, -1.2)	-16.5 (-17.3, -15.9)
Bangladesh	5.6 (4.6, 6.6)	11.7 (10.3, 13.3)	-19.6 (-22.4, -17)	-9.1 (-10.8, -7.5)	-1.6 (-1.7, -1.5)	-1.4 (-1.6, -1.2)	-1.9 (-2.8, -1)	-14.6 (-15.1, -14.3)	NA	NA	NA	NA
Cote d'Ivoire	2.7 (1.5, 3.8)	3.2 (2, 4.2)	-12.1 (-14.5, -10)	-12.1 (-14, -10.5)	-0.4 (-1, 0)	-0.7 (-1.2, -0.3)	-0.9 (-1.7, 0)	-13.7 (-15.3, -12.7)	-3.2 (-4.1, -2.5)	-3.1 (-3.9, -2.3)	-4.1 (-5.9, -2.5)	-20.3 (-21.9, -18.9)
Cameroon	5.2 (3.4, 7.1)	7.8 (5.8, 10)	-24.6 (-26.9, -22.2)	-11.9 (-14.5, -9)	-5 (-7.7, -2.6)	-5.3 (-7.9, -3)	-5.8 (-8.5, -3.3)	-24.6 (-29.9, -19.6)	-9.3 (-10.3, -8)	-8.9 (-10.1, -7.3)	-10.9 (-13, -8.8)	-33.2 (-34.7, -31.2)
DRC	3.3 (2.8, 3.9)	6.9 (6.1, 7.6)	-22.9 (-25.4, -20.6)	-9.5 (-10.6, -8.5)	-1.7 (-2.7, -0.7)	-1.8 (-2.8, -0.8)	-2.3 (-3.5, -1.1)	-13.8 (-16.5, -11.3)	NA	NA	NA	NA
Congo	7.9 (6.4, 9.4)	7.7 (6.1, 9.2)	-21.6 (-24.6, -18.5)	-7.8 (-10, -5.6)	-0.8 (-1.8, 0.1)	-1.1 (-2, -0.3)	-1.4 (-2.7, -0.1)	-14.4 (-16.7, -12.2)	-4.3 (-5.3, -3.1)	-4 (-5.2, -2.7)	-5.8 (-8.7, -3.4)	-23.1 (-25.1, -21.1)
Comoros	5.4 (4.4, 6.5)	8.4 (7.1, 9.8)	-21 (-23.2, -18.8)	-13 (-14.6, -11.4)	NA	NA	NA	NA	NA	NA	NA	NA
Ethiopia	3.8 (3.2, 4.4)	5.1 (4.5, 5.8)	-14.5 (-15.9, -13)	-6.6 (-7.6, -5.6)	-1.4 (-3.4, 0.6)	-1.6 (-3.5, 0.3)	-2.1 (-4.2, 0)	-16 (-19.8, -12)	-3.6 (-4.3, -2.8)	-3.4 (-4.3, -2.4)	-5.3 (-7.8, -3.1)	-20.2 (-21.4, -18.8)
Ghana	-0.8 (-1.8, 0)	8.2 (6.6, 9.9)	-15.2 (-18.8, -11.4)	-18.6 (-20.4, -17.1)	-0.3 (-2.6, 1.4)	-0.4 (-2.7, 1.3)	-0.4 (-3, 1.7)	-14.9 (-19.8, -11)	-3.7 (-4.6, -2.8)	-3.1 (-4.1, -1.9)	-4.2 (-7.5, -1.4)	-21.9 (-23.6, -20.1)
Guinea	1 (0.5, 1.5)	1.6 (1, 2.2)	-10 (-11, -9)	-13.7 (-14.5, -12.6)	NA	NA	NA	NA	NA	NA	NA	NA
Gambia	-9 (-10.1, -8)	0.7 (0.2, 1.1)	-8.5 (-13, -4.6)	-22.4 (-24.1, -20.8)	-0.5 (-0.8, -0.1)	-0.5 (-0.8, -0.1)	-0.3 (-1.2, 0.6)	-8.5 (-9.5, -7.4)	-0.5 (-0.7, -0.3)	-0.6 (-0.8, -0.3)	-0.5 (-3, 2)	-8.8 (-9.2, -8.3)
Honduras	-3.5 (-4, -3.1)	6.7 (5.7, 7.7)	-11.9 (-14.5, -9.3)	-18.8 (-19.9, -17.9)	3 (0.8, 4.1)	2.7 (0.6, 3.9)	3.3 (1, 4.9)	-6.5 (-11.9, -3.6)	-2 (-2.7, -1.3)	-1.5 (-2.4, -0.7)	-2.5 (-6, 0.4)	-18.2 (-19.7, -16.7)
Haiti	-2.4 (-2.8, -2.1)	-0.5 (-1, -0.2)	-4 (-5, -3.2)	-20.2 (-21, -19.6)	-0.9 (-1.9, 0)	-0.9 (-2, -0.1)	-0.9 (-2, 0)	-16.7 (-18.2, -15.5)	-5.5 (-6.3, -4.7)	-5.4 (-6.2, -4.6)	-6.4 (-8.7, -4)	-24.9 (-26.5, -23.5)
Indonesia	2.3 (2, 2.7)	10.3 (9.4, 11.2)	-15.6 (-18, -13.4)	-10.4 (-11.3, -9.5)	NA	NA	NA	NA	NA	NA	NA	NA
Kenya	1.4 (0.3, 2.2)	6.9 (5.5, 8.1)	-20.9 (-23.2, -18.6)	-18 (-19.6, -16.6)	-3.8 (-4.8, -3.1)	-3.6 (-4.6, -2.9)	-4.2 (-5.4, -3.1)	-22.5 (-24.3, -21.1)	-5.8 (-7.1, -5)	-5 (-6.5, -3.6)	-6.8 (-9.8, -4.3)	-26.3 (-28.6, -24.7)

Kyrgyz Republic	-4.4 (-5.1, -3.8)	6.4 (5.6, 7.4)	-3.3 (-6.5, -0.3)	-11.1 (-12.2, -10.1)	-0.6 (-1, -0.1)	-0.4 (-0.7, 0.1)	-0.4 (-1.2, 0.4)	-6.8 (-8.3, -5.3)	NA	NA	NA	NA
Cambodia	11.3 (9.9, 12.8)	10.7 (9.4, 12)	-16.4 (-19.2, -13.7)	-2.2 (-4, -0.3)	0.2 (-1, 1.5)	-0.1 (-1.3, 1.2)	-0.1 (-1.6, 1.7)	-9.1 (-13.4, -4.3)	NA	NA	NA	NA
Liberia	-0.9 (-1.2, -0.5)	1.1 (0.6, 1.5)	-9.4 (-10.3, -8.5)	-17.7 (-18.4, -17.1)	-0.6 (-2.2, 0.9)	-0.7 (-2.4, 0.8)	-0.9 (-2.8, 0.9)	-15.4 (-18.7, -12.3)	-3.1 (-3.4, -2.7)	-2.9 (-3.3, -2.5)	-3.6 (-5, -2.4)	-20.3 (-21, -19.5)
Lesotho	-1.4 (-2.8, -0.2)	8.2 (6, 10.3)	-18.7 (-21.9, -15.7)	-20.7 (-23.3, -18.6)	-2.4 (-4.4, -0.8)	-2.2 (-4.2, -0.6)	-2.6 (-4.8, -0.7)	-19.3 (-23.6, -15.8)	NA	NA	NA	NA
Mali	6.1 (5.4, 6.8)	11.2 (10.2, 12.3)	-22.5 (-24.7, -20.4)	-4.9 (-6.1, -3.8)	-1.1 (-1.6, -0.5)	-1.2 (-1.7, -0.6)	-1.8 (-2.7, -0.9)	-12.7 (-14.6, -10.5)	-1.8 (-2.3, -1.2)	-1.3 (-2.2, -0.1)	-3.4 (-5.8, -1.4)	-16 (-16.7, -15.1)
Mozambique	2 (1.4, 2.6)	8.3 (7.4, 9.3)	-24.1 (-26.1, -22.2)	-13.1 (-14.2, -12)	-4.3 (-5.1, -3.6)	-4.1 (-5, -3.3)	-4.9 (-6, -3.9)	-22.5 (-25, -20.1)	-5.3 (-5.6, -4.8)	-4.4 (-5.5, -3.2)	-6.8 (-9.2, -4.6)	-25.3 (-26.1, -24.4)
Malawi	-1.1 (-1.8, -0.2)	9.7 (8, 11.5)	-20.3 (-23, -17.4)	-16.3 (-18.1, -14)	-3.9 (-5.5, -2.3)	-3.6 (-5.3, -2)	-4.2 (-6, -2.3)	-21.1 (-25.1, -16.9)	-5.3 (-6, -4.5)	-4.5 (-5.5, -3.2)	-6.2 (-8.9, -3.8)	-24.4 (-26.3, -22.1)
Niger	7 (6.5, 7.6)	10.1 (9.4, 10.9)	-12.7 (-14.2, -11.3)	0 (-0.8, 0.7)	-0.1 (-0.3, 0.2)	-0.2 (-0.4, 0)	-0.8 (-1.6, -0.1)	-8.2 (-8.9, -7.3)	0.3 (-0.5, 1.2)	0.4 (-0.4, 1.5)	-1.6 (-4.7, 1)	-9.1 (-9.9, -8.1)
Nigeria	10 (8.8, 10.9)	1.1 (0.4, 1.7)	-10.6 (-12, -9.6)	-1.8 (-3.7, -0.4)	-2 (-2.7, -1.1)	-2.8 (-3.2, -2.3)	-3.3 (-4, -2.6)	-17.3 (-18.7, -16)	NA	NA	NA	NA
Nepal	-1.7 (-3.5, -0.1)	3.5 (1.3, 5.4)	-18.8 (-22.8, -15.1)	-18.7 (-21.7, -16.1)	0.1 (-1.9, 2)	0 (-2.1, 1.9)	0.1 (-2.2, 2.2)	-15 (-18.9, -11.5)	NA	NA	NA	NA
Pakistan	6.1 (5.1, 7.2)	4.5 (3.6, 5.5)	-20 (-22.5, -17.6)	-11.3 (-12.9, -9.7)	-3 (-3.9, -2.2)	-3.3 (-4.2, -2.6)	-4.1 (-5.3, -3.1)	-20.5 (-22.6, -18.7)	-1.4 (-3.4, 0.6)	-1.6 (-3.5, 0.3)	-2.1 (-4.2, 0)	-16 (-19.8, -12)
Rwanda	1.5 (0.9, 2.1)	10.4 (9, 12)	-14 (-17.1, -11)	-10.7 (-11.8, -9.6)	-0.2 (-0.8, 0.3)	-0.1 (-0.8, 0.3)	-0.2 (-1.4, 1)	-9.7 (-11.7, -8.1)	-1.7 (-2.1, -1.4)	-1 (-1.7, 0.1)	-2.3 (-5.8, 0.6)	-14.7 (-15.9, -13.7)
Senegal	-0.2 (-0.8, 0.5)	8.8 (7.6, 10)	-17.1 (-19.8, -13.4)	-14 (-15.7, -12.3)	-0.9 (-2, -0.1)	-0.8 (-1.9, -0.1)	-1.1 (-2.5, 0.1)	-12 (-15.3, -9.6)	-1.3 (-2, -0.3)	-0.8 (-1.7, 0.3)	-2 (-5.1, 0.9)	-13.6 (-15.8, -10.9)
Sierra Leone	-2.9 (-3.3, -2.6)	3.2 (2.5, 3.8)	-7.7 (-9.7, -5.9)	-15.3 (-16.2, -14.6)	-0.5 (-1.1, 0.1)	-0.5 (-1.1, 0.2)	-0.5 (-1.4, 0.5)	-11.4 (-12.6, -10.2)	-1.9 (-2.1, -1.6)	-1.5 (-1.9, -1)	-2.3 (-4.8, -0.1)	-14.8 (-15.6, -14.1)
Chad	2.1 (1.9, 2.3)	-0.2 (-0.3, 0)	-0.5 (-1.2, 0)	-2.4 (-3, -1.9)	NA	NA	NA	NA	NA	NA	NA	NA
Togo	-1 (-1.6, -0.4)	3.9 (3, 4.7)	-19.1 (-22, -16.4)	-13.8 (-14.9, -12.8)	-2.6 (-3, -2.3)	-2.5 (-2.9, -2.2)	-2.9 (-3.7, -2.1)	-17.3 (-18.3, -16.4)	-5.6 (-6.2, -5.2)	-5.1 (-5.9, -4.2)	-6.5 (-8.9, -4.1)	-24.3 (-25.4, -23.3)
Tajikistan	1.1 (0.9, 1.3)	7.5 (6.6, 8.5)	-4 (-7, -0.8)	-6.2 (-6.9, -5.5)	NA	NA	NA	NA	-0.3 (-0.5, -0.1)	0.2 (-0.2, 0.7)	-0.4 (-4.1, 2.8)	-7.4 (-8.3, -6.3)
Timor-Leste	7.9 (7.2, 8.5)	6.3 (5.7, 6.9)	-11.3 (-12.7, -9.8)	-4.9 (-5.8, -4)	NA	NA	NA	NA	NA	NA	NA	NA
Tanzania	1.3 (0.5, 2)	9.3 (7.9, 10.8)	-13.6 (-16.2, -11)	-13.8 (-14.9, -12.7)	0.6 (-0.6, 1.4)	0.5 (-0.7, 1.3)	0.5 (-0.9, 1.8)	-12.1 (-15.4, -9.7)	-2.1 (-2.6, -1.7)	-1.6 (-2.4, -0.7)	-2.8 (-6, -0.2)	-18.4 (-19.3, -17.6)
Uganda	0.6 (0.1, 1.1)	3.8 (3.2, 4.5)	-8.5 (-11.4, -5.9)	-10.2 (-11.2, -9.1)	-0.4 (-1.2, 0.4)	-0.3 (-1.2, 0.5)	-0.3 (-1.4, 0.8)	-10.3 (-12, -8.7)	-2.7 (-3.1, -2.4)	-2.6 (-2.9, -2.3)	-2.8 (-3.4, -2.2)	-16.2 (-17.3, -15.5)
Yemen	3.1 (2.2, 4)	7.4 (6.2, 8.6)	-22.8 (-24.6, -21)	-13.8 (-15.1, -12.5)	-2.8 (-3.7, -1.8)	-2.9 (-3.8, -1.9)	-3.5 (-4.7, -2.3)	-19.9 (-22, -17.6)	-3.8 (-4.5, -3)	-3.4 (-4.4, -2.3)	-5.3 (-8.5, -2.9)	-22.5 (-23.4, -21.4)
Zambia	2.5 (1.7, 3.2)	6.6 (5.5, 7.6)	-13.4 (-15.6, -11.4)	-13 (-14.1, -12)	-0.4 (-1.1, 0.2)	-0.5 (-1.1, 0.2)	-0.7 (-1.7, 0.3)	-16 (-17.5, -14.5)	-2.2 (-2.6, -1.8)	-1.8 (-2.5, -1)	-2.9 (-6, -0.5)	-19.4 (-20.2, -18.8)
Zimbabwe	2.1 (1.5, 2.6)	6.9 (6, 7.9)	-15.3 (-17.8, -13)	-10.6 (-11.5, -9.7)	-3.1 (-3.5, -2.8)	-3.1 (-3.5, -2.8)	-3.5 (-4.3, -2.6)	-18.4 (-19.2, -17.9)	-5 (-5.3, -4.7)	-4.5 (-5.2, -3.7)	-5.9 (-8.6, -3.4)	-23 (-23.8, -22.3)

Table S6.2. Sensitivity analysis – change in the area under the curve (AUC) for deaths, compared to Scenario 1, with total fertility rate adjustment. Highlighted cells represent greater than 25% difference compared to the main results in Appendix V.

Scenarios

S2: current quintile-specific treatment coverage rates;
S3: national average of treatment coverage for all quintiles;
S4: treatment coverage proportional to quintile-specific baseline mortality risks;
S5: equal baseline mortality risk with current quintile-specific treatment coverage rates.

Country	MCV S2	MCV S3	MCV S4	MCV S5	PCV S2	PCV S3	PCV S4	PCV S5	RV S2	RV S3	RV S4	RV S5
Armenia	2.1 (-0.1, 4.5)	-0.9 (-1.1, -0.7)	-2.7 (-6.9, 2)	-7.6 (-10, -4.9)	0.5 (-0.3, 1.3)	-2.3 (-2.6, -1.8)	-5.8 (-7.7, -3.9)	-14.7 (-16.6, -12)	2.1 (1.3, 3)	-2.3 (-2.6, -1.8)	-8.7 (-10.6, -6.5)	-13.1 (-15.4, -10)
Burundi	0.1 (-0.2, 0.6)	-0.3 (-0.4, -0.2)	-3.3 (-11.7, 5.8)	-7 (-8.6, -5.3)	1.1 (0.8, 1.4)	0.2 (0.1, 0.3)	-1.1 (-4, 2.2)	-2.3 (-4.7, -0.3)	0.5 (0.3, 0.7)	0.2 (0, 0.3)	-3.8 (-6.8, -0.7)	-3.7 (-5.1, -2.4)
Benin	0 (-1.3, 1.4)	-1.8 (-1.9, -1.7)	-3.2 (-7.1, 1.4)	-13.2 (-14.2, -12)	-0.1 (-0.6, 0.3)	-2 (-2.1, -1.9)	-3.7 (-4.8, -2.4)	-14.1 (-14.6, -13.5)	0.7 (0.2, 1.1)	-2.5 (-2.6, -2.3)	-6.5 (-7.6, -5.3)	-14.2 (-14.9, -13.7)
Burkina Faso	1.4 (-1.5, 4.9)	-2.4 (-2.7, -2.1)	-4.5 (-10.7, 2.5)	-11.5 (-15, -7.2)	3.1 (1.7, 4.6)	-1.8 (-2, -1.6)	-3.9 (-6.2, -1.4)	-8.6 (-10.2, -6.8)	2.7 (1.7, 3.5)	-2.7 (-3.1, -2.4)	-6.5 (-8.1, -4.8)	-9.7 (-11.1, -8.5)
Bangladesh	0.6 (-1.7, 3.1)	-2.5 (-3, -2.1)	-4 (-7.7, 0.1)	-13.5 (-16.7, -10.1)	2.4 (1.3, 3.6)	-1.8 (-2, -1.7)	-3.5 (-5, -1.9)	-10.5 (-11.8, -9.1)	1.7 (1.1, 2.3)	-2.4 (-2.6, -2.2)	-4.6 (-5.3, -3.8)	-11.8 (-12.9, -10.9)
Cote d'Ivoire	-1.3 (-3.1, 0.6)	-3.5 (-4.3, -2.9)	-5 (-8.5, -1)	-18.5 (-20.8, -16.2)	2.8 (1.7, 4)	-1.3 (-1.8, -0.9)	-2.2 (-3.8, -0.6)	-11.7 (-13.3, -10.2)	-1.2 (-2.1, -0.5)	-2.8 (-3.6, -2.2)	-4.6 (-6, -3.4)	-17.5 (-19.5, -16)
Cameroon	-4 (-6, -1.7)	-6.4 (-7.5, -5.3)	-8.7 (-12, -4.9)	-24.8 (-27.5, -21.6)	-4.1 (-6.2, -2)	-7.2 (-9, -5.3)	-10.4 (-13.4, -7.3)	-26.1 (-29.8, -22.1)	-5 (-7.5, -2.8)	-8 (-10.1, -6.1)	-12.1 (-15.2, -9.2)	-26.4 (-30.6, -22.4)
DRC	-3.3 (-3.6, -3.1)	-3.5 (-3.7, -3.4)	-6 (-10.1, -1.1)	-19.2 (-20, -18.6)	-2 (-2.5, -1.3)	-2.8 (-3.3, -2.2)	-5.1 (-6.7, -3.3)	-15.7 (-17.1, -14)	-3.4 (-3.9, -2.9)	-2.7 (-3.4, -2.1)	-6.5 (-8, -5)	-18 (-19.8, -16.2)
Congo	-1.3 (-3.3, 1)	-3.8 (-4.6, -3.1)	-6.4 (-12, -0.2)	-17.9 (-21.1, -14.5)	1.8 (-0.1, 3.6)	-2.5 (-3.6, -1.3)	-5 (-8, -1.7)	-12.9 (-16.1, -9.6)	-1.2 (-2.7, 0.1)	-3.9 (-5.1, -3)	-7.8 (-10, -5.6)	-17.5 (-20.3, -15)
Comoros	-4.2 (-4.8, -3.6)	-4.1 (-4.6, -3.4)	-6.3 (-11.3, -1)	-22.3 (-23.6, -20.8)	-3.5 (-4.5, -2.2)	-4.2 (-5.2, -2.9)	-7 (-9.4, -4.6)	-21.5 (-23.7, -18.5)	-4.1 (-4.9, -3)	-3.7 (-4.6, -2.5)	-7.7 (-9.9, -5.2)	-21.4 (-23.6, -18.8)
Ethiopia	-1 (-2.9, 1.1)	-3.7 (-3.9, -3.3)	-5.1 (-8.1, -1.4)	-15.3 (-18.3, -11.8)	0.3 (-1.1, 2.3)	-3.1 (-4.1, -1.8)	-4.7 (-6.3, -2.9)	-13.6 (-16.1, -10.6)	1.1 (-0.5, 2.8)	-3.1 (-4.3, -1.9)	-6.1 (-7.9, -4)	-12.9 (-15.4, -10.2)
Ghana	-5.5 (-7.2, -4)	-3.8 (-4.6, -3.1)	-5.9 (-11.5, 0.3)	-23.2 (-25, -21.4)	-2.8 (-4.5, -1.1)	-1.9 (-3.7, -0.3)	-3.4 (-7.2, 0.4)	-18.8 (-22.4, -15.4)	-5.5 (-7, -4)	-2.3 (-3.8, -0.8)	-4.9 (-8.2, -1.7)	-21.2 (-24.2, -18.3)
Guinea	0 (-2.9, 3.3)	-4 (-4.3, -3.6)	-6 (-10.2, -0.9)	-14.4 (-18.5, -9.6)	2 (0.1, 3.9)	-3.9 (-4.7, -2.9)	-6.6 (-8.7, -4.4)	-11.9 (-14.9, -8.7)	0.2 (-1.1, 1.6)	-4.4 (-5.3, -3.4)	-9.2 (-11.3, -6.9)	-15.2 (-17.6, -12.6)
Gambia	-2 (-3, -1.4)	-1.3 (-1.6, -1)	-2.1 (-10.9, 7.6)	-12.7 (-14.3, -11.5)	-1.8 (-2.3, -1.4)	-0.7 (-0.9, -0.5)	-0.1 (-2.8, 3.2)	-10.5 (-11.2, -9.8)	-1.4 (-1.8, -1)	-0.3 (-0.6, 0)	1.9 (-0.9, 5.1)	-9.4 (-10.2, -8.5)
Honduras	-1 (-1.9, 0)	-1.9 (-2.3, -1.4)	-3.5 (-11.8, 5.8)	-17 (-18.4, -15.6)	3.6 (1.5, 5.3)	1.5 (-0.3, 2.9)	3.6 (-1.3, 8.1)	-7.7 (-12.4, -4.2)	0.3 (-1.9, 1.9)	0.3 (-2, 1.8)	0.7 (-5.1, 5)	-13 (-18, -9.3)
Haiti	0.5 (-1.5, 2.7)	-2.2 (-2.6, -1.8)	-2.7 (-6.2, 1.7)	-16.3 (-18.9, -13.4)	1.8 (0.6, 3)	-2 (-2.7, -1.3)	-2 (-3.4, -0.4)	-14.1 (-15.6, -12.6)	-1.2 (-3, 0.2)	-4.2 (-5.8, -3)	-5.9 (-8.1, -3.9)	-18.3 (-20.9, -16.2)
Indonesia	-0.2 (-1.4, 1.9)	-1.7 (-1.9, -1.5)	-6.5 (-19.7, 7)	-13.3 (-14.4, -11.8)	2.1 (1, 3.6)	-1.1 (-1.3, -0.9)	-5.3 (-10.6, 0.3)	-9.4 (-10.6, -7.9)	-1.6 (-1.9, -1.3)	-1.5 (-1.8, -1.1)	-10.1 (-14.4, -5.6)	-13.5 (-15, -12.1)
Kenya	-4.9 (-5.7, -4.2)	-4.9 (-5.7, -4.2)	-9.2 (-19.7, 0.8)	-24.3 (-25.8, -23.1)	-3.5 (-4.6, -2.6)	-4.5 (-5.6, -3.7)	-9.2 (-13.5, -5.3)	-22.3 (-24.5, -20.8)	-7.3 (-8.4, -6.5)	-5.6 (-6.7, -4.8)	-15.4 (-20.2, -11.5)	-27.3 (-29.3, -25.9)

Kyrgyz Republic	3.9 (0.8, 7.9)	-0.1 (-0.3, 0.2)	-0.6 (-7.1, 7)	-1.9 (-5.8, 3.3)	3.9 (2.5, 5.3)	-0.3 (-0.8, 0.2)	-0.9 (-3.3, 1.7)	-1.7 (-4.2, 1)	5.9 (4.6, 7.1)	-0.9 (-1.2, -0.6)	-3.4 (-5.6, -1.2)	0.4 (-1.8, 2.6)
Cambodia	-3.2 (-4.4, -2.4)	-2.2 (-2.6, -2)	-5.8 (-15.6, 4.7)	-17.9 (-19.6, -16.6)	-0.8 (-1.3, -0.1)	-0.9 (-1.5, -0.2)	-3 (-7.3, 1.8)	-12.5 (-14.5, -9.8)	-5.8 (-6.6, -4.8)	-1.1 (-1.9, -0.3)	-4.4 (-8.5, -0.2)	-17.4 (-20.2, -14.5)
Liberia	-3.7 (-4, -3.4)	-3.8 (-4.1, -3.5)	-5.8 (-11.2, 0.3)	-21.7 (-22.4, -21.1)	-2.6 (-3.4, -1.6)	-2 (-2.9, -1)	-3.5 (-6, -0.9)	-19.3 (-21.2, -17.2)	-0.9 (-2, 0.1)	-2.2 (-3.2, -1.2)	-5 (-7.6, -2.4)	-17.5 (-19.4, -15.5)
Lesotho	-5.1 (-6.5, -4)	-4.5 (-5.8, -3.5)	-8 (-16, 0.6)	-23.7 (-26.2, -21.5)	-3.1 (-5.4, -1.2)	-3.5 (-5.8, -1.6)	-7.5 (-13, -2.5)	-21.3 (-26, -17.2)	-8.9 (-10.9, -7.3)	-6.2 (-8.2, -4.5)	-15.2 (-20.6, -10.7)	-28.4 (-32.1, -25.3)
Mali	0.7 (-1.5, 3)	-2.2 (-2.5, -2.1)	-4 (-7.3, -0.3)	-12.1 (-14.8, -9.1)	2.4 (1.3, 3.5)	-1.8 (-2.1, -1.5)	-3.8 (-5, -2.4)	-9.4 (-11.1, -7.5)	1.8 (1.1, 2.4)	-2 (-2.4, -1.7)	-5.2 (-6.1, -4.1)	-10.8 (-12.2, -9.3)
Mozambique	-3.1 (-4.7, -0.9)	-5.2 (-5.3, -5.1)	-10.2 (-18.3, -1.8)	-21.2 (-23.4, -18.1)	-2.8 (-3.6, -1.9)	-5.2 (-5.7, -4.7)	-10.9 (-13.9, -8.1)	-21.3 (-23.1, -19.2)	-1.3 (-2.3, -0.4)	-5.1 (-5.7, -4.6)	-17.9 (-22.6, -13.5)	-19.3 (-21.2, -17.4)
Malawi	-2.6 (-3.5, -1.3)	-3.4 (-4.1, -2.7)	-8.2 (-18.4, 2.5)	-18.4 (-20.4, -16)	-2.4 (-3.9, -0.7)	-4.7 (-5.8, -3.2)	-12.9 (-18.4, -7.4)	-20.4 (-23.6, -16.1)	-5.5 (-6.5, -4.4)	-4.6 (-5.6, -3.4)	-19.6 (-25.3, -14)	-24 (-26.5, -21.2)
Niger	3.3 (0.2, 7.2)	-0.7 (-0.8, -0.6)	-3.8 (-11.5, 4.7)	-4.7 (-8.1, -0.1)	6.4 (4.4, 8.6)	-0.5 (-0.6, -0.4)	-4.2 (-7, -1.4)	-1.3 (-3.6, 1.1)	2.8 (2.2, 3.4)	-0.5 (-0.6, -0.5)	-7.6 (-9.7, -5.4)	-6.4 (-7.1, -5.7)
Nigeria	-1.3 (-3.3, 0.8)	-3.9 (-4.4, -3.6)	-5.9 (-9.6, -1.8)	-17.7 (-20.4, -14.7)	0.1 (-0.8, 1)	-3.4 (-3.6, -3.1)	-5.6 (-7.1, -4.1)	-14.8 (-16.1, -13.4)	0.4 (-0.2, 0.9)	-3.3 (-3.6, -2.9)	-5.9 (-6.7, -4.9)	-15.2 (-16.1, -14.2)
Nepal	-4.2 (-5.9, -2.7)	-5.2 (-6.6, -3.9)	-6.7 (-10.8, -1.8)	-24 (-26.8, -21.6)	0.4 (-1.3, 2)	-1.5 (-3, 0)	-1.8 (-4.2, 0.9)	-16.1 (-19.3, -13.1)	-1.7 (-3.1, -0.4)	-3.3 (-4.7, -2.2)	-5.2 (-7.4, -3.2)	-20.2 (-22.6, -18)
Pakistan	-2 (-4.4, 1.6)	-5 (-5.5, -4.5)	-12.3 (-25.7, 0)	-20.2 (-23.7, -15.1)	-0.7 (-1.9, 0.5)	-4.1 (-4.6, -3.6)	-11.4 (-16, -6.9)	-18 (-19.8, -16.1)	1.4 (0, 2.7)	-4.5 (-5, -4.1)	-19.2 (-24, -14.7)	-15 (-17.2, -13)
Rwanda	1.1 (-1.2, 4.3)	-1.8 (-2.1, -1.6)	-4.7 (-12.9, 4.3)	-11.1 (-13.9, -7.2)	3.3 (1.9, 5)	-0.9 (-1.3, -0.5)	-3.8 (-8, 0.5)	-7.4 (-9.5, -5.1)	3.8 (2.8, 4.8)	-1.4 (-1.8, -1)	-6.5 (-9, -3.9)	-8.7 (-10.4, -7.2)
Senegal	-2.8 (-3.4, -2.3)	-2.5 (-3, -2)	-4.2 (-8.2, 0.6)	-17.3 (-18.9, -15.7)	-2.7 (-3.6, -1.8)	-1.7 (-2.6, -0.8)	-3.1 (-5.3, -0.7)	-15.9 (-18.6, -12.9)	-0.5 (-1.4, 0.3)	-0.9 (-1.7, -0.1)	-1.9 (-3.6, -0.2)	-11.1 (-13.6, -8.7)
Sierra Leone	-0.8 (-1.8, 0.9)	-2 (-2.3, -1.7)	-3.6 (-11.9, 6.2)	-13.3 (-14.8, -11.4)	-0.1 (-0.6, 0.5)	-1 (-1.4, -0.5)	-2.1 (-5.4, 1.8)	-11.2 (-12.3, -10)	1.7 (0.9, 2.5)	-1.4 (-1.9, -1)	-5.3 (-8.2, -1.9)	-10.7 (-11.9, -9.5)
Chad	2.3 (0.3, 4.4)	-0.4 (-0.5, -0.3)	-0.4 (-2.9, 2.5)	-2.5 (-4.9, -0.1)	3.4 (2.5, 4.3)	-0.1 (-0.3, 0)	0.1 (-0.7, 1.2)	0.2 (-0.9, 1.2)	3.3 (2.8, 3.8)	-0.5 (-0.7, -0.4)	-0.7 (-1.3, 0)	-1.7 (-2.7, -0.8)
Togo	-2.8 (-3.6, -1.9)	-3.7 (-4.1, -3.3)	-5.2 (-9.2, -0.4)	-17.8 (-19.5, -16)	-1.4 (-2, -0.8)	-3.2 (-3.4, -3)	-5.3 (-7.3, -3.3)	-15.5 (-16.6, -14.5)	-5.1 (-5.9, -4.5)	-5.2 (-5.8, -4.7)	-8 (-9.1, -7)	-21.7 (-23.2, -20.5)
Tajikistan	1.7 (0.3, 3.6)	-0.1 (-0.2, -0.1)	-0.7 (-8.5, 8)	-4.7 (-6, -2.9)	2.9 (2.1, 3.9)	0 (-0.2, 0.3)	-0.8 (-3.8, 2.6)	-3.4 (-5, -1.3)	3.6 (2.9, 4.2)	0.1 (-0.2, 0.3)	-1.3 (-4.1, 2)	-2.6 (-4.4, -0.6)
Timor-Leste	-0.3 (-1.7, 2.2)	-2 (-2.2, -1.8)	-5.7 (-17.9, 6.9)	-13.3 (-14.8, -10.6)	1.3 (0.2, 2.6)	-1.8 (-2.2, -1.2)	-5.4 (-9.7, -1)	-11 (-12.7, -9)	2.2 (1.1, 3.3)	-1.8 (-2.2, -1.3)	-10.9 (-15.7, -5.2)	-10.1 (-11.8, -8.3)
Tanzania	-2.5 (-3.2, -1.7)	-3 (-3.5, -2.6)	-5.6 (-12.1, 1.8)	-18.3 (-19.7, -16.7)	1.2 (0.3, 2.1)	-0.6 (-1.3, 0)	-2.3 (-5.4, 0.8)	-12.4 (-14.3, -10.5)	0.2 (-1, 1.1)	-1.2 (-2.2, -0.4)	-4.5 (-8.2, -1.2)	-13.3 (-15.9, -11.1)
Uganda	-2.1 (-2.6, -1.7)	-1.8 (-2.2, -1.5)	-3.8 (-15.1, 9.1)	-14.4 (-15.5, -13.4)	-0.9 (-1.4, -0.2)	-1.1 (-1.7, -0.5)	-2.1 (-6.6, 2.7)	-11.3 (-12.4, -10.1)	-3.6 (-4.3, -2.8)	-1.8 (-2.7, -1.1)	-8 (-12.8, -3.5)	-17.1 (-18.9, -15.4)
Yemen	-4.4 (-5.2, -3.5)	-5.3 (-5.8, -4.8)	-7.6 (-11.5, -3.2)	-23.5 (-24.9, -22.1)	-2.9 (-3.4, -2.2)	-3.9 (-4.4, -3.3)	-6.2 (-7.7, -4.4)	-20.6 (-21.9, -19.1)	-2.3 (-2.9, -1.6)	-3.9 (-4.5, -3.3)	-7.5 (-8.9, -6)	-20 (-21.4, -18.4)
Zambia	-1.7 (-2.8, -0.3)	-2.8 (-3.3, -2.4)	-6 (-16.6, 5.3)	-18.8 (-20.1, -17)	0.8 (0, 1.7)	-1.2 (-1.6, -0.9)	-4.1 (-8, 0)	-14.7 (-16, -13.3)	-1.3 (-1.8, -0.8)	-1.8 (-2.4, -1.4)	-8.3 (-12, -4.5)	-18.9 (-20.1, -17.8)
Zimbabwe	-3 (-3.3, -2.7)	-2.7 (-2.9, -2.5)	-4.1 (-8.3, 1.1)	-18 (-18.5, -17.5)	-4.5 (-4.9, -4.2)	-3.6 (-3.9, -3.3)	-5.8 (-7.6, -3.7)	-21 (-21.9, -20.2)	-4.7 (-5.3, -4.2)	-4.7 (-5.3, -4.2)	-8.2 (-9.5, -6.7)	-21.3 (-22.8, -20.3)

N/A: estimates not available due to no vaccine introduction in 2016.

Table S6.3. Sensitivity analysis – change in the area under the curve for cases, compared to Scenario 1, with under-five mortality rate adjustment. Highlighted cells represent greater than 25% difference compared to the main results in Appendix V.

Scenarios:

S2: current quintile-specific vaccine coverage rates;
S3: national average of vaccine coverage for all quintiles;
S4: vaccine coverage proportional to quintile-specific baseline mortality risks;
S5: equal baseline morbidity risk with current quintile-specific vaccine coverage rates.

Country	MCV S2	MCV S3	MCV S4	MCV S5	PCV S2	PCV S3	PCV S4	PCV S5	RV S2	RV S3	RV S4	RV S5
Armenia	-2.9 (-3.3, -2.5)	16.6 (14.9, 18.4)	-15.5 (-18.1, -12.7)	-15.1 (-16, -14)	0.1 (0, 0.2)	0.3 (0.1, 0.6)	-0.3 (-1.2, 0.5)	-13.5 (-16.3, -10.1)	0.1 (0, 0.3)	1.1 (0.1, 2.4)	-0.9 (-4.3, 2.1)	-16.6 (-17.6, -15.1)
Burundi	3.8 (3.4, 4.3)	13.8 (12.4, 15.4)	-14.9 (-18, -11.8)	-4.6 (-6.2, -2.9)	0 (0, 0)	0.1 (0, 0.1)	0 (-1.1, 1.2)	-2.1 (-4, -0.4)	0 (0, 0.1)	0.6 (0.1, 1.3)	-0.5 (-4.3, 2.7)	-4 (-5.8, -2.5)
Benin	9.4 (9, 10)	7.3 (6.9, 7.7)	-8 (-9, -7)	1.7 (1.1, 2.3)	0.4 (0.1, 0.6)	0.3 (0.1, 0.6)	-0.3 (-1.2, 0.5)	-7.1 (-7.6, -6.6)	NA	NA	NA	NA
Burkina Faso	9.3 (8.4, 10.3)	14.1 (12.9, 15.5)	-17 (-19.4, -14.8)	1 (-0.4, 2.2)	0.1 (0, 0.1)	0.2 (0.1, 0.4)	-0.2 (-1.2, 0.8)	-4.8 (-5.7, -3.9)	0.2 (0, 0.5)	0.7 (0.1, 1.6)	-1 (-4.3, 1.7)	-7.7 (-8.6, -7.1)
Bangladesh	8.7 (7.9, 9.7)	16.0 (14.6, 17.6)	-18.5 (-21.2, -16.1)	-0.6 (-2.2, 1)	0.1 (0, 0.2)	0.3 (0.1, 0.5)	-0.2 (-1.2, 0.8)	-5.9 (-6.4, -5.5)	NA	NA	NA	NA
Cote d'Ivoire	8.6 (8, 9.2)	8.9 (8.3, 9.4)	-10.1 (-12.2, -8.3)	0.2 (-1.6, 1.8)	0.5 (0.2, 0.8)	0.1 (0, 0.2)	-0.1 (-0.9, 0.8)	-2.1 (-3.5, -1.2)	0.5 (0.1, 0.9)	0.6 (0.1, 1.3)	-0.7 (-2.7, 1)	-8 (-9.6, -6.6)
Cameroon	15 (13.8, 16.3)	18.6 (17.1, 20.3)	-21.6 (-23.7, -19.6)	-0.2 (-2.7, 2.4)	0.7 (0.3, 1.2)	0.3 (0.1, 0.5)	-0.4 (-1.3, 0.3)	-12.3 (-17.2, -7.7)	1 (0.1, 2)	1.5 (0.2, 3.2)	-1.5 (-4.3, 1)	-19.8 (-21.4, -17.8)
DRC	8 (7.5, 8.6)	12.6 (11.8, 13.4)	-22 (-24.5, -19.7)	-2.6 (-3.7, -1.6)	0.5 (0.2, 0.8)	0.4 (0.1, 0.6)	-0.4 (-1.2, 0.5)	-7.1 (-9.7, -4.5)	NA	NA	NA	NA
Congo	14.2 (13.1, 15.4)	14.4 (13.2, 15.7)	-19.6 (-22.4, -16.7)	4.4 (2.2, 6.4)	0.5 (0.2, 0.8)	0.2 (0.1, 0.3)	-0.2 (-1.1, 0.8)	-3.5 (-5.5, -1.5)	0.8 (0.1, 1.7)	1.1 (0.1, 2.4)	-1.2 (-4.4, 1.4)	-10.8 (-12.8, -8.9)
Comoros	11.1 (10.3, 11.9)	15.5 (14.3, 16.7)	-17.9 (-19.8, -16)	1.4 (-0.1, 2.9)	NA	NA	NA	NA	NA	NA	NA	NA
Ethiopia	11.5 (10.9, 12.3)	12.7 (11.9, 13.4)	-13.8 (-15.2, -12.3)	3.8 (2.7, 4.8)	0.6 (0.3, 1.1)	0.3 (0.1, 0.5)	-0.3 (-1.2, 0.6)	-5.9 (-9.8, -1.9)	1 (0.1, 2)	1.2 (0.1, 2.5)	-1.3 (-4.3, 1.1)	-9.9 (-11.2, -8.4)
Ghana	4.1 (3.6, 4.6)	15.5 (14, 17.2)	-14 (-17.7, -10)	-4.9 (-6.4, -3.5)	0.1 (0, 0.1)	0 (-0.1, 0)	0 (-1.1, 1.3)	-0.9 (-5, 2.2)	0.1 (0, 0.2)	0.8 (0.1, 1.8)	-0.6 (-4.6, 2.7)	-6.8 (-8.4, -5.2)
Guinea	5.8 (5.5, 6.1)	7 (6.6, 7.3)	-7.1 (-7.9, -6.4)	-5.9 (-6.7, -5)	NA	NA	NA	NA	NA	NA	NA	NA
Gambia	-9.8 (-11.2, -8.6)	3.4 (3, 3.8)	-9.2 (-13.8, -5.2)	-16.3 (-18.1, -14.7)	-0.1 (-0.1, 0)	0 (0, 0)	0.1 (-0.8, 1.1)	-0.2 (-1.1, 0.8)	0 (0, 0)	0 (0, 0.1)	0 (-3.1, 2.9)	-0.2 (-0.6, 0.2)
Honduras	-1.6 (-1.7, -1.4)	9.7 (8.8, 10.7)	-10.6 (-13.1, -8.1)	-10.8 (-11.6, -10.2)	0 (0, 0.1)	-0.3 (-0.4, -0.1)	0.3 (-0.6, 1.3)	2.8 (-1, 4.7)	0 (0, 0.1)	0.6 (0.1, 1.2)	-0.6 (-4.4, 2.5)	-5.2 (-6.4, -4)
Haiti	-0.4 (-0.5, -0.4)	2.4 (2.3, 2.5)	-2.5 (-3.2, -1.8)	-7.6 (-8.2, -7.1)	0.1 (0, 0.1)	0 (0, 0)	0 (-0.3, 0.3)	-1.7 (-3.4, -0.5)	0.5 (0.1, 1)	0.7 (0.1, 1.5)	-0.9 (-3.7, 1.6)	-8.7 (-10, -7.4)
Indonesia	4.1 (3.8, 4.4)	12.3 (11.4, 13.2)	-14.1 (-16.3, -12.1)	-7.7 (-8.5, -6.9)	NA	NA	NA	NA	NA	NA	NA	NA
Kenya	7.5 (7, 8.1)	16.2 (15, 17.5)	-18.2 (-20.1, -16.3)	-1.7 (-3.1, -0.6)	0.1 (0, 0.2)	0.4 (0.2, 0.7)	-0.4 (-1.3, 0.5)	-7.4 (-8.6, -6.4)	0.1 (0, 0.2)	1.2 (0.1, 2.6)	-1.1 (-4.5, 1.6)	-10.2 (-11.9, -9)

Kyrgyz Republic	-4.3 (-4.9, -3.7)	6.9 (6.1, 7.9)	-3.3 (-6.7, 0.1)	-8.3 (-9.3, -7.3)	-0.2 (-0.4, -0.1)	0 (0, 0.1)	0 (-0.8, 0.8)	-2.6 (-4.1, -1)	NA	NA	NA	NA
Cambodia	14.5 (13.1, 16)	13.7 (12.5, 15.1)	-14.9 (-17.6, -12.3)	3.9 (2.1, 5.8)	0.3 (0.1, 0.5)	-0.1 (-0.1, 0)	0 (-1.1, 1.2)	-1.3 (-5.4, 3.2)	NA	NA	NA	NA
Liberia	4.1 (3.9, 4.3)	7.3 (6.9, 7.7)	-7.5 (-8.4, -6.7)	-4 (-4.6, -3.5)	0.3 (0.1, 0.5)	0.1 (0, 0.1)	-0.1 (-0.9, 0.8)	-1.8 (-5, 1.1)	0.2 (0, 0.5)	0.5 (0.1, 1)	-0.5 (-2.3, 1)	-6.4 (-7, -5.7)
Lesotho	3.6 (3.2, 4)	17.4 (15.6, 19.3)	-18.2 (-20.9, -15.5)	-5.3 (-7.8, -3.4)	0 (0, 0)	0.3 (0.1, 0.5)	-0.3 (-1.2, 0.8)	-4.6 (-8.6, -1.5)	NA	NA	NA	NA
Mali	9.4 (8.7, 10.2)	15.3 (14.2, 16.4)	-22 (-24.3, -19.9)	0.1 (-1.1, 1.2)	0.5 (0.2, 0.8)	0.4 (0.2, 0.7)	-0.4 (-1.2, 0.4)	-7.3 (-9.1, -5.2)	0.6 (0.1, 1.3)	1.3 (0.2, 2.7)	-1.2 (-3.9, 1)	-10.3 (-11.1, -9.4)
Mozambique	9.9 (9.1, 10.8)	17.9 (16.6, 19.2)	-23.6 (-25.8, -21.6)	-2.4 (-3.5, -1.1)	0.3 (0.1, 0.5)	0.6 (0.2, 0.9)	-0.5 (-1.4, 0.2)	-12.3 (-14.8, -9.9)	0.4 (0.1, 0.9)	1.5 (0.2, 3.2)	-1.5 (-4.5, 0.9)	-14.9 (-15.7, -13.9)
Malawi	3 (2.7, 3.3)	17 (15.4, 18.7)	-20.6 (-23.2, -17.8)	-7.1 (-8.8, -5)	0 (0, 0.1)	0.4 (0.2, 0.7)	-0.4 (-1.3, 0.4)	-11.5 (-15.4, -7.4)	0 (0, 0.1)	1.1 (0.1, 2.3)	-1.2 (-4.2, 1.4)	-14.7 (-16.6, -12.4)
Niger	8.4 (7.8, 8.9)	11.7 (11, 12.5)	-12.7 (-14.2, -11.3)	1.9 (1.2, 2.6)	0.5 (0.2, 0.8)	0.3 (0.1, 0.6)	-0.3 (-1.1, 0.4)	-6.3 (-7.1, -5.5)	0.9 (0.1, 1.9)	1 (0.1, 2.2)	-1.1 (-4.4, 1.6)	-7.3 (-8.1, -6.2)
Nigeria	17.1 (16.2, 17.9)	6.2 (5.9, 6.4)	-7.8 (-8.8, -6.9)	7.5 (5.9, 8.9)	1.3 (0.5, 2.1)	0.3 (0.1, 0.5)	-0.3 (-1.1, 0.3)	-7.6 (-9, -6.3)	NA	NA	NA	NA
Nepal	5.6 (5, 6.3)	12.2 (11.1, 13.4)	-18.7 (-22, -15.4)	-3.3 (-5.8, -1)	0.2 (0.1, 0.3)	0 (-0.1, 0)	0 (-0.9, 1.2)	-0.4 (-4, 2.9)	NA	NA	NA	NA
Pakistan	13.5 (12.8, 14.3)	11.6 (11, 12.3)	-16.4 (-18.5, -14.2)	0.4 (-1.1, 1.9)	0.8 (0.3, 1.4)	0.4 (0.2, 0.7)	-0.5 (-1.4, 0.4)	-9 (-10.9, -7.4)	NA	NA	NA	NA
Rwanda	3.7 (3.2, 4.3)	13.5 (12.1, 15.2)	-13.5 (-16.7, -10.4)	-5 (-6.1, -3.9)	0 (0, 0.1)	0.1 (0, 0.1)	0 (-1.2, 1.3)	-1.8 (-3.9, -0.2)	0.1 (0, 0.1)	0.9 (0.1, 2)	-0.6 (-4.5, 2.7)	-6.7 (-8, -5.6)
Senegal	2.7 (2.5, 2.9)	13.1 (12.2, 14.3)	-16.4 (-19, -13)	-9.4 (-10.9, -7.9)	0.1 (0, 0.2)	0.2 (0.1, 0.3)	-0.1 (-1.1, 0.9)	-4.6 (-7.6, -2.3)	0.2 (0, 0.5)	0.7 (0.1, 1.6)	-0.7 (-4.4, 2.3)	-5.4 (-7.4, -2.8)
Sierra Leone	-0.8 (-0.9, -0.8)	8.2 (7.6, 8.9)	-8.3 (-10.2, -6.4)	-5.9 (-6.7, -5.2)	-0.1 (-0.1, 0)	0.1 (0, 0.1)	0 (-0.9, 1)	-1.3 (-2.5, 0)	0 (0, 0)	0.6 (0.1, 1.2)	-0.6 (-3.6, 2.1)	-4.4 (-5.2, -3.8)
Chad	3 (2.8, 3.1)	0.6 (0.6, 0.7)	-0.5 (-1.1, 0.1)	2 (1.4, 2.5)	NA	NA	NA	NA	NA	NA	NA	NA
Togo	3.8 (3.5, 4.2)	9.3 (8.6, 10.1)	-17.7 (-20.5, -15)	-5.8 (-6.8, -4.9)	0.1 (0, 0.1)	0.2 (0.1, 0.4)	-0.3 (-1.2, 0.5)	-7.7 (-8.8, -6.7)	0.1 (0, 0.3)	0.8 (0.1, 1.8)	-1.1 (-4, 1.4)	-13.8 (-14.8, -12.9)
Tajikistan	1.3 (1.1, 1.5)	8 (7.1, 9.1)	-3.9 (-7.1, -0.6)	-4.3 (-4.9, -3.6)	NA	NA	NA	NA	-0.1 (-0.3, 0)	0.4 (0, 0.9)	-0.3 (-4.1, 3.2)	-3.1 (-3.9, -2)
Timor-Leste	10.9 (10.3, 11.7)	10.2 (9.5, 10.8)	-10.4 (-11.8, -9)	4 (3, 4.8)	-1.4 (-3.4, 0.6)	-1.6 (-3.5, 0.3)	-2.1 (-4.2, 0)	-16 (-19.8, -12)	NA	NA	NA	NA
Tanzania	6.3 (5.5, 7.2)	16 (14.3, 17.9)	-14.4 (-17.3, -11.7)	-1.3 (-2.5, -0.1)	0.1 (0.1, 0.2)	0 (0, 0)	0.1 (-1.1, 1.3)	-0.1 (-3.1, 2.2)	0.2 (0, 0.5)	0.8 (0.1, 1.8)	-0.6 (-4.5, 2.5)	-5.9 (-6.7, -5.1)
Uganda	3.5 (3.2, 3.8)	8 (7.4, 8.7)	-8.7 (-11.5, -6.3)	-2.6 (-3.6, -1.6)	0 (0, 0)	0 (0, 0)	0 (-0.8, 0.8)	-1.5 (-3.2, 0.2)	0 (0, 0)	0.2 (0, 0.3)	-0.1 (-0.6, 0.3)	-6.8 (-7.7, -6.1)
Yemen	11.6 (10.7, 12.4)	17.7 (16.4, 19)	-20.4 (-22.2, -18.7)	0.4 (-1, 1.7)	0.5 (0.2, 0.9)	0.4 (0.2, 0.7)	-0.3 (-1.3, 0.7)	-7.3 (-9.1, -5.3)	0.9 (0.1, 1.9)	1.3 (0.2, 2.8)	-1.1 (-4.7, 1.8)	-9.1 (-10, -8)
Zambia	7.6 (6.9, 8.3)	12.7 (11.6, 13.9)	-13.6 (-15.9, -11.5)	0 (-1.1, 1)	0.2 (0.1, 0.4)	0.1 (0.1, 0.2)	-0.1 (-1.2, 1)	-2.9 (-4.3, -1.3)	0.3 (0, 0.7)	0.8 (0.1, 1.6)	-0.7 (-4.4, 2.4)	-5.8 (-6.6, -5.2)
Zimbabwe	6.7 (6, 7.5)	12 (10.9, 13.3)	-16 (-18.4, -13.5)	-0.5 (-1.5, 0.5)	0.2 (0.1, 0.4)	0.2 (0.1, 0.4)	-0.3 (-1.2, 0.7)	-7.8 (-8.5, -7.3)	0.3 (0, 0.6)	0.9 (0.1, 1.9)	-1 (-4.2, 1.8)	-11.9 (-12.6, -11.3)

N/A: estimates not available due to no vaccine introduction in 2016.

.

Table S6.4. Sensitivity analysis – change in the area under the curve for death, compared to Scenario 1, with under-five mortality rate adjustment. None of the cells had greater than 25% difference compared to the main results in Appendix V.

<u>Scenarios</u>

S2: current quintile-specific treatment coverage rates;
S3: national average of treatment coverage for all quintiles;
S4: treatment coverage proportional to quintile-specific baseline mortality risks;
S5: equal baseline mortality risk with current quintile-specific treatment coverage rates.

Country	MCV S2	MCV S3	MCV S4	MCV S5	PCV S2	PCV S3	PCV S4	PCV S5	RV S2	RV S3	RV S4	RV S5
Armenia	3 (0.8, 5.3)	0 (0, 0)	-1.9 (-6.2, 2.8)	-8.7 (-11, -6.1)	2.8 (2.1, 3.6)	0 (0, 0)	-3.8 (-5.7, -1.9)	-12.5 (-14.4, -9.9)	4.6 (3.9, 5.2)	0 (0, 0)	-6.8 (-8.6, -4.9)	-10.2 (-12.5, -7.2)
Burundi	0.5 (0.1, 0.9)	0 (0, 0)	-3.1 (-11.7, 6.4)	-7.9 (-9.5, -6.3)	1 (0.7, 1.3)	0 (0, 0)	-1.3 (-4.3, 2.1)	-1.9 (-4.3, 0.2)	0.6 (0.4, 0.7)	0 (0, 0)	-4.1 (-7.2, -0.9)	-3.1 (-4.5, -1.8)
Benin	1.8 (0.5, 3.1)	0 (0, 0)	-1.5 (-5.7, 3.3)	-6.6 (-7.7, -5.3)	1.8 (1.4, 2.3)	0 (0, 0)	-1.8 (-3.1, -0.4)	-6.1 (-6.5, -5.6)	3.3 (2.8, 3.7)	0 (0, 0)	-4.6 (-5.7, -3.4)	-5.9 (-6.6, -5.3)
Burkina Faso	4.4 (1.1, 8.7)	0 (0, 0)	-2.7 (-9.9, 5.1)	-3.8 (-7.5, 0.9)	6.1 (4.3, 8)	0 (0, 0)	-2.7 (-5.4, 0.1)	0.6 (-1.2, 2.6)	6.6 (5.5, 7.5)	0 (0, 0)	-5 (-6.7, -3.2)	-0.3 (-1.7, 1)
Bangladesh	3.6 (1, 6.3)	0 (0, 0)	-1.7 (-5.5, 2.6)	-5.4 (-8.7, -1.9)	5 (3.7, 6.3)	0 (0, 0)	-1.9 (-3.4, -0.1)	-1.1 (-2.5, 0.4)	5.2 (4.5, 5.8)	0 (0, 0)	-2.5 (-3.2, -1.7)	-2.1 (-3.2, -1.2)
Cote d'Ivoire	2.5 (0.7, 4.5)	0 (0, 0)	-1.8 (-5.8, 2.7)	-7.1 (-9.4, -4.7)	4.6 (3.4, 5.8)	0 (0, 0)	-1.1 (-2.7, 0.8)	0.7 (-0.9, 2.3)	1.8 (1.6, 2)	0 (0, 0)	-2.3 (-3.1, -1.3)	-5 (-6.9, -3.6)
Cameroon	2.9 (0.8, 5.1)	0 (0, 0)	-2.7 (-6.4, 1.5)	-14.8 (-17.5, -11.7)	3.8 (2.9, 4.7)	0 (0, 0)	-4.2 (-6.2, -2.3)	-12.9 (-16.4, -9.1)	4.2 (3.5, 4.7)	0 (0, 0)	-5.5 (-7, -4)	-12.4 (-16.5, -8.6)
DRC	0.2 (0, 0.3)	0 (0, 0)	-2.9 (-7.2, 2.3)	-13.1 (-13.9, -12.4)	0.9 (0.6, 1.1)	0 (0, 0)	-2.9 (-4.5, -1.1)	-8.8 (-10.3, -7.2)	-1.1 (-1.3, -0.9)	0 (0, 0)	-4.7 (-5.9, -3.3)	-11.2 (-13, -9.4)
Congo	3.1 (0.8, 6)	0 (0, 0)	-3 (-9, 3.6)	-7.2 (-10.5, -3.4)	5.5 (3.9, 7.2)	0 (0, 0)	-3 (-5.8, 0.2)	-0.7 (-3.8, 2.5)	3.9 (3.3, 4.5)	0 (0, 0)	-4.7 (-6.2, -3)	-4.7 (-7.4, -2.4)
Comoros	-0.1 (-0.2, 0)	0 (0, 0)	-2.5 (-7.8, 3.3)	-9.4 (-10.6, -8.1)	1 (0.7, 1.2)	0 (0, 0)	-3.2 (-5.3, -0.9)	-7.8 (-9.8, -5.2)	-0.3 (-0.4, -0.2)	0 (0, 0)	-4.5 (-6.2, -2.6)	-7.9 (-9.7, -5.6)
Ethiopia	4 (1.1, 7)	0 (0, 0)	-1.9 (-5.6, 2.2)	-5.9 (-9.2, -2)	5.4 (4, 6.7)	0 (0, 0)	-2.1 (-3.4, -0.7)	-2.9 (-5.4, 0.3)	6.3 (5.3, 7.2)	0 (0, 0)	-3.9 (-5, -2.4)	-1.9 (-4.4, 0.8)
Ghana	-1.8 (-3.4, -0.4)	0 (0, 0)	-2.5 (-9.3, 4.9)	-10.2 (-12, -8.4)	-0.9 (-1.1, -0.6)	0 (0, 0)	-1.5 (-4.9, 1.8)	-4.4 (-7.5, -1.4)	-3.1 (-3.5, -2.6)	0 (0, 0)	-2.9 (-5.5, -0.3)	-6.8 (-9.4, -4.3)
Guinea	4.8 (1.3, 8.9)	0 (0, 0)	-2.4 (-7, 3)	-6.7 (-11.2, -1.4)	7.9 (5.9, 10)	0 (0, 0)	-3.3 (-5.3, -1.3)	-1.3 (-4.4, 1.9)	6 (5.1, 6.9)	0 (0, 0)	-5.9 (-7.6, -4.2)	-4.5 (-6.8, -2.1)
Gambia	-0.9 (-2, -0.2)	0 (0, 0)	-1.2 (-11.2, 9.3)	-5.9 (-7.4, -4.7)	-1.2 (-1.7, -0.8)	0 (0, 0)	0 (-3.2, 4)	-2.1 (-2.8, -1.4)	-1.3 (-1.6, -1)	0 (0, 0)	1.2 (-1.9, 4.9)	-0.9 (-1.6, 0)
Honduras	1 (0.2, 2.1)	0 (0, 0)	-1.7 (-10.8, 8.6)	-8.9 (-10.1, -7.5)	2.5 (1.7, 3.4)	0 (0, 0)	1.9 (-1.8, 6.5)	3.3 (-0.1, 5.9)	0.1 (0.1, 0.2)	0 (0, 0)	0.3 (-3.7, 3.6)	-0.7 (-4.5, 1.9)
Haiti	3.6 (0.9, 6.5)	0 (0, 0)	-0.7 (-4.8, 4.2)	-3.1 (-6, 0.2)	5 (3.7, 6.3)	0 (0, 0)	-0.4 (-1.9, 1.3)	1.9 (0.3, 3.5)	4.9 (4.1, 5.5)	0 (0, 0)	-2.6 (-4, -1.2)	-1.1 (-3.7, 0.9)

Indonesia	1.5 (0.3, 3.6)	0 (0, 0)	-4.8 (-18.3, 8.9)	-10.7 (-11.8, -9.2)	3 (2, 4.4)	0 (0, 0)	-4.3 (-9.7, 1.5)	-3.7 (-5, -2.3)	-0.4 (-0.5, -0.3)	0 (0, 0)	-9 (-13.2, -4.4)	-7 (-8.5, -5.6)
Kenya	0.1 (0, 0.1)	0 (0, 0)	-5 (-16, 6.2)	-9.2 (-10.4, -8.2)	1.4 (0.9, 1.9)	0 (0, 0)	-5.9 (-10, -1.7)	-6.8 (-8.3, -5.6)	-2 (-2.4, -1.6)	0 (0, 0)	-11.5 (-15.5, -8)	-11.5 (-13, -10.5)
Kyrgyz Republic	4.8 (1.1, 9.8)	0 (0, 0)	-0.6 (-7.9, 7.8)	1.2 (-2.8, 6.6)	5.3 (3.8, 6.9)	0 (0, 0)	-0.7 (-3.1, 2.2)	3 (0.5, 5.7)	9 (7.4, 10.4)	0 (0, 0)	-3.1 (-5.3, -0.8)	6.1 (3.8, 8.3)
Cambodia	-1 (-2.3, -0.2)	0 (0, 0)	-3.6 (-13.6, 7.1)	-12.8 (-14.5, -11.5)	0.1 (0, 0.1)	0 (0, 0)	-2 (-6.4, 3.1)	-3.9 (-5.8, -1.4)	-5.2 (-6.1, -4.2)	0 (0, 0)	-3.2 (-6.9, 0.8)	-8.4 (-11.2, -5.5)
Liberia	-0.2 (-0.4, 0)	0 (0, 0)	-2.8 (-9.7, 4.6)	-8.6 (-9.3, -8.1)	-1.4 (-1.8, -1)	0 (0, 0)	-1.9 (-4.5, 1)	-5.8 (-7.6, -3.9)	1.3 (1.1, 1.5)	0 (0, 0)	-3.6 (-5.8, -1.3)	-3.4 (-5.3, -1.5)
Lesotho	-0.6 (-1.2, -0.1)	0 (0, 0)	-4.3 (-13.1, 5.3)	-8.9 (-11.3, -6.9)	0.4 (0.3, 0.6)	0 (0, 0)	-4.9 (-9.6, -0.4)	-6.5 (-10.9, -2.8)	-2.7 (-3.1, -2.2)	0 (0, 0)	-11.3 (-16, -7.3)	-13.6 (-17.2, -10.7)
Mali	3.3 (0.9, 5.9)	0 (0, 0)	-2 (-5.5, 2)	-7.5 (-10.4, -4.4)	4.9 (3.7, 6.2)	0 (0, 0)	-2.3 (-3.5, -0.8)	-3.5 (-5.3, -1.7)	4.5 (3.9, 5)	0 (0, 0)	-3.7 (-4.5, -2.7)	-4.8 (-6.2, -3.3)
Mozambique	2.8 (0.7, 5.6)	0 (0, 0)	-6.3 (-15.7, 3.1)	-11.4 (-13.9, -7.9)	3.4 (2.5, 4.5)	0 (0, 0)	-7.2 (-10.5, -4.1)	-10.5 (-12.4, -8.3)	5 (4.1, 5.9)	0 (0, 0)	-15.9 (-20.5, -11.3)	-8.2 (-10.2, -6.2)
Malawi	0.9 (0.2, 2.2)	0 (0, 0)	-5.9 (-17.5, 6.1)	-9.5 (-11.4, -7.1)	2.7 (1.9, 3.8)	0 (0, 0)	-10.4 (-16.3, -5.1)	-10.5 (-13.7, -6.3)	-1.4 (-1.6, -1.1)	0 (0, 0)	-18.3 (-24.1, -12.7)	-14.5 (-16.9, -11.6)
Niger	4.3 (1, 8.6)	0 (0, 0)	-3.3 (-11.4, 5.6)	-2.8 (-6.3, 1.8)	7.7 (5.5, 10.1)	0 (0, 0)	-4 (-6.9, -0.9)	0.7 (-1.6, 3.1)	3.6 (3, 4.2)	0 (0, 0)	-7.5 (-9.7, -5.1)	-4.6 (-5.3, -3.9)
Nigeria	3.1 (0.8, 5.5)	0 (0, 0)	-2.4 (-6.5, 2.2)	-9.8 (-12.7, -6.7)	4.6 (3.4, 5.8)	0 (0, 0)	-2.8 (-4.5, -1)	-4.3 (-5.8, -2.8)	4.4 (3.9, 4.9)	0 (0, 0)	-3.3 (-4.1, -2.3)	-4.4 (-5.3, -3.5)
Nepal	1 (0.3, 1.9)	0 (0, 0)	-2.1 (-6.5, 3.1)	-9.6 (-12, -7.6)	2.1 (1.5, 2.6)	0 (0, 0)	-0.7 (-2.5, 1.4)	-1 (-3.7, 1.6)	1.7 (1.5, 1.9)	0 (0, 0)	-2.8 (-4.3, -1.3)	-4.4 (-6.7, -2.4)
Pakistan	3.8 (0.8, 8.4)	0 (0, 0)	-8.1 (-21.9, 5.1)	-9.7 (-13.4, -4)	4.6 (3.2, 6.3)	0 (0, 0)	-8.2 (-12.8, -3.3)	-5.6 (-7.5, -3.6)	8.2 (6.6, 9.8)	0 (0, 0)	-16.6 (-21.1, -12.1)	-2.1 (-4.4, 0)
Rwanda	3.3 (0.7, 6.8)	0 (0, 0)	-3.1 (-12.1, 6.4)	-5.4 (-8.4, -1.2)	5.4 (3.7, 7.5)	0 (0, 0)	-2.8 (-7.3, 2)	1 (-1.3, 3.4)	5.8 (4.8, 6.7)	0 (0, 0)	-5.3 (-7.8, -2.5)	-0.1 (-2, 1.5)
Senegal	-0.4 (-0.8, -0.1)	0 (0, 0)	-1.9 (-6.4, 3.4)	-12.9 (-14.3, -11.5)	-1.6 (-2, -1.1)	0 (0, 0)	-1.8 (-3.8, 0.4)	-8.2 (-10.7, -5.4)	1 (0.8, 1.1)	0 (0, 0)	-1.3 (-2.5, 0.1)	-2.7 (-5, -0.4)
Sierra Leone	1.4 (0.3, 3.3)	0 (0, 0)	-2.5 (-12.8, 8.7)	-3.6 (-5.1, -1.5)	1.2 (0.8, 1.6)	0 (0, 0)	-1.7 (-5.6, 3)	-0.9 (-1.9, 0.3)	3.7 (2.9, 4.5)	0 (0, 0)	-5.3 (-8.7, -1.3)	0.1 (-1.2, 1.4)
Chad	3.1 (0.9, 5.4)	0 (0, 0)	0 (-2.7, 3)	1.9 (-0.6, 4.5)	4 (3.1, 5)	0 (0, 0)	0.2 (-0.7, 1.4)	5.1 (4, 6.2)	4.3 (3.8, 4.8)	0 (0, 0)	-0.3 (-0.9, 0.4)	3.4 (2.3, 4.3)
Togo	1.1 (0.3, 2.1)	0 (0, 0)	-1.9 (-6.1, 3.3)	-10.3 (-12, -8.3)	2.5 (1.8, 3.2)	0 (0, 0)	-2.8 (-5.1, -0.6)	-5.3 (-6.5, -4.1)	0.7 (0.5, 0.9)	0 (0, 0)	-3.8 (-4.6, -2.9)	-10.9 (-12.3, -9.7)
Tajikistan	1.8 (0.4, 3.6)	0 (0, 0)	-0.6 (-8.6, 8.2)	-5.1 (-6.4, -3.4)	3 (2.1, 4)	0 (0, 0)	-0.8 (-3.9, 2.8)	0.6 (-1, 2.6)	3.5 (2.9, 4.1)	0 (0, 0)	-1.3 (-4.2, 1.9)	2 (0.3, 4)
Timor-Leste	1.9 (0.4, 4.6)	0 (0, 0)	-4.2 (-17.2, 8.8)	-5.2 (-6.9, -2.3)	3.4 (2.3, 4.8)	0 (0, 0)	-4.2 (-8.5, 0.6)	-1.8 (-3.5, 0.2)	4.6 (3.5, 5.7)	0 (0, 0)	-10.4 (-14.9, -4.5)	-0.7 (-2.4, 1.1)
Tanzania	1.2 (0.3, 2.5)	0 (0, 0)	-3.3 (-11.7, 5.6)	-6.5 (-8, -4.7)	3.1 (2.1, 4.2)	0 (0, 0)	-1.7 (-5.2, 2.2)	0.3 (-1.6, 2.1)	2.9 (2.4, 3.4)	0 (0, 0)	-3.7 (-7.2, -0.6)	-0.5 (-2.9, 1.6)
Uganda	-0.3 (-0.9, -0.1)	0 (0, 0)	-2.6 (-15.5, 10.7)	-7.2 (-8.3, -6.3)	0.5 (0.3, 0.7)	0 (0, 0)	-2 (-6.8, 3.6)	-2.3 (-3.4, -1)	-2.6 (-3.3, -1.9)	0 (0, 0)	-8.4 (-13.5, -3.4)	-7.8 (-9.7, -6.1)
Yemen	1.2 (0.3, 2.2)	0 (0, 0)	-2.9 (-7.4, 2.2)	-11 (-12.5, -9.5)	1.5 (1.1, 1.9)	0 (0, 0)	-2.7 (-4.3, -0.9)	-7.5 (-8.6, -6.2)	2.3 (2, 2.6)	0 (0, 0)	-4.3 (-5.5, -2.9)	-6.4 (-7.6, -5)

Zambia	1.4 (0.3, 3.2)	0 (0, 0)	-4.1 (-17, 9.6)	-6.7 (-8.1, -4.6)	3.2 (2.1, 4.5)	0 (0, 0)	-3.3 (-8, 1.8)	-1 (-2.4, 0.6)	0 (-0.1, 0.1)	0 (0, 0)	-7.7 (-11.7, -3.4)	-5.3 (-6.5, -4.1)
Zimbabwe	-0.3 (-0.7, -0.1)	0 (0, 0)	-1.7 (-6.8, 4)	-8.7 (-9.2, -8.2)	-1.4 (-1.8, -1)	0 (0, 0)	-2.9 (-5, -0.6)	-10.4 (-11.3, -9.6)	0.4 (0.3, 0.4)	0 (0, 0)	-4.6 (-5.8, -3.3)	-10.1 (-11.5, -9.1)

VII. References

1. USAID, ICF Macro International. Demographic and Health Surveys (DHS) Program. [cited 2016 Oct 15]. Available from: http://dhsprogram.com.

2. GBD 2013 Risk Factors Collaborators. Global, regional, and national comparative risk assessment of 79 behavioural, environmental and occupational, and metabolic risks or clusters of risks in 188 countries, 1990–2013: a systematic analysis for the Global Burden of Disease Study 2013. The Lancet. 2015;386(10010):2287–2323.
